# Nature‐Inspired Surface Modification Strategy Reverses the Autophagic Flux Impairment of Mitochondrial Transplantation for Attenuating Ischemic Strokes

**DOI:** 10.1002/advs.202518969

**Published:** 2026-02-25

**Authors:** Nisha Wang, Qiyang Ding, Lei Shi, Ji Xia, Shuyue Zhang, Chenxin Jian, Yixiao Yan, Xiuhua Luo, Jiarui Wang, Ming Cheng, Yiqiong Jia, Hao Tian, Wei Gao

**Affiliations:** ^1^ Department of Anesthesiology The First Affiliated Hospital of Xi'an Jiaotong University Xi'an China; ^2^ Shaanxi University of Chinese Medicine Xianyang China; ^3^ Center for Brain Science The First Affiliated Hospital of Xi'an Jiaotong University Xi'an China

**Keywords:** autophagic flux, mitochondrial transplantation, mitophagy, ROS‐RIP1/RIP3‐exosome axis

## Abstract

Mitochondrial transplantation has emerged as a promising therapeutic intervention for ischemic strokes (IS). Although previous studies have demonstrated the therapeutic breakthroughs of mitochondrial transplantation facilitated by advances in biotechnology, in‐depth investigations into the exact mechanisms underlying its beneficial effects remain insufficient. Here, we investigate how exogenous mitochondria interact with recipient cells to optimize therapeutic protocols and improve outcomes. Emerging evidence indicates that exogenous mitochondria act as triggers of mitophagy via the PTEN‐induced putative kinase 1 (PINK1)–Parkin pathway. However, excessive reactive oxygen species (ROS) generated during ischemia‐reperfusion injury activate the receptor‐interacting protein (RIP)1/RIP3 pathway, leading to the blockage of autophagic flux. Hence, we devised a novel mitochondrial transplantation platform (MLSR) that utilizes functionalized starch as a stable coating for exogenous mitochondria and enables the co‐delivery of the antioxidant resveratrol through the helical structure of the starch. Following internalization by recipient neurons, the exogenous mitochondria rapidly initiate mitophagy, while resveratrol escapes from the lysosome to inhibit the ROS‐RIP1/RIP3‐exosome axis. Experimental results demonstrate that MLSR effectively triggers and maintains positive autophagic flux, thereby suppressing the release of undegraded autophagosomes in the form of exosomes and preventing proinflammatory crosstalk between neurons and microglia. Therefore, our findings provide important implications for renewing the therapeutic potential of mitochondrial transplantation.

## Graphical abstract

The functionalized starch modifies the mitochondrial surface and encapsulates resveratrol into the cavity of helices, facilitating a novel mitochondrial transplantation platform (MLSR). Upon internalization in recipient cells, MLSR triggers mitophagy and maintains positive autophagic flux through RIP1/RIP3 pathway inhibition. Our findings provide implications for renewing the therapeutic potential of mitochondrial transplantation.

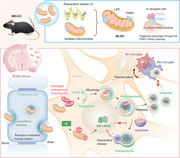

## 1 Introduction

The direct transplantation of healthy exogenous mitochondria into ischemic tissues has emerged as a promising strategy to ameliorate ischemia‐reperfusion (IR) injury and promote functional recovery after stroke [1, 2]. Recent breakthroughs in mitochondrial transplantation have been driven by biotechnological strategies including surface modification, exosome encapsulation, and liposome delivery, which focus on enhancing targeted delivery while preserving mitochondrial activity and facilitating integration into recipient mitochondrial networks [3, 4, 5, 6]. However, the mechanism underlying mitochondrial transplantation remains controversial, as tracking studies have shown the direct delivery of exogenous mitochondria into lysosomes for degradation [7]. Moreover, the mechanisms through which a small number of exogenous mitochondria produce substantial beneficial effects remain elusive [8, 9].

Emerging evidence suggests that the internalization of exogenous mitochondria triggers the transient activation of mitophagy via the PINK1–Parkin signaling pathway [10]. Mechanistically, the presence of PINK1 in donor mitochondria recruits cytoplasmic Parkin in recipient cells, which facilitates the degradation of dysfunctional mitochondria through mitophagy activation. However, by activating the RIP1/RIP3 pathway, the burst production of reactive oxygen species (ROS) following IR injury blocks autophagosome–lysosome fusion, which arrests autophagic flux [11, 12, 13]. In the case of a mitophagy defect, undegraded autophagosomes can be released in the form of exosomes containing mitochondria‐derived cargo, which act as danger‐associated molecular patterns (DAMPs) and trigger proinflammatory crosstalk between neurons and microglia [14]. Therefore, inhibition of the ROS‐RIP1/RIP3‐exosome axis has the potential to restore autophagic flux for mitochondrial transplantation and inhibit microglia activation to an inflammatory phenotype, making it a prospective therapeutic strategy for ischemic strokes.

The co‐delivery of mitochondria with protective molecules represents a promising strategy, and sugars are well‐known bioprotectants owing to their ability to preserve biological functions and structures under extreme conditions [15, 16]. Starch, a biocompatible and biodegradable polysaccharide with negligible toxicity, is an attractive option for constructing surface‐coating materials [17]. Moreover, hydrogen bonding, carbohydrate–aromatic interactions, and hydrophobic interactions collectively enable starch to adopt a defined helical secondary structure capable of co‐delivering hydrophobic molecules within the helix cavity, indicating its significant potential for establishing a novel mitochondrial transplantation platform via mitochondrial surface modification [18, 19, 20].

**FIGURE 1:**
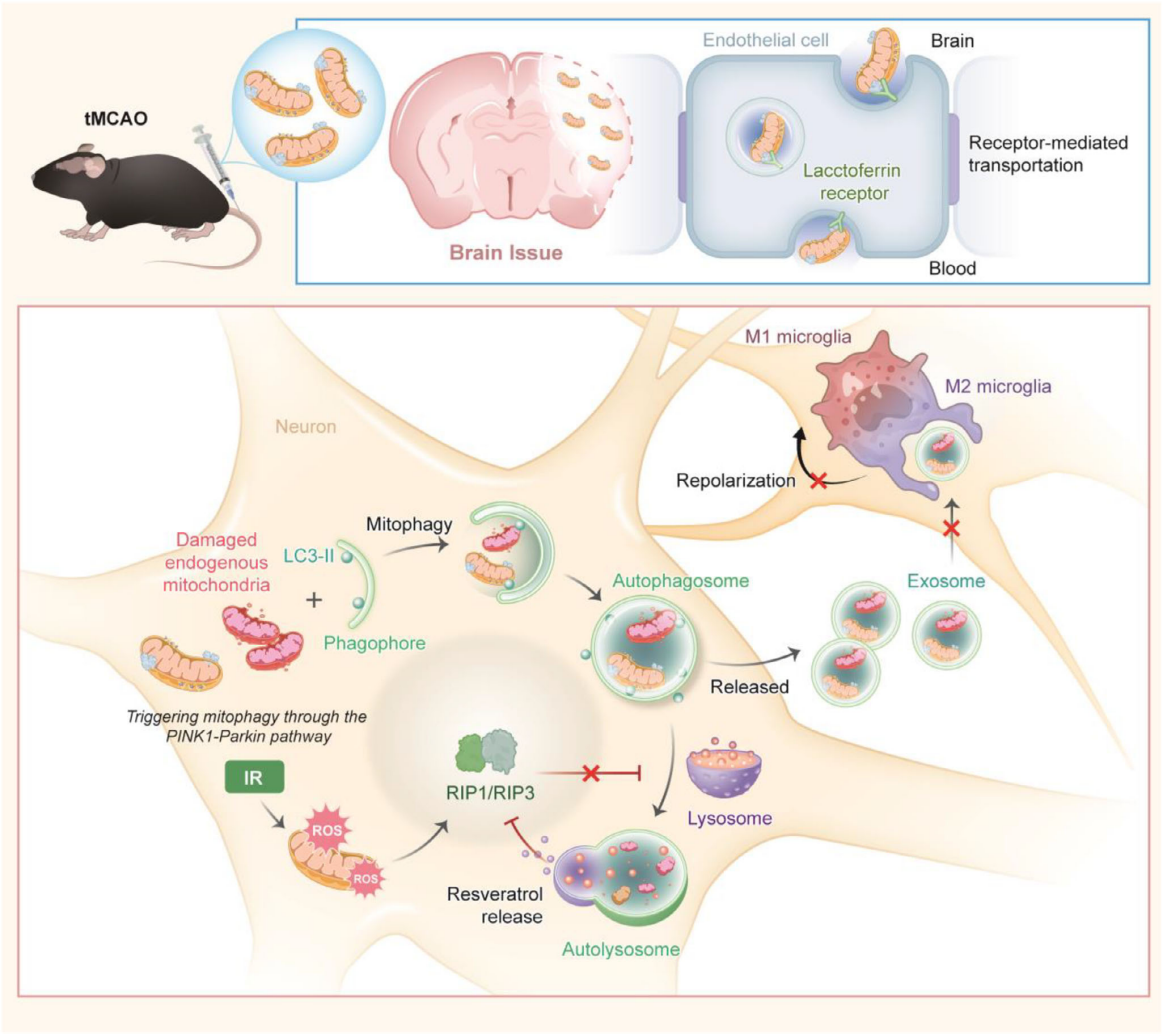
Schematic illustration of the MLSR reverses autophagic flux impairment of mitochondrial transplantation in attenuating ischemic stroke. We established a novel mitochondrial transplantation platform (MLSR) through the surface modification of exogenous mitochondria, which enabled receptor‑mediated transcytosis across the BBB. Upon internalization in recipient cells, MLSR triggers mitophagy via the PINK1–Parkin signaling pathway for neuroprotection. Meanwhile, resveratrol escapes from lysosomes for ROS clearance and restores mitophagy through inhibiting the RIP1/RIP3 pathway, which maintains positive autophagic flux for suppressing the neuro‐microglial proinflammatory communication.

Here, we report a rationally designed mitochondrial transplantation platform (MLSR) that employs functionalized starch as a stable coating for exogenous mitochondria, inspired by natural sugar‐membrane interactions (Figure 1). Upon internalization in recipient cells, we observed that exogenous mitochondria co‐localize with autophagosomes and serve as a trigger for mitophagy via the PINK1–Parkin signaling pathway. Resveratrol is loaded into the helix of starch and escapes from the lysosome for ROS clearance, restoring mitophagy by inhibiting the RIP1/RIP3 pathway. The positive autophagic flux facilitates the transition of autophagosomes to autolysosomes, thereby suppressing the release of damaged mitochondrial components that contribute to proinflammatory crosstalk. Moreover, we chemically modify lactoferrin (Lf) into a starch coating, enabling receptor‑mediated transcytosis across the blood–brain barrier (BBB) [21, 22]. Collectively, the targeted delivery of MLSR to the ischemic penumbra in the transient middle cerebral artery occlusion (tMCAO) model mice enhances neuroprotection, reduces inflammation, promotes long‐term functional recovery, and highlights the importance of maintaining positive autophagic flux during mitochondrial transplantation.

## 2 Results and Discussion

### 2.1 Synthesis and Molecular Dynamics Simulations of Resveratrol‐Loaded Starch

**FIGURE 2:**
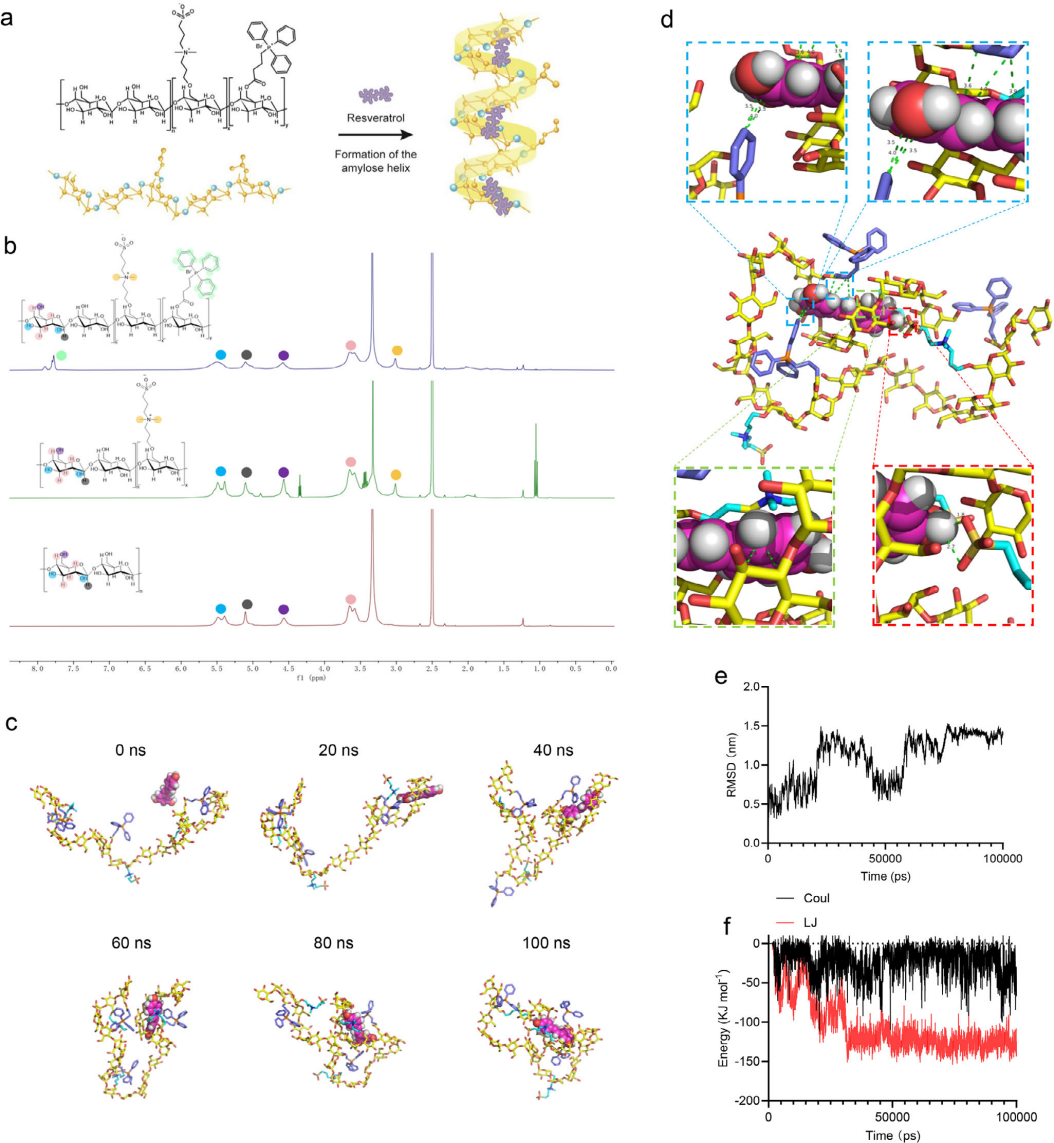
Synthesis and molecular dynamics simulations of resveratrol‐loaded starch (SR). (a) Schematic illustration of the helical structure of Z‑Starch‐TPP and the formation of the SR. (b) Comparison of the ^1^H NMR spectra of the Starch, Z‑Starch, and Z‑Starch‐TPP in DMSO‐d6. (c) Representative snapshots of molecular dynamics simulation for SR at the indicated time points. (d) Snapshot of interactions between Z‑Starch‐TPP and resveratrol at a simulation time of 100 ns. The enlarged view highlighted the hydrophobic interactions within SR via TPP (blue box) and via the sugar ring (green box), and hydrogen‐bonding interactions via sulfonate (red box). (e) Time‐dependent changes in RMSD for SR. (f) Time‐dependent changes of the electrostatic and van der Waals interactions within SR.

Grafted starch (Z‐Starch‐TPP) was synthesized with a helical secondary structure through a two‐step chemical modification to form hydrophobic cavities for establishing drug‐delivery systems (Figure S1; Figure 2a). Specifically, zwitterionic starch (Z‑Starch) was first prepared by etherifying native starch with 1‑chloro‑3‑dimethylaminopropane (CDMAP), which is a water‑soluble side chain. Subsequent esterification of Z‐Starch with triphenylphosphonium (TPP) moieties yielded the final product (Z‑Starch‑TPP). The chemical structures of the intermediate (Z‐Starch) and final product were analyzed by ^1^H NMR spectroscopy (Figure 2b). Z‐Starch exhibited a new methyl hydrogen peak at 3.01 ppm that was consistent with CDMAP incorporation, along with the characteristic peaks from the glucose units in native starch that ranged from 3.5 to 5.5 ppm. Additional characteristic peaks corresponding to the TPP moieties were also observed for Z‐Starch‐TPP. Zeta potential analysis further confirmed the successful conjugation of TPP, as the analysis showed a shift to a positive surface charge (Figure S2). Considering the negative surface potential (−20 to −180 mV) of mitochondria, the TPP modification is expected to facilitate the interactions between Z‑Starch‑TPP and the mitochondrial outer membrane, thereby establishing a stable mitochondrial coating.

Theoretically, a network of intramolecular hydrogen bonds between the O3, O5, O2, and O6 atoms of adjacent glucose units stabilizes the amylose chains of starch into single‐stranded helices [20, 23]. Therefore, the formation of a unique helical secondary structure in Z‐Starch‐TPP was verified by mixing it with a 3% iodine solution. Upon binding to starch in all solutions, iodine complexation generated a characteristic deep blue color, indicating that chemical modification had no effect on the formation of the helical structure of starch (Figure S3). Similarly, resveratrol, a polyphenolic antioxidant with known ROS scavenging properties, was effectively encapsulated in a helical cavity to yield resveratrol‐loaded starch (SR). Drug loading was verified by ultraviolet (UV)‐visible spectroscopy using the characteristic peak of resveratrol, and the loading content was calculated to be approximately 13.8% (Figure S4). Release studies were performed under physiological (pH 7.4) and lysosome‐mimicking (pH 5.5) conditions. Results showed that there was only a minimal release of resveratrol of approximately 20% within 24 h under physiological conditions. By contrast, the release rate was accelerated under lysosomal conditions, with more than 50% of the resveratrol released within 12 h (Figure S5).

To better understand the formation process of SR and the interactions between Z‑Starch‑TPP and resveratrol, we conducted a molecular dynamics (MD) simulation in which 16,698 water molecules and one resveratrol molecule and Z‐Starch‐TPP (the ratio of residual glucose, CDMAP, and TPP was 6:1:1, respectively) were randomly positioned in an 8 × 8 × 8 nm simulation box. During the simulation, data were saved every 20 for 100 ns. According to snapshots of the conformation captured at various time points, Z‐Starch‐TPP was fully folded and encapsulated in resveratrol at approximately 60 ns, and this process was completed at 80 ns (Figure 2c). The simulations demonstrated that this process involved several interactions, including hydrophobic interactions between resveratrol and the sugar ring. Meanwhile, the hydroxyl groups of resveratrol and sulfonate groups on the side chains of starch formed hydrogen‐bonds. In particular, the hydrophobic effect of TPP on the starch side chains further accelerated the SR folding (Figure 2d). Following a 100 ns MD simulation, resveratrol was encapsulated in the helical cavity of starch (Figure S6). Root mean square deviation (RMSD) analysis further confirmed the folding process, in which the encapsulation of resveratrol in the helices reached equilibrium at 80 ns (Figure 2e). Furthermore, the energy shift during the folding process indicated that hydrophobic interactions were necessary for the encapsulation of resveratrol in the helical cavity, even though the starch and its side chains contain a high level of static electricity (Figure 2f).

### 2.2 Construction and Characterization of SR‐Coated Mitochondria

**FIGURE 3:**
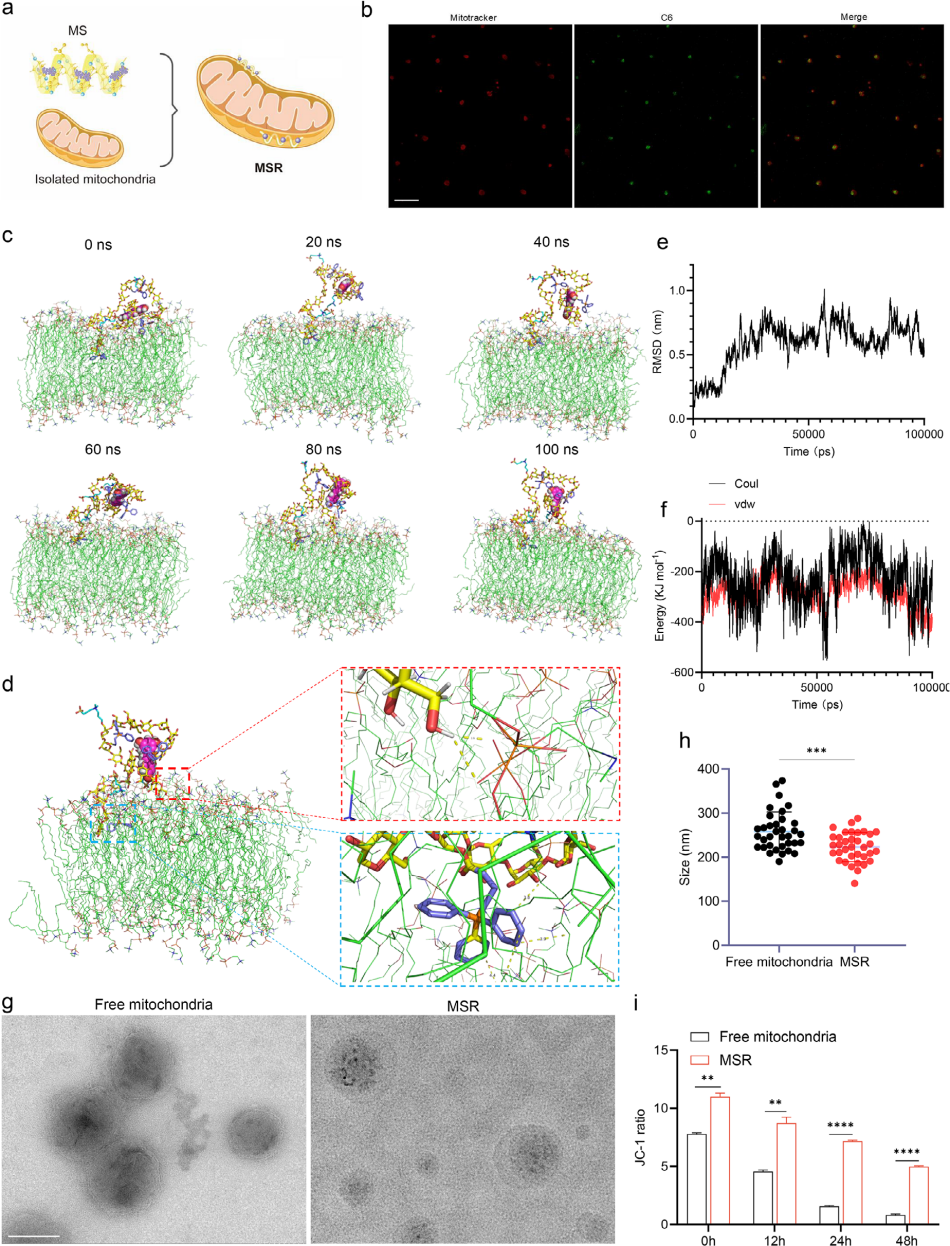
Construction and characterization of SR‐coated mitochondria (MSR) (a) Schematic illustration of the construction of MSR. (b) Confocal laser scanning microscope (CLSM) images of MitoTracker‐stained mitochondria (red) and C6‐labeled SR (green). Scale bars: 25 µm. (c) Representative snapshots of molecular dynamics simulation of the interactions between SR and the mitochondrial outer membrane. (d) Snapshot of interactions between SR and the mitochondrial outer membrane at a simulation time of 100 ns. The enlarged view highlighted the hydrophobic interactions (blue box) and hydrogen‐bonding interactions (red box). (e) Time‐dependent changes in RMSD for SR during the interactions with the mitochondrial outer membrane. (f) Time‐dependent changes of the electrostatic effect and the Van der Waals effect. (g) TEM images of unmodified mitochondria and MSR. Scale bars: 200 nm. (h) Size distribution of unmodified mitochondria and MSR (t‐test; n  =  35 per group). (i) Quantitative analysis of unmodified mitochondria and MSR for mitochondrial membrane potential after staining with JC‐1 (t‐test; n  =  3 per group). Data are presented as mean ± s.d., and n represents biological replicates. ^*^ *p* <0.05; ^**^ *p* <0.01; ^***^ *p* <0.001; ^****^ *p* <0.0001 are considered as statistically significant.

Platelets, which are abundant in peripheral blood and readily obtainable through minimally invasive venipuncture, were used to isolate mitochondria. The isolated mitochondria were quantified using flow cytometry, and the concentration was estimated to be approximately 2.29 × 10^10^ mL^−1^ (Figure S7). Subsequently, the SR was mixed with the mitochondrial suspension to obtain surface‐modified mitochondria (MSR; Figure 3a). The fluorescence co‐localization of C6‐labeled SR (green) and MitoTracker‐stained mitochondria (red) supported the successful formation of the MSR (Figure 3b). The TPP moieties from the side chains of starch were positively charged and lipophilic components, which are considered to facilitate the interactions with the negatively charged mitochondrial membrane. Therefore, we further utilized MD simulations of the interactions between the SR and mitochondrial membrane to verify the above modification process. In the simulation system, the mitochondrial outer membrane was simulated by establishing a lipid bilayer that was composed of 128 1‐palmitoyl‐2‐oleoyl‐sn‐glycero‐3‐phosphocholine (POPC) molecules and three cardiolipin (CL) molecules [24, 25]. Then, 16 581 water molecules and six Na^+^ molecules were added to maintain electrical neutrality, and the SR was pulled along the direction perpendicular to the interior of the mitochondrial membrane. During the 100 ns molecular simulation, we observed that the sugar chains from the SR were always inserted into the membrane (Figure 3c). A snapshot taken at a simulation time of 100 ns showed that the interactions between the SR and mitochondrial membrane were hydrophobic interactions via TPP moieties, and hydrogen‐bonding interactions (Figure 3d). Moreover, variations in the RMSD of the SR demonstrated that it tended to stabilize after 30 ns; however, the presence of the mitochondrial membrane led to fluctuations in the structure of the SR (Figure 3e). Concurrently, the energy changes in the simulation validated the significance of the van der Waals effect, which was marginally stronger than the electrostatic effect, indicating that the TPP moieties facilitated the mitochondrial surface modification (Figure 3f).

This modification process is expected to support the long‐term storage of mitochondria and preserve mitochondrial stability. Transmission electron microscopy (TEM) directly revealed a coating layer surrounding the mitochondria in the MSR group that was absent in free mitochondria. The size distribution indicated that the average size of MSR was smaller than that of the unmodified mitochondria (Figure 3g,h). To confirm the structural stability of MSR during long‐term preservation, JC‐1 assay was employed to evaluate the changes in mitochondrial membrane potential (MMP) at different time points. Compared with the rapid decline of MMP in the free mitochondrial group, the MSR group remained the MMP at a high level even after 48 h of storage (Figure 3i). Moreover, the structural stability following the successful surface modification was further confirmed through the protease‐shaving assay. According to the results, the MSR group exhibited a good protective effect under the enzymatic degradation. Only 14.6% of total mitochondrial protein was degraded at 3 h, and slightly more than 50% at 6 h. The protective effect of surface modification on critical mitochondrial proteins (PINK1) was also confirmed through Western blot analysis (Figure S8). As mitochondria are essential organelles for energy production, ATP synthesis, and mitochondria complex I activity were also assessed as functional indicators of mitochondrial bioactivity after 24 and 48 h of preservation (Figure S9). The ATP production capacity of the free mitochondria decreased significantly after 24 h of preservation. By contrast, the mitochondria in the MSR group retained a substantially higher ATP production capacity after being preserved for 48 h, with the specific value being approximately one‐third that of freshly isolated mitochondria (0 h). Similarly, the MSR group performed better in preserving the biological functions of complex I, as evidenced by the amount of NAD^+^ produced at approximately 260 nm (Figure S9). These findings demonstrate that starch‐based surface modification confers meaningful preservation of mitochondrial bioactivity during storage.

### 2.3 MSR Triggered Mitophagy Through the PINK1–Parkin Signaling Pathway

**FIGURE 4:**
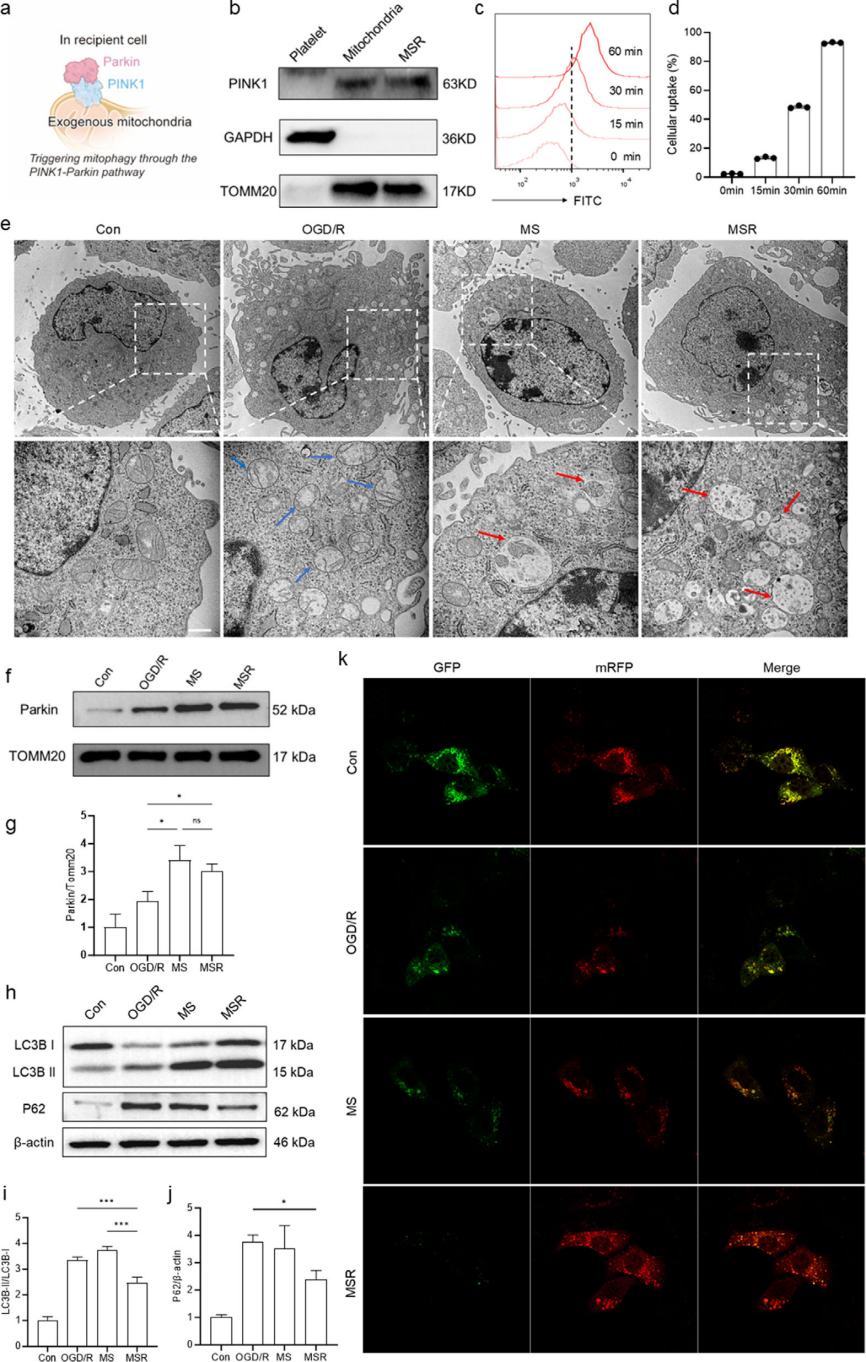
MSR triggered mitophagy through the PINK1–Parkin signaling pathway. (a) Schematic illustration of exogenous mitochondria initiating mitophagy in the recipient cells. (b) Western blot analysis of the PINK1 protein in platelets, isolated mitochondria, and MSR. (c,d) Flow cytometry analysis of cellular uptake behavior and quantification after co‐incubation with different times (n  =  3 per group). (e) TEM images showed the formation of autophagosomes and autolysosomes in MS and MSR groups, indicating mitophagy activation at 30 min post‐ transplantation. Scale bar: 2 µm. (f,g) Western blot analysis and quantification of Parkin in mitochondria from recipient cells after transplantation (one‐way ANOVA; n  =  3 per group). (h–j) Western blot analysis and quantification of mitophagy‐related proteins at 30 min transplantation (one‐way ANOVA; n  =  3 per group). (k) CLSM images demonstrated autophagic flux in HT22 cells after transfection with the mRFP‐GFP‐LC3 lentivirus. Scale bar: 30 µm. Data are presented as mean ± s.d., and n represents biological replicates. ^*^ *p* <0.05; ^**^ *p* <0.01; ^***^ *p* <0.001; ^****^ *p* <0.0001 are considered as statistically significant.

Mitophagy refers to the selective degradation of damaged mitochondria, a crucial cellular process for reducing IR injury [26]. Emerging evidence suggests that exogenous mitochondria can engage the PINK1–Parkin pathway to initiate mitophagy in recipient cells (Figure 4a). Using western blotting (WB) analysis with TOMM20 as a mitochondrial protein, we first verified the presence of PINK1 in lysates from both platelet‐isolated mitochondria and MSR (Figure 4b; Figure S10). The subsequent uptake behavior was determined by a simple co‐incubation between coumarin 6 (C6)‐labeled MSR and HT22 cells, demonstrating that the cellular internalization of MSR reached over 90% within 1 h via flow cytometry (Figure 4c,d). Following transplantation, the effect of MSR on mitophagy was demonstrated using TEM images of the recipient cells (Figure 4e). Healthy mitochondria in the control group exhibited a high matrix density and clear cristae structure. Conversely, the oxygen‐glucose deprivation/reperfusion (OGD/R)‐treated HT22 cells exhibited swollen mitochondria with broken crista structures (blue arrows). Notably, both MS and MSR transplantation markedly induced the formation of autophagosomes and autolysosomes (red arrows) that were wrapped and degraded around damaged mitochondria, indicating mitophagy activation. Moreover, both mitochondrial transplantation groups showed a significant decrease in mitochondrial swelling, suggesting the protective effects of mitophagy.

Mechanistically, the accumulation of PINK1 on the outer mitochondrial membrane (OMM) selectively recruits Parkin from the cytosol [27]. After collecting mitochondrial lysates from various treatment groups, WB analysis verified the translocation of Parkin to endogenous mitochondria in the recipient cells following transplantation (Figure 4f,g; Figure S11). This finding is consistent with the increase in autophagosome formation observed in both the MS and MSR groups. The efficient progression of mitophagy further involves lysosome‐autophagosome fusion to degrade the enclosed cargo. Static enumeration of autophagosome formation can lead to an inaccurate interpretation of this dynamic and multistage process. To assess the overall autophagic flux, related markers were evaluated by WB analysis (Figure 4h–j). Increased LC3B‐II expression was observed in both the MS and MSR groups, consistent with mitophagy activation (Figure S12). However, p62 accumulated in the MS group, and its expression level was comparable to that in the OGD/R group, indicating a potential blockade of autophagic flux (Figure S13). By contrast, MSR treatment significantly reduced p62 accumulation. Moreover, LC3B‐II on the inner autophagosomal membrane was degraded, and partially delipidated to LC3B‐I and recycled for the formation of new autophagosomes [28, 29]. The MSR‐treated cells exhibited a lower LC3B‐II/LC3B‐I ratio than the MS group, suggesting more complete degradation of substrates within the autophagosomes. Transfection with mRFP‐GFP‐LC3 lentivirus further confirmed these results (Figure 4k). Persistent GFP fluorescence was observed in MS‐treated cells, demonstrating autophagosome accumulation and impaired autophagic flux. MSR treatment facilitated effective autophagosome–lysosome fusion, underscoring the essential role of resveratrol in restoring autophagic flux.

### 2.4 MSR‐Restored Autophagic Flux via Inhibition of the ROS‐RIP1/RIP3 Pathway

**FIGURE 5:**
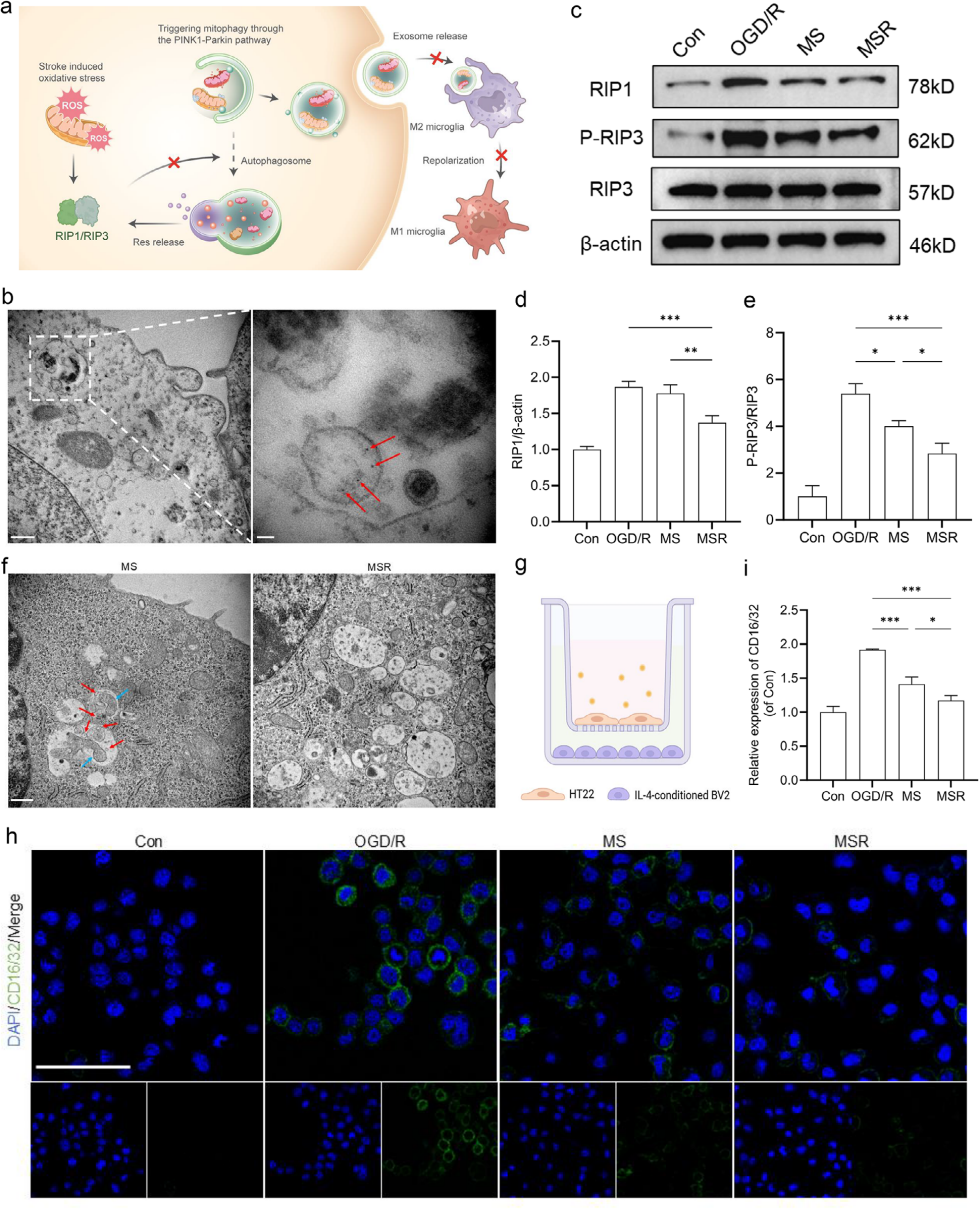
MSR restored autophagic flux via inhibition of the ROS‐RIP1/RIP3 pathway, thereby mediating neuroprotection and suppressing the inflammatory cascade. (a) Schematic illustration of MSR inhibiting the RIP1–RIP3 pathway through ROS clearance, thereby modulating neuro‐microglial proinflammatory communication to reduce post‐stroke inflammation. (b) TEM images showed the co‐localization of Au nanoparticle‐labeled MSR and endosomes following internalization. Scale bar: 500 nm. (c–e) Western blot analysis and quantification of RIP1/RIP3 pathway inhibition. (one‐way ANOVA; n  =  3 per group). (f) TEM images showed the autophagosomes containing undegraded mitochondria and suspected exosomes in the MS group, while damaged mitochondria were completely degraded in the MSR group. Scale bar: 500 nm. (g) Schematic illustration of the transwell model in which HT22 cells and BV2 cells were co‐cultured to evaluate the inhibition of the inflammatory cascade after different treatments. (h) CLSM images showed the changes in CD16/32 (M1 phenotypic marker) expression after the co‐culture model. Scale bar: 50 µm. (i) Quantitative analysis of the expression of CD16/32. (one‐way ANOVA; n  =  3 per group). Data are presented as mean ± s.d., and n represents biological replicates. ^*^ *p* < 0.05; ^**^ *p* < 0.01; ^***^ *p* < 0.001; ^****^ *p* < 0.0001 are considered as statistically significant.

IR injury initiates a succession of events, including the production of excessive ROS that activates the RIP1/RIP3 pathway [11]. This restricts the transition of autophagosomes to autolysosomes, thereby disrupting autophagic flux. We confirmed that resveratrol co‑delivered within the MSR could escape from the endosomal compartments for ROS clearance, which mitigates autophagosome accumulation and modulates neuro‐microglial proinflammatory communication (Figure 5a). MSR were labeled with positively charged Au nanoparticles (red arrows), and TEM analysis demonstrated the early localization of MSR within endosomes following internalization (Figure 5b). Similarly, the CLSM results revealed the co‐localization of C6‐labeled MSR with lysosomes 30 min after incubation with HT22 cells, as evidenced by the extensive yellow fluorescence in the merged images (Figure S14). Additionally, the release of resveratrol and its cytosolic diffusion were indicated by a transition to green fluorescence over time. ROS detection assays demonstrated that this release was functionally associated with a significant decrease in the intracellular ROS levels (Figure S15). Moreover, compared to MS alone, WB analysis revealed that MSR treatment significantly downregulated RIP1 expression (from 1.78 to 1.37) and reduced phosphorylated RIP3 levels (from 4.01 to 2.84), indicating the efficient suppression of the RIP1/RIP3 pathway (Figure 5c–e; Figures S16 and S17). These findings underscore the dual functionality of MSR in inducing mitophagy and restoring autophagic flux through the inhibition of the RIP1–RIP3 pathway.

To establish a strong correlation between the restoration of autophagic flux and the RIP1/RIP3‐exosome axis, we introduced necrostatin‐1 (Nec‐1, RIP1 inhibitor) and necrosulfonamide (NSA, MLKL inhibitor) as mechanistic controls (Figure S18). However, the WB results revealed that neither Nec‐1 nor NSA significantly reduced p62 accumulation or improved the transformation of LC3B‐II/I when compared to the MSR group. Therefore, we hypothesized that the simple inhibition of necroptosis signaling alone is not sufficient to restore autophagic flux and that exogenous mitochondria provide a critical initiating signal via the PINK1–Parkin pathway. Groups that combined exogenous mitochondria with necroptosis inhibitors were included in subsequent experiments (Figure S19). Notably, the Nec‐1+MS group exhibited a marked restoration of autophagic flux, characterized by enhanced p62 reduction and the transformation of LC3B‐II/I, closely resembling the effects observed in the MSR group. However, the NSA+MS group did not show a comparable improvement, which may be attributed to the fact that the target of NAS was the terminal MLKL blockade. Moreover, the culture media from each group were collected to quantify the extracellular mitochondrial DNA (mtDNA) levels (Figure S20). The results demonstrated that similar levels of reduced mtDNA release were observed in the MSR and MS+Nec‐1 groups. By contrast, groups lacking effective flux restoration did not display suppression of mitochondrial cargo release. Reduced mtDNA release was linked to inflammatory consequences in BV2 cells in the transwell model. Both the MSR and MS+Nec‐1 groups showed significantly suppressed M1 polarization of BV2 cells in the lower chamber, as evidenced by the reduced expression of M1‐associated markers (CD86 and iNOS).

Moreover, selective removal of damaged and dysfunctional mitochondria through mitophagy is beneficial for mitochondrial quality control and cellular energy homeostasis. Therefore, the MMP and ATP production in different groups were evaluated for OGD/R‐treated HT22 cells (Figure S21). JC‑1 staining revealed that the MMP levels were significantly reduced in the OGD/R group. By contrast, both MS and MSR transplantation treatments preserved MMP, and the MSR‑treated cells exhibited near‐normal MMP in almost all HT22 cells. Correspondingly, ATP production benefited from the restoration of MMP, suggesting improved mitochondrial function. Because mitophagy is a highly energy‐dependent process, improvements in intracellular bioenergetic homeostasis and ATP production contribute to the initiation of mitophagy, autophagosome maturation, and lysosomal degradation. Therefore, enabling the effective recovery of autophagic flux via MSR also serves as a bioenergetic donor, providing essential ATP to support energy‐demanding autophagic processes.

### 2.5 Positive Autophagic Flux Mediated Neuroprotection and Inflammatory Cascade Inhibition

Mechanistically, mitochondrial dysfunction is associated with apoptosis; therefore, we used annexin V/PI flow cytometry and CCK‐8 assays to investigate cell survival (Figure S22). MS and MSR transplantation mitigated apoptosis in OGD/R‐treated HT22 cells. However, MSR conferred greater neuroprotective effects, which are likely attributable to synergistic mitophagy activation and ROS clearance.

Positive autophagic flux also mitigates neuroinflammation by inhibiting proinflammatory crosstalk between neurons and microglia. In the case of a defect in mitophagy, the secretion of exosomes containing undegraded mitochondria acts as a danger signal to exacerbate post‐ischemic inflammation. TEM images acquired 6 h post‐transplantation revealed the accumulation of autophagosomes containing undegraded mitochondria or mitochondrial fragments (blue arrows) in the MS group as well as the membranous structures of suspected exosomes (red arrows). By contrast, MSR treatment enabled the complete lysosomal degradation of damaged mitochondria, indicative of the terminal step of mitophagy (Figure 5f). Subsequently, we used a transwell model in which HT22 and BV2 cells were co‐cultured to evaluate the inflammatory consequences of the extracellular release of undegraded mitochondrial fragments via exosomes (Figure 5g). OGD/R‐treated HT22 cells were placed in the upper chamber and treated with PBS, MS, or MSR. Simultaneously, BV2 cells were stimulated with IL‐4 to obtain the M2 phenotype and were added to the bottom chamber. After the co‐culture period, the obtained results supported that MSR inhibited proinflammatory crosstalk between neurons and microglia, as evidenced by the minimal changes in CD206 (M2 phenotypic marker) expression (Figure S23). Furthermore, there was minimal expression of CD16/32 (an M1 phenotypic marker), indicating inhibition of the proinflammatory response in microglia (Figure 5h,i). Conversely, the MS group showed an obvious upregulation of CD16/32 expression, and CD206 expression decreased to a certain extent, implying that BV2 cells underwent a polarization process toward a proinflammatory phenotype. These findings indicate that positive autophagic flux via MSR attenuates inflammatory signaling from neurons to microglia and maintains microglial immune homeostasis.

### 2.6 MLSR‐Enabled Brain‐Targeted Mitochondrial Transplantation

**FIGURE 6:**
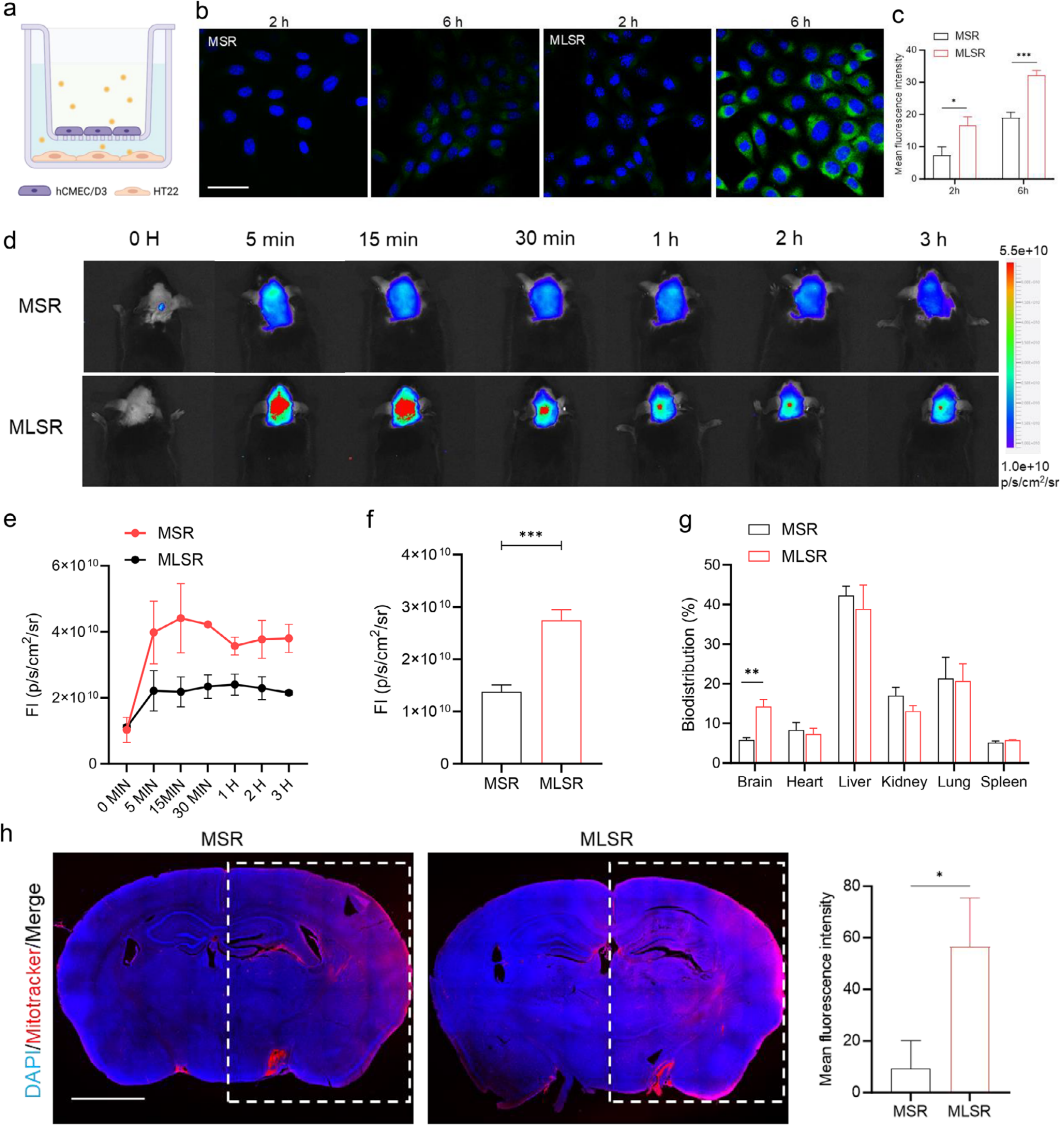
MLSR enabled brain‐targeted mitochondrial transplantation. (a) Schematic illustration of the in vitro BBB model for evaluating BBB penetration of MSR and MLSR. (b) CLSM images showed the enhanced transport efficiency facilitated by Lf modification in the in vitro BBB model. Scale bar: 50 µm. (c) Quantification of fluorescence intensity from the lower chamber of the in vitro BBB model. (n  =  3 per group, t‐test). (d) In vivo fluorescence imaging of Ce6‐labeled MSR and MLSR in the brain region post‐injection. (e) Quantification of the fluorescence intensity in the brain region from the MSR and MLSR group (n  =  3 per group). (f) Quantification of fluorescence intensity from ex vivo brains at 3 h post‐injection. (t‐test; n  =  3 per group). (g) Quantification of fluorescence intensity in ex vivo major organs at 3 h post‐injection (t‐test; n  =  3 per group). (h) CLSM images and the fluorescence quantification showed a greater accumulation of Mitotracker‐labeled MLSR in the ischemic penumbra after intravenous injection (right side). The same imaging parameters, including identical exposure times and Look‐Up Table (LUT) were employed for different groups to ensure fair comparison (t‐test; n  =  3 per group). Scale bar: 2 mm. Data are presented as mean ± s.d., and n represents biological replicates. ^*^ *p* < 0.05; ^**^ *p* < 0.01; ^***^ *p* < 0.001; ^****^ *p* < 0.0001 are considered as statistically significant.

MSR has demonstrated significant therapeutic effects in a series of in vitro results by promoting positive autophagic flux, which induces neuroprotection and suppresses post‐ischemic inflammation. However, because exogenous mitochondria require to be transported across multiple biological barriers, a non‐invasive administration route remains a crucial challenge. To address the challenges of delivering MSR to ischemic brain tissue, SR was chemically modified with brain‐targeting molecules to enhance BBB penetration. Lactoferrin (Lf), an iron‐binding protein that binds receptors (LfR) in the brain, has been utilized as a ligand to facilitate drug delivery into the brain through receptor‐mediated transcytosis [21]. Consequently, a straightforward modification was employed to obtain LSR (Lf‐modified SR), and the mass ratio of Lf was determined to be 8.4% by calculating the protein concentration (Figure S24). In the in vitro BBB model, the resulting MLSR formulation significantly improved BBB penetration. The brain microvascular endothelial cells (HCMEC/D3) were inoculated in the upper chamber and used for subsequent experiments when the trans‐endothelial electrical resistance (TEER) value reached above 200 Ω cm^2^. Subsequently, C6‐labeled MSR or MLSR were added to the upper chamber of the in vitro BBB model, and the fluorescence intensity of HT22 cells in the lower chamber was evaluated using CLSM images (Figure 6a). The enhanced permeability facilitated by Lf modification was confirmed by the fact that MLSR exhibited higher transport efficiency at both 2 and 6 h after incubation compared to the passive transport group (Figure 6b,c).

Near‐infrared fluorescence imaging was used to investigate biodistribution in C57BL/6 mice. Fluorescence quantification in the brain region following the intravenous injection of Ce6‐labeled MSR or MLSR showed that the fluorescence signal from the MLSR group reached a peak at 15 min post‐injection, but only maintained a lower fluorescence signal in the MSR group even after 3 h (Figure 6d,e). Additionally, ex vivo organ imaging further demonstrated that MLSR accumulated approximately 2.45 times more than MSR in the brain, confirming improved brain‐targeted delivery (Figure 6f; Figure S25). Significant fluorescence was also observed in the liver of both groups, indicating that the intravenously injected exogenous mitochondria were predominantly eliminated by the liver (Figure 6g). In addition, exogenous mitochondria were stained with the MitoTracker probe to provide direct evidence of accumulation in the brain (Figure S26). Prior to surface modification, the exogenous mitochondria were thoroughly washed to remove excess and unbound dyes to minimize potential false‐positive fluorescence. Consistent with the previous starch‐labeling results, the fluorescence signals in the brain region from the MLSR group were stronger than those in the MSR group. However, the fluorescence signals from the MitoTracker‐labeled MLSR continued to increase throughout the monitoring period, providing direct evidence that exogenous mitochondria accumulate in the brain, rather than from rapidly clearance.

Moreover, tMCAO model was established to assess the delivery efficiency of MLSR at cerebral ischemic sites (Figure S27). Using Ce6‐labeled MSR or MLSR, brain slices were extracted at 3 h post‐injection. Only a weak fluorescence signal was observed in the cerebral cortex of mice administered with MSR (Figure S28). By contrast, intense fluorescence from the MLSR group was observed in the ischemic penumbra (right side) from the MLSR group, confirming its excellent delivery and accumulation in cerebral ischemic lesions. Correspondingly, the delivery efficiency of Mitotracker‐labeled MSR or MLSR was also assessed in the same imaging parameters, including identical exposure times and Look‐Up Table (LUT). The mean fluorescence intensity was analyzed in the ischemic penumbra (right side), which provided evidence for enhanced brain delivery and retention of exogenous mitochondria (Figure 6h).

### 2.7 MLSR‐Mediated Positive Autophagic Flux Enhanced In Vivo Therapeutic Effects After IR Injury

**FIGURE 7:**
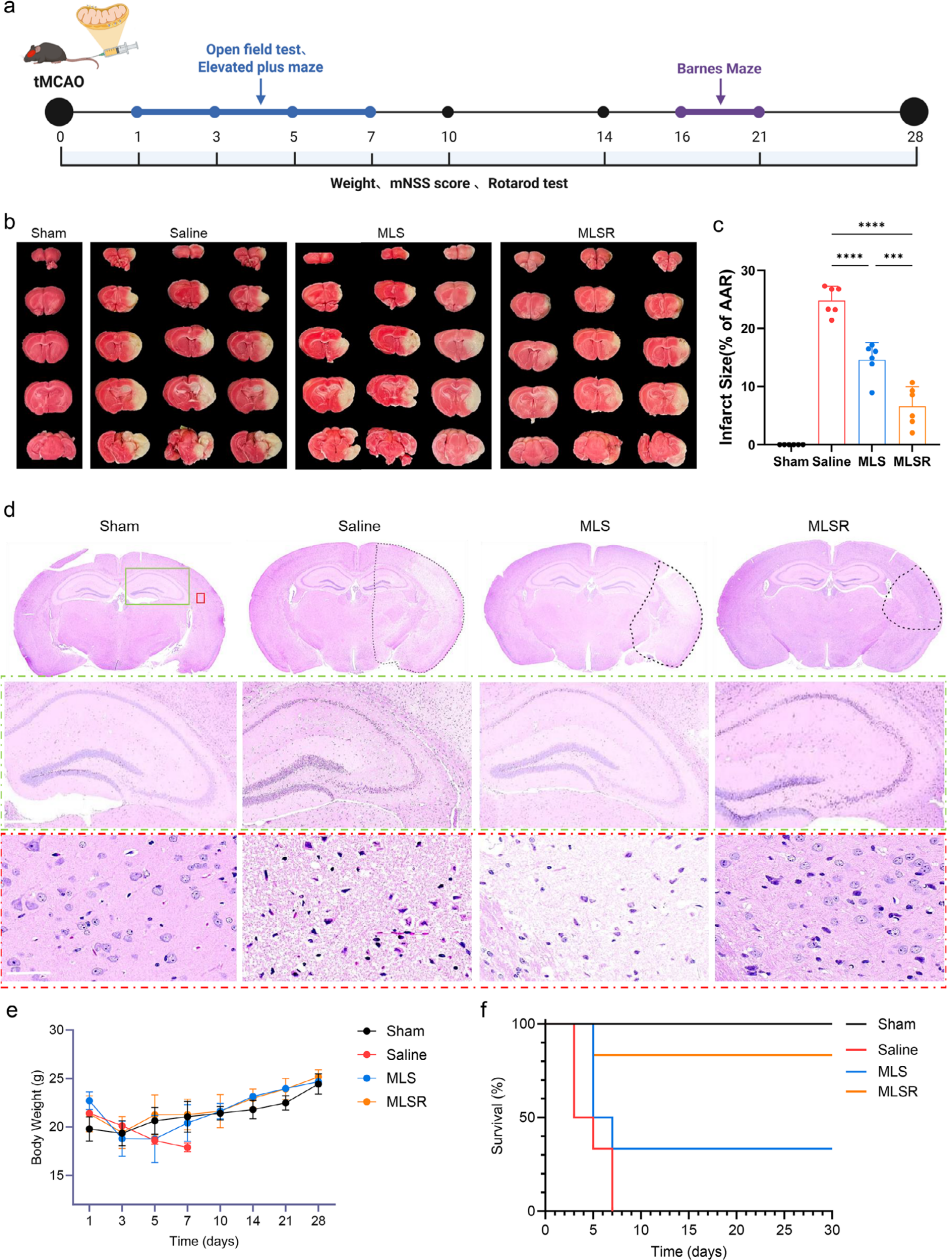
MLSR‐mediated positive autophagic flux enhanced in vivo therapeutic effects. (a) Schematic illustration of the experimental timeline for tMCAO mice. (b,c) TTC staining and quantification of cerebral infarct volume in each group. (one‐way ANOVA; n  =  6 per group). (d) H&E staining of brain slices in each group. scale bar: 500 and 50 µm. (e) Changes in body weight of tMCAO mice treated with Saline, MLS, or MLSR during the period of treatment. (f) Changes in survival rate of tMCAO mice treated with Saline, MLS, or MLSR during the period of treatment. Data are presented as mean ± s.d., and n represents biological replicates. ^*^ *p* < 0.05; ^**^ *p* < 0.01; ^***^ *p* < 0.001; ^****^ *p* < 0.0001 are considered as statistically significant.

Next, we evaluated the therapeutic efficacy of MLSR in a tMCAO mice model (Figure 7a). All tMCAO mice were randomly divided into four groups and administered a single intravenous dose of saline, MLS, or MLSR immediately after reperfusion (n  =  6 per group). According to this scheme (Figure 7a), brains were collected 24 h post‑reperfusion, and histopathological assessments revealed that MLSR rapidly conferred potent neuroprotection compared to saline and MLS treatments. Brain slices stained with 2,3,5‐triphenyltetrazolium chloride (TTC) demonstrated that tMCAO mice treated with saline showed distinctive cerebral infarct volumes, which were significantly reduced from 24.8% to 14.6% in the MLS group and to 6.6% in the MLSR group (Figure 7b,c). The remarkable neuroprotective effects in the ischemic hemisphere were validated using hematoxylin and eosin (HE) and Nissl staining. In the saline group, HE staining revealed significant neuronal loss, disorganization, and vacuolization in the hippocampus and cortex of the ischemic brain. By contrast, these features were partially attenuated by MLS treatment and were virtually absent in the MLSR‑treated brain tissue, where the cytoarchitecture resembled that of the normal brain tissue (Figure 7d). Consistent with these findings, Nissl staining demonstrated superior preservation of neuronal integrity after IR injury in MLSR‑treated brains compared to both saline and MLS groups, as evidenced by the increased Nissl body density and morphology recovery in the ischemic hemisphere (Figure S29). The major organs of mice that received different treatments were collected to assess systemic toxicity. The HE staining of tissue sections demonstrated no pathological changes in the heart, liver, spleen, lungs, or kidneys, revealing the outstanding biological safety of this mitochondrial transplantation platform (Figure S30). We also evaluated the systemic inflammation and hematological safety following MLSR administration. The results demonstrated negligible hemolysis, no significant alteration in coagulation parameters, and no elevation in systemic inflammatory cytokines compared with the PBS group (Figure S31). The impressive therapeutic benefits of mitochondrial transplantation translate into systemic outcomes. Throughout the 30‐day period, we monitored changes in survival rates and body weights. In the early post‐stroke period, saline‑treated mice exhibited significant weight loss, and all of the mice died within the first week. By contrast, both the MLS and MLSR groups showed rapid weight recovery, with MLSR‐treated mice restoring their baseline body weight by day 5 compared to the sham group (Figure 7e). Moreover, MLSR treatment conferred a significant improvement in survival rates, with 83% of the mice surviving to day 30 compared to 33% in the MLS group (Figure 7f).

### 2.8 MLSR‐Mediated Positive Autophagic Flux Reversed Neuroinflammation by Suppressing Microglia Activation

**FIGURE 8:**
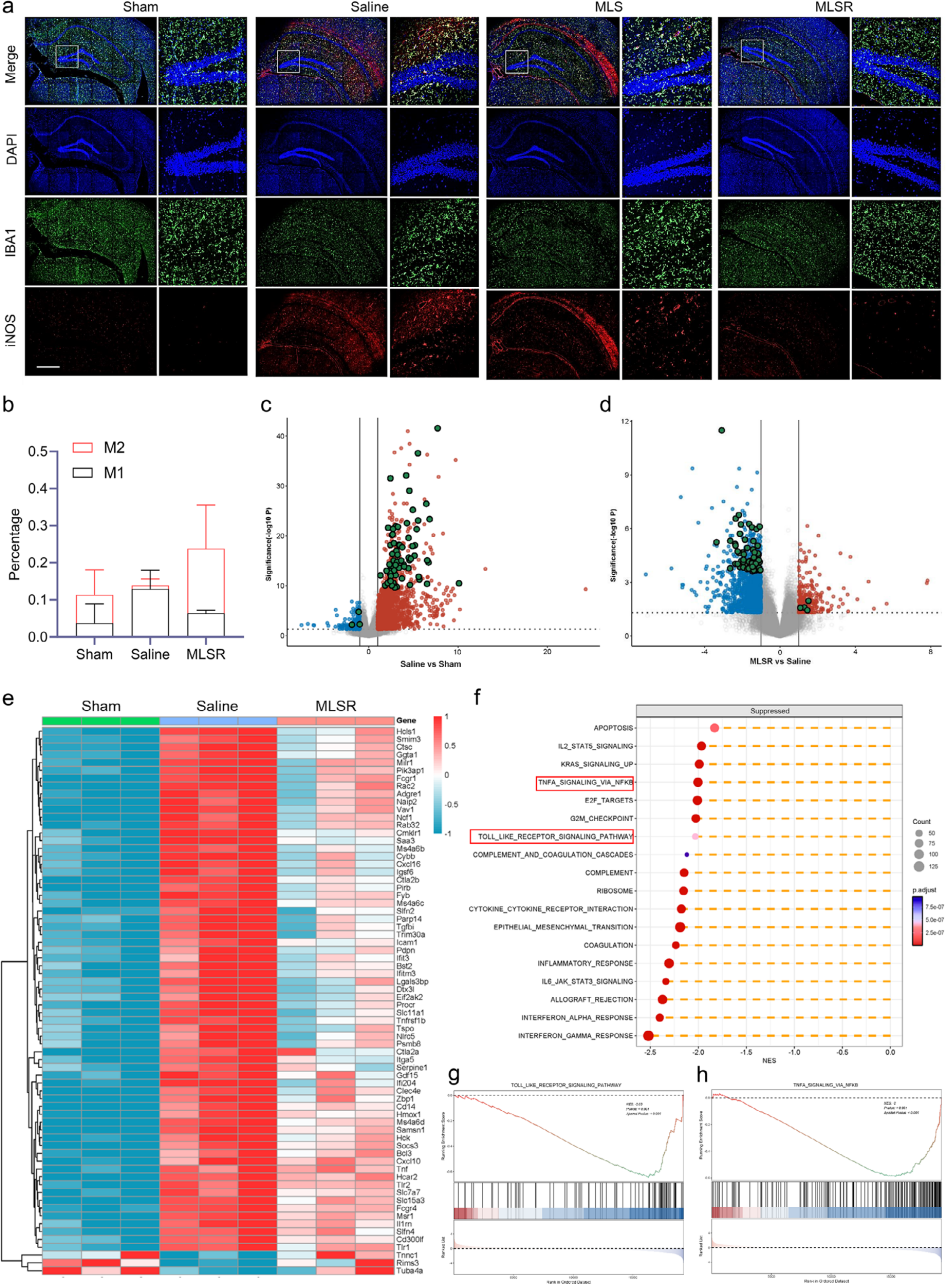
MLSR‐mediated positive autophagic flux reversed neuroinflammation by suppressing microglia activation. (a) Immunofluorescence images of Iba1^+^/iNOS^+^ microglia in ischemic brains (hippocampus) of tMCAO mice after different treatments. Scale bar: 2.5 mm. (b) Proportion of each microglia subtype in Sham, Saline, and MLSR groups. (c) Volcano plot showed the dynamic expression of genes between the Saline and Sham group. Red dots indicated upregulated genes, blue dots represented downregulated genes, while green dots were M1‐associated genes. (d) Similar result of the volcano plot showed the dynamic expression of genes between MLSR and the Saline group. (e) Heatmap showed the dynamic changes in selected gene expression between Sham, Saline, and MLSR groups. (f–h) The gene set enrichment analysis (GSEA) showed the suppressed biological processes and signaling pathways in the MLSR group.

The neuroprotective effects of MLSR in tMCAO mice were associated with its capacity to restore autophagic flux. Previous results examined the role of positive autophagic flux in reducing the secretion of undegraded mitochondria in the form of exosomes, thereby inhibiting proinflammatory crosstalk between neurons and microglia. Therefore, we examined microglial activation following MLSR treatment to evaluate its anti‐inflammatory potential in vivo. Immunofluorescence staining of ischemic brain sections revealed a marked induction of Iba1^+^/iNOS^+^ microglia in the saline group, which is indicative of M1‐type proinflammatory activation. By contrast, MLSR‐treated tMCAO mice showed a significant reduction in iNOS expression, particularly in the cortex and hippocampus, suggesting inhibition of microglial polarization toward the M1 phenotype (Figure 8a; Figure S32).

Subsequently, RNA sequencing (RNA‐seq) was conducted to gain further insight into the inhibition of microglial activation by MLSR treatment. An established computational algorithm, ImmuCC, was used to estimate the composition of immune cells from gene expression profiles (Figure S33) [30]. The saline group showed increased M1‐like microglial polarization compared to the sham‐operated controls, indicating the deterioration of brain injury after IS. Notably, the tMCAO mice treated with MLSR had a higher percentage of M2‐like microglia. The anti‐inflammatory changes at the ischemic site exerted the opposite effect, which could rescue local neuroinflammation and promote functional recovery of the brain (Figure 8b). GSE69607 is a gene expression dataset of bone marrow‐derived macrophages (BMDMs) in which differentially expressed genes between the M1 and M2 phenotypes are identified [31]. Following I/R injury, microglia from saline‐treated tMCAO mice upregulated M1‐associated genes; however, this tendency was attenuated in the MLSR group (Figure 8c–e). Gene set enrichment analysis (GSEA) identified the downregulation of pathways associated with Toll‐like receptor signaling and NF‐κB activation in response to TNF in the MLSR group, which were known to trigger early immune responses and facilitate M1 polarization (Figure 8f–h). Collectively, these results provide compelling evidence that MLSR mitigates neuroinflammation by modulating microglial phenotypes through the restoration of autophagic flux.

### 2.9 Improved Post‐Stroke Functional Recovery Following MLSR Treatment in tMCAO Mice

**FIGURE 9:**
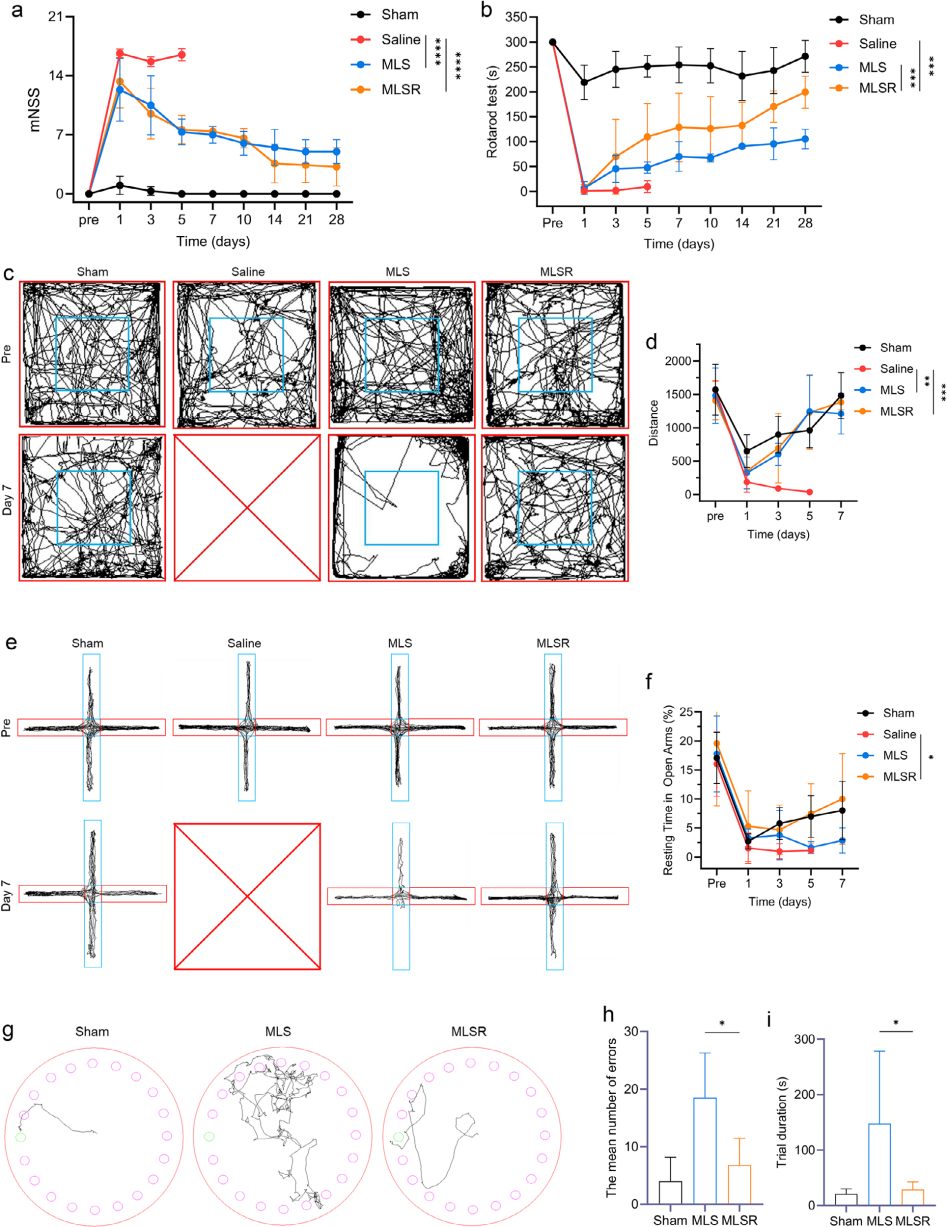
Improved post‐stroke functional recovery following MLSR treatment in tMCAO mice. (a) Mean mNSS scores for neurological deficit evaluation (two‐way ANOVA; n  =  6 per group). (b) Rotarod test results after the different treatments (two‐way ANOVA; n  =  6 per group). (c,d) Representative images of open field tests, and analysis of total distance (two‐way ANOVA; n  =  6 per group). (e,f) Representative images of elevated plus maze tests, and analysis of entries and resting time in the open arms (two‐way ANOVA; n  =  6 per group). (g–i) Representative images of the Barnes maze test, and analysis of the number of errors and time spent to find the right hole (one‐way ANOVA; n  =  6 per group). Data are presented as mean ± s.d., and n represents biological replicates. ^*^ *p* < 0.05; ^**^ *p* < 0.01; ^***^ *p* < 0.001; ^****^ *p* < 0.0001 are considered as statistically significant.

After receiving different treatments, we evaluated the long‐term functional recovery of tMCAO mice. A series of behavioral tests, including movement, anxiety, and cognition tests were performed on tMCAO mice over a 28‐day period. First, the sensorimotor function of tMCAO mice was significantly impaired after surgery, as indicated by the modified neurological severity score (mNSS, Figure 9a). As expected, MLSR‐treated mice experienced earlier and sustained improvements in neurological deficits throughout the study period. The rotarod test was used to further assess the post‐stroke impaired motor function (Figure 9b). According to these results, the retention time of the MLSR‐treated mice showed almost full recovery by day 3 post‐injury, whereas the MLS group displayed only limited improvement. Simultaneously, multiple behavioral tests, including the open field (OF) and elevated plus maze (EPM) tests were conducted because post‐stroke depression is a common outcome following IS and is associated with poor recovery. In the open field test, the total distance and time spent in the center for the saline‐treated mice were significantly lower than those of the sham group on day 2, and this decline persisted for the duration of the post‐stroke period. By contrast, MLSR treatment significantly reversed this phenomenon by day 7 (Figure 9c,d; Figure S34). Although the time spent in the center area in the MLSR group remained slightly reduced compared to that in the sham group, no significant difference was observed in the total distance traveled. Similarly, in the EPM test, the tMCAO mice exhibited a significant reduction in both the duration and number of entries into the open arms, suggesting a higher level of anxiety (Figure 9e,f; Figure S35). In comparison to the saline group, the mice treated with MLS showed similar exploring routes and heat maps, with negligible improvement. Conversely, MLSR treatment significantly enhanced exploration of the open arms and the residence time, indicating a reduction in anxiety levels and promotion of locomotor activity. The Barnes maze test was performed to investigate the long‐term learning ability and spatial memory on day 21. Compared with the MLS group, the MLSR‐treated mice made fewer errors and spent less time finding the correct hole (Figure 9g–i). Moreover, there were no significant differences in either parameter between the sham and MLSR groups, indicating successful long‐term neurofunctional recovery in tMCAO mice after MLSR treatment. These findings demonstrate that MLSR confers robust and long‐lasting benefits in a variety of behavioral domains following IS, highlighting its potential to promote comprehensive functional recovery by facilitating positive autophagic flux.

## 3 Conclusions

Mitochondrial transplantation has emerged as a prominent research topic in a wide range of acute and chronic diseases. Exogenous mitochondria show promising protective effects in damaged recipient cells, demonstrating notable improvements in restoring mitochondrial activity, increasing ATP production, and improving cell viability. However, the mechanisms underlying mitochondrial transplantation remain elusive. Meanwhile, several limitations and challenges must be carefully considered before clinical application. For instance, the source of exogenous mitochondria remains a critical issue, involving challenges in the inter‐individual variability, mitochondrial quality control, and large‐scale production. A comprehensive evaluation of long‐term biosafety and immunogenicity is also necessary because DAMPs and mtDNA may potentially trigger immune activation. Moreover, systemic delivery efficiency and off‐target accumulation represent another challenge.

Recent advances in nanotechnology, especially in surface modification strategies, have renewed the therapeutic potential of mitochondrial transplantation. Surface modification with targeting molecules facilitates precise mitochondrial delivery and internalization via non‐invasive administration routes. In this study, we demonstrated that functionalized starch is an effective coating material with several innovative features, and it permits the targeted delivery of mitochondria to the ischemic penumbra. We also investigated the mechanisms by which exogenous mitochondria trigger mitophagy. This natural process selectively degrades dysfunctional mitochondria following IR injury to maintain a stable intracellular equilibrium. However, the bursting of ROS during IR injury blocks autophagosome–lysosome fusion and arrests autophagic flux by activating the RIP1/RIP3 pathway. Using the helical structure of starch to establish a drug delivery system, we demonstrated that the co‐delivery of resveratrol with mitochondria via this starch modification strategy can restore autophagic flux. Moreover, through a series of in vitro and in vivo studies, we demonstrated that positive autophagic flux suppressed the release of damaged mitochondrial components, thereby inhibiting proinflammatory crosstalk between neurons and microglia. This promising and innovative modification strategy expands the mechanistic understanding of positive autophagic flux in mitochondrial transplantation and has important implications for renewing the therapeutic potential of mitochondrial transplantation. Despite these promising advances, further systematic preclinical and translational studies are warranted to bridge the gap between experimental success and clinical application. Although starch‐based coating significantly improved mitochondrial stability during storage and facilitated precise mitochondrial delivery via non‐invasive administration route, standardized cryopreservation protocols and Good Manufacturing Practice (GMP) compliance remain to be established. Moreover, determining the precise therapeutic window for mitochondrial transplantation during reperfusion therapy is essential for clinical translation.

## 4 Experimental Section

### 4.1 Cell Lines and Culture Conditions

HT22 cells (RRID: CVCL_0321) were obtained from Procell Life Science & Technology Co., Ltd. (Wuhan, China) in March 2024. And BV‐2 cells (RRID: CVCL_0182) were also obtained from Procell Life Science & Technology Co., Ltd. (Wuhan, China) in December 2024. HCMEC/D3 cells (RRID: CVCL_U985) were obtained from Shanghai Jinyuan Biotechnology Co., Ltd. (Shanghai, China) in May 2024. All cell lines were verified by short tandem repeat (STR) profiling, and the STR report was provided by the original supplier. The cells were cultured in DMEM supplemented with 10% fetal bovine serum (FBS), and further supplemented with penicillin (100 U/mL) and streptomycin (100 U/mL), maintained in a humidified incubator at 37 °C with 5% CO_2_.

### 4.2 Animals

The Sprague‐Dawley (SD) rats (male, 6–8 weeks, 200–320 g) and C57BL/6 mice (male, 6–8 weeks, 20–22 g) were purchased from the Experimental Animal Center of Xi'an Jiaotong University. All the animal experiments were performed in accordance with the ethical guidelines (Health Science Center of XJTU Approval for Research Involving Animals No.2019066), which was approved by the Biomedical Ethics Committee of Health Science Center of Xi'an Jiaotong University.

### 4.3 Z‑Starch Synthesis

Starch (1.62 g) was dissolved in 30 mL of deionized water, and 5 mL of 20% NaOH was added dropwise to the solution for alkalization. Then, 0.31 g of 3‐((3‐chloropropyl) dimethylammonio) propane‐1‐sulfonate was added to the reaction, and stirred for 6 h at 60 °C. After the reaction, the solution was adjusted to a neutral pH and dialyzed against deionized water. Finally, the solution was concentrated by rotary evaporation and slowly added dropwise into ethanol to obtain the precipitate.

### 4.4 Z‐Starch‐TPP Synthesis

Z‑Starch (1.62 g), 4‐carboxybutyl triphenylphosphonium bromide (0.52 g), 1‐ethyl‐3‐(3‐dimethylaminopropyl) carbodiimide (EDC, 0.36 g), and 4‐dimethylaminopyridine (DMAP, 0.22 g) were dissolved in 10 mL of DMSO, and stirred for 72 h at room temperature. After the reaction, the solution was dialyzed against deionized water, and the product was obtained by lyophilization.

### 4.5 The Loading of Resveratrol and In Vitro Release Profiles

The resveratrol was encapsulated in the helical cavity of starch through hydrophobic interactions. Specifically, 250 µL of the resveratrol solution (10 mg mL^−1^) was slowly added dropwise to the solution of Z‐Starch‐TPP (10 mL, 1 mg mL^−1^), followed by stirring for 6 h to ensure most of the resveratrol was loaded. After dialysis (MWCO, 3.5 kDa) for 2 h against distilled water, the actual concentration of loaded resveratrol was calculated by measuring the absorbance at 306 nm. To evaluate the release profiles under different conditions, the solution of SR was concentrated and re‐diluted in pH 7.4 and pH 5.5 buffers. Taking samples from the solution at 0, 1, 2, 4, 8, 12, and 24 h to test the decrease in absorbance.

### 4.6 Isolation and Quantitative Analysis of Platelet Mitochondria

Platelets were isolated from SD rats using a platelet separation kit (Slb2011M, Solarbio), and mitochondria were isolated from the platelets using a mitochondrial isolation kit (C3601, Solarbio). Subsequently, the solution of isolated mitochondria was diluted with PBS to a final volume of 1 mL, and the mitochondrial concentration was quantified via flow cytometry.

### 4.7 Detection of Isolated Mitochondrial Purity

The high purity of isolated exogenous mitochondria was confirmed through the analysis of protein expression profiles. Platelets, isolated mitochondria, and MSR were lysed with mitochondrial lysis buffer (C3601, Beyotime), and the supernatant was collected after centrifugation at 12 000 × g for 20 min. Equal amounts of proteins were separated by SDS‐PAGE electrophoresis, followed by transfer to a polyvinylidene difluoride (PVDF) or nitrocellulose membrane. After blocking, the membrane was incubated with primary antibodies against PINK1 (1:1000), GAPDH (1:1000), and TOMM20 (1:1000) overnight at 4 °C, and the corresponding horseradish peroxidase‐labeled secondary antibody (1:5000) was incubated at room temperature for 1.5 h. The Bio‐Rad imaging system was used to visualize specific bands, and ImageJ software was used to analyze the obtained images.

### 4.8 Molecular Dynamics (MD) Simulation

All simulations were performed using the molecular dynamics (MD) simulation software GROMACS 2022 [32]. The force field of phospholipid molecules adopted the CHARMM36m force field [33]. And resveratrol molecules were assigned the CgenFF force field [34]. Force field parameter files for all molecules were obtained from the online platform CHARMM‐GUI [35]. All simulations were carried out under normal temperature and pressure (303.15 K, 1 standard atmosphere).

### 4.9 MSR Preparation and Co‐Localization Analysis

SR was prepared by adding the resveratrol solution (200 µL, 1 mg mL^−1^) in the solution of Z‐Starch‐TPP (10 mL, 1 mg mL^−1^) and stirring for 6 h. Then the mixed solution was dialysed against PBS to remove free resveratrol. The isolated mitochondria (50 µL, 2.29 × 10^10^ mL^−1^) were directly incubated with SR (1 mL) to obtain surface‐modified mitochondria (MSR). After incubation for 20 min at 4 °C on a shaker, the solution was centrifuged at 3000 g for 10 min to collect the MSR. Moreover, the isolated mitochondria were stained with 200 nm MitoTracker Red CMXRos (Ex/Em = 579/599 nm) on ice for 10 min. Then, the C6‐labeled SR (Ex/Em = 450/505 nm) was added to the solution of isolated mitochondria, and incubated under shaking conditions at 4 °C for 20 min. The successful construction of MSR was observed under a laser confocal microscope.

### 4.10 Morphology Characterization

The obtained mitochondria and MSR precipitates were mixed with the electron microscopy fixative and fixed at room temperature for 30 min. Subsequently, the samples were rinsed with 0.1 m phosphate buffer solution (PBS) and ultrapure water, followed by fixation with 1% osmium tetroxide. This was followed by further rinsing with ultrapure water and gradient dehydration with ethanol. Finally, the precipitates were infiltrated with 100% embedding medium, and the resulting ultrathin sections were stained with 2% uranyl acetate and lead citrate before observation under a transmission electron microscope.

### 4.11 Long‐term Preservation Conditions For Isolated Mitochondria

The obtained free mitochondria and MSR were prepared into a homogeneous suspension with pre‐cooled mitochondrial storage solution (C3609, Beyotime), and stored at 4 °C for subsequent experiments.

### 4.12 The Structural Stability of MSR

Equal amounts of free mitochondria and MSR were incubated with trypsin (10%, v/v) at 37 °C. Subsequently, the precipitates were collected from mixed solutions at different time points by centrifugation at 11 000 × g for extracting mitochondria proteins. The concentrations of total protein were determined using the BCA Protein Concentration Rapid Assay Kit (P0398, Beyotime). Correspondingly, 50 µL of the mitochondrial protein supernatant from each timepoint was used for the quantitative detection of key mitochondrial proteins, including PINK1 and COX IV.

### 4.13 Mitochondrial Transplantation and Cellular Uptake Assessment

According to the quantitative analysis, the number of MSR used for transplantation corresponded to a 3:1 ratio (mitochondria: recipient cells), and this ratio was also used in subsequent in vitro experiments. HT22 cells were seeded in six‐well plates until the cell density reached 80% (approximately 2.4 × 10^6^ cells). Then, MitoTracker‐labeled MSR were incubated with HT22 cells, and samples were collected at different time intervals (0, 0.25, 0.5, 1 h). Intracellular fluorescence was measured by a flow cytometer to evaluate the cellular internalization of MSR.

### 4.14 Oxygen‐Glucose Deprivation/Reperfusion (OGD/R) Model

Cells were cultured in glucose‐free DMEM in a hypoxic chamber (95% N_2_ + 5% CO_2_) for 4 h to simulate the ischemic condition. Then, the culture medium was changed to complete medium and incubated in a humidified cell incubator (37 °C, 5% CO_2_) during the reperfusion phase. For the treatment groups, mitochondrial transplantation was performed in the reperfusion phase at the previously specified ratio.

### 4.15 The Translocation of Parkin

To assess the translocation of Parkin to endogenous mitochondria in recipient cells after mitochondrial transplantation, cells from different groups were collected after 1 h of reperfusion. Then, mitochondria proteins were extracted, and western blot analysis was performed as previously described, using the following primary antibodies: Parkin (1:1000) and TOMM20 (1:1000) to label the target proteins.

### 4.16 Detection of Mitophagy‐Related Proteins

Cells from different groups were collected after 6 h of reperfusion, and lysed by shaking on ice with RIPA lysis buffer (containing phosphatase and protease inhibitors) to ensure complete lysis. Western blot analysis was performed as previously described, using the following primary antibodies: P62 (1:1000), LC3B (1:1000), and β‐actin (1:1000) to label the target proteins.

### 4.17 Autophagic Flux Assay

The mRFP‐GFP‐LC3 lentivirus was added to cells (3 MOI) for transfection. After incubation for 48 h, OGD/R was established, and the cells were treated with MS or MSR. Then, the fluorescence signals of mRFP and GFP were detected at 587/610 nm and 488/507 nm using a CLSM.

### 4.18 Co‐Localization Analysis of Exogenous Mitochondria and Lysosomes

The positively charged Au nanoparticles (Nano lab‐Au5, Nanjing Mice Technology Co., Ltd.) were used to label isolated mitochondria by incubating them with the solution of exogenous mitochondria at 4 °C for 20 min with shaking. Then, Au nanoparticles labeled mitochondria were collected by centrifugation at 3000 g for 10 min at 4 °C, and then added to HT22 cells after OGD. The cell sample was collected after 1 h of incubation for TEM imaging.

### 4.19 Detection of RIP1/RIP3 Pathway‐Related Proteins by Western Blot

The cells from different groups were collected after 6 h of reperfusion, and lysed thoroughly by shaking in RIPA lysis buffer (containing phosphatase inhibitors and protease inhibitors) on ice. Western blot analysis was performed as previously described, using the following antibodies: RIP1, P‐RIP3, and RIP3 to label the target proteins.

### 4.20 Detection of mtDNA Release

The released mtDNA in supernatants from each group were extracted strictly following the protocol of the Cell Genomic DNA Extraction Kit (DP304, TIANGEN). Meanwhile, total RNA was extracted using Super FastPure Cell RNA Isolation Kit (RC102‐01, Vazyme), and complementary DNA (cDNA) was synthesized using the Evo M‐MLV RT Mix Kit (AG11728, ACCURATE BIOTECHNOLOGY(HUNAN)CO., LTD, ChangSha, China) according to the manufacturer's instructions. The cDNA was amplified using SYBR (AG11701, ACCURATE BIOTECHNOLOGY(HUNAN)CO., LTD, ChangSha, China). The sequences of the primers used are as follows:

COX1 forward primer: CAAATGAAAGCGGCTGGATAAC;

COX1 reverse primer: AGCCTAGATAGCCCAGATACTC;

ND1 forward primer: TCCGAGCATCTTATCCACGC;

ND1 reverse primer: GTATGGTGGTACTCCCGCTG;

β‐actin forward primer: GTGACGTTGACATCCGTAAAGA;

β‐actin reverse primer: GCCGGACTCATCGTACTCC.

### 4.21 Analysis of Macrophage Polarization

In the transwell model, HT22 cells were seeded into the upper chamber (pore size 0.4 µm, Corning) at a density of 3 × 10^4^ per well. Simultaneously, BV2 cells were seeded into the lower chamber at a density of 5 × 10^4^ per well. M2‐like microglia were obtained by stimulation with 20 ng mL^−1^ IL‐4 (HY‐P7080, MCE) for 24 h. Then, HT22 cells underwent the OGD/R, and different treatments were administered to HT22 cells in the upper chamber. Fluorescence imaging was performed to investigate the microglial phenotype transition in different groups by immunofluorescence staining with antibodies against CD16/32 and CD206 after co‐incubation for 24 h.

### 4.22 Association Between the Restoration of Autophagic Flux and the RIP1/RIP3‐Exosome Axis

Necrostatin‐1 (Nec‐1, RIP1 inhibitor) and Necrosulfonamide (NSA, MLKL inhibitor) were further introduced as mechanistic controls. HT22 cells were seeded in six‐well plates and subjected to OGD for 4 h. Then, the medium was replaced with complete medium, and the cells were treated with PBS, MSR (1.5 × 10^5^), Nec‐1 (30 µm), and NSA (1 µm), respectively. The proteins related to autophagic flux and necroptosis were collected at the same timepoint for Western blot analysis. Moreover, MS + Nec‐1 (1.5 × 10^5^, 30 µm) and MS + NSA (1.5 × 10^5^, 1 µm) were used for the combination groups.

### 4.23 Lf‐Z‐Starch‐TPP Synthesis

Z‐Starch‐TPP (2.0 g), NaOH (2.4 g), and chloroacetic acid (0.2 g) were dissolved in 20 mL of deionized water, and were reacted for 4 h at 60 °C. After the reaction, the pH of the solution was adjusted to 7.5–8.5. Then, EDC (100 mg), N‐hydroxysuccinimide (NHS, 20 mg), and Lf (20 mg) were added, and the mixture was reacted for 48 h at room temperature. The solution was dialyzed against deionized water overnight, and the Lf‐modified Z‐Starch‐TPP was obtained after lyophilization.

### 4.24 In Vitro BBB Model

The upper chamber of a Transwell culture plate (pore size 0.4 µm, Corning) was coated with matrix gel (GelNestTM) for 1 h and then seeded with hCMEC/D3 cells, while HT22 cells were seeded in the lower chamber (placed with coverslips coated with poly‐L‐lysine hydrobromide). When the trans‐endothelial electrical resistance (TEER) value reached above 200 Ω cm^2^, the upper chamber could be used for subsequent experiments. The upper chamber was treated with C6‐labeled MSR or MLSR, and incubated for 2 and 6 h, respectively; the fluorescence intensity in the lower chamber was then observed using CLSM.

### 4.25 Biodistribution

To investigate the in vivo distribution, mice were intravenously injected with 150 µL of Ce6‐labeled MSR or MLSR (5 mg kg^−1^ based on Ce6). Fluorescence imaging of the brain was performed using IVIS image system at fixed time intervals (0, 5, 15, 30 min, 1, 2, 3 h, n = 3). Moreover, the major organs (heart, liver, spleen, lungs, kidney, and brain) were harvested for ex vivo fluorescence imaging at 24 h post‐injection. Fluorescence intensities were quantified using Living Image software. Moreover, exogenous mitochondria were incubated with MitoTracker (MitoBright LT Deep Red, DOJINDO, MT12, Ex: 640 nm/Em: 650–700 nm) with slow shaking at 37 °C for 30 mins. Subsequently, the precipitate was collected through centrifugation at 11 000 × g and thoroughly washed with pre‐chilled PBS. The IVIS imaging system were also used to perform fluorescence imaging of the brain at fixed time points.

### 4.26 Establishment of tMCAO Model

Anesthesia was induced by intraperitoneal injection of 1% sodium pentobarbital (40 mg kg^−1^), ensuring that the mice experienced no pain during the surgery or experiment. The mice were placed in a supine position on the surgical table, and the neck and head were exposed. The common carotid artery and left external carotid artery were exposed via a middle incision of the neck. A 0.2‐mm silicon‐coated filament was inserted into the internal carotid artery and advanced approximately 8–10 mm to occlude the middle cerebral artery (MCA). After 50 min of occlusion, the filament was removed to restore MCA blood flow. Sham‐operated mice received the same surgical procedure, but no filament was inserted.

### 4.27 Laser Speckle Blood Flux Imaging

Cerebral blood flux (CBF) in the cortical region was monitored using a Laser Speckle Contrast Imaging system (RFLSI ZW, RWD, ShenZhen). After isoflurane inhalation anesthesia, the scalps of the mice were shaved, and the scalps were disinfected with 1% iodine solution. Then, a midline incision was made along the top of the skull to expose the entire skull, and the position was adjusted so that the laser speckle was located 2 mm posterior to the anterior fontanelle and 2 mm lateral to the midline, while keeping the skull moist and recording the changes in cerebral blood flux. CBF was recorded immediately after the procedure and following reperfusion to verify the success of the model.

### 4.28 Therapeutic Effects of MLSR in tMCAO Mice

All mice in which the tMCAO model was successfully established were assigned to four groups (n = 6 per group), including the Sham, Saline, MLS, and MLSR groups. After removing the filament for reperfusion, MLS and MLSR were intravenously injected once at a concentration of 4 × 10^8^ mitochondria per mice. Mice in the Sham group were intravenously injected with PBS.

### 4.29 TTC Staining

The brains of mice from each group were harvested after 24 h of reperfusion, and the brain tissue was sectioned vertically on the coronal plane (5 slices, each 2 mm thick). Subsequently, the coronal brain slices were incubated in 2% 2,3,5‐triphenyltetrazolium chloride (TTC) solution for 30 min. These sections were then photographed, and the infarct lesion area (unstained areas in each section) was calculated by Image J analysis software according to the following equation: infarct volume/total volume of non‐infarcted hemisphere.

### 4.30 Immunofluorescence Staining

After 72 h of reperfusion, all mice were anesthetized with an intraperitoneal injection of 1% sodium pentobarbital (40 mg kg^−1^), and perfused with PBS, followed by 4% paraformaldehyde. After that, the brains were fixed in 4% paraformaldehyde for 24 h, followed by dehydration treatment with 30% sucrose. Then, the dehydrated brain was subjected to paraffin embedding, sectioning, and dewaxing. The expression of microglial marker IBA1 and the M1‐type microglial marker iNOS in the hippocampus and cortical regions were stained using the Coral TSA staining kit (FRT000150T, Vive Biotechnology Shanghai Ltd.) and observed by CLSM.

### 4.31 RNA Sequencing

Brains from different groups were collected at 72 h after reperfusion. The bulk sequencing profiles were preprocessed using R programming (version 4.2.0). The DESeq2 package was utilized to screen for the differentially expressed genes (DEGs) in these datasets. The proportion of immune cells in the brain was estimated from the normalized count data of bulk RNA sequencing using a pre‐established reference signature matrix encompassing 25 immune cell types. And GSE69607 was used to identify the differentially expressed genes of M1 macrophage. The expression difference of individual gene was identified by log2(Fold change) and adjusted *p* value, in which log2FC > 1 with an adjusted *p* value < 0.05 were identified as up‐regulated genes. The gene set enrichment analysis (GSEA) based on the clusterProfile R package was used to understand the biological processes and signaling pathways in the MLSR group, in which hallmark gene sets were adopted.

### 4.32 Modified Neurological Severity (mNSS) Scores

According to the previously reported method, the mNSS reflects the changes in the neurological functions related to motor, sensory, reflex, and balance [36]. The mice from each group were assessed at days 0, 1, 3, 5, 7, 10, 14, 21, and 28 after tMCAO. The normal score for mice is 0, and a higher score indicates more severe neurological injury.

### 4.33 Behavioral Tests

All behavior tests were conducted according to the scheme.

#### 4.33.1 Rotarod Test

Mice in each group were trained three times before tMCAO modeling. The rotating rod instrument was started at a speed of 4 r/min and gradually increased to 40 r/min, ensuring that the mice could adapt to exercise on the rotating rod and stay on it for approximately 300 s. After the establishment of the tMCAO model, the test was conducted using the same protocol, and the residence time of the mice on the rotarod was recorded.

#### 4.33.2 Open Field Test

Using an open‐field box (50 × 50 × 40 cm), the mice from each group were placed in the center of the box for a 10 min of free exploration. Smart3R software (Chengdu Taimeng Software Co., Ltd., Sichuan, China) was used to record and analyze the locomotion data, including distance traveled and time spent in different regions. After each trial, the box was cleaned to avoid cross‐contamination from behavioral cues.

#### 4.33.3 Elevated Plus Maze

Mice from each group were placed in the center of the elevated maze, which consists of two open arms and two closed arms arranged in a cross shape. Smart3R software was used to record the behavior of the mice in the maze, including the number of entries and time spent in the open arms.

#### 4.33.4 Barnes Maze Test

Mice were placed on the circular turntable of the Barnes maze and exposed to bright light above the turntable. Mice were trained on the 16th day post‐surgery for five consecutive days, three times a day. On the 21st day, mice were placed back in the center of the turntable and restrained with a transparent cylinder to limit their movement. Then, the behaviors of mice were recorded after removing the cylinder, including the time taken for each mouse to reach the dark target box and the number of errors made when locating the dark box.

### 4.34 Statistical Analyses

Independent two‐sample t‐tests were used to compare two groups, while one‐way analysis of variance (ANOVA) was used for comparisons between multiple groups. All statistical analyses were performed using GraphPad Prism 8 (GraphPad Software, USA), and the graphs were plotted. The results were presented as mean ± s.d., and n is biological replicates. ^*^ *p* < 0.05; ^**^ *p* < 0.01; ^***^ *p* < 0.001; ^****^ *p* < 0.0001 are considered statistically significant.

## Conflicts of Interest

The authors declare no conflicts of interest.

## Supporting information




**Supporting File**: advs74577‐sup‐0001‐SuppMat.docx.

## Data Availability

The data that support the findings of this study are available from the corresponding author upon reasonable request.
