# Supplementary material for: Nature‐Inspired Surface Modification Strategy Reverses the Autophagic Flux Impairment of Mitochondrial Transplantation for Attenuating Ischemic Strokes
Source: Adv Sci (Weinh). 2026 Feb 25;13(26):e18969. doi: 10.1002/advs.202518969 (PMC13159099; doi:10.1002/advs.202518969)
Supplement: Supplementary file 1 — Supporting File: advs74577‐sup‐0001‐SuppMat.docx. [file ADVS-13-e18969-s001.docx]

Supporting Information

**Nature-inspired surface modification strategy reverses the autophagic flux impairment of mitochondrial transplantation for attenuating ischemic strokes**

*Nisha Wang, Qiyang Ding, Lei Shi, Ji Xia, Shuyue Zhang, Chenxin Jian, Yixiao Yan, Xiuhua Luo, Jiarui Wang, Ming Cheng, Yiqiong Jia, Hao Tian*, Wei Gao**

**1. Materials and Methods**

**1.1 Materials**

Supplementary Table 1. Chemical reagents and functional kits

| Reagent | Cat # | Source |
| --- | --- | --- |
| Corn Starch | 9005-25-8 | Sinopharm Chemical Reagent |
| N,N-Dimethylaminopropyl Chloride Hydrochloride (CDMAP•HCl) | 5407-04-5 | Aladdin Biochemical Technology |
| 4-Carboxybutyl Triphenylphosphonium Bromide | 17814-85-6 | Aladdin |
| 1-(3-Dimethylaminopropyl)-3-ethylcarbodiimide Hydrochloride (EDC) | 7084-11-9 | Aladdin |
| IR780 Iodide | I302749 | Aladdin |
| Chloroacetic acid | 79-11-8 | Aladdin |
| Resveratrol | 501-36-0 | Aladdin |
| BCA Protein Assay Kit | P0011 | Beyotime Biotechnology |
| Lyso-Tracker Red | C1046 | Beyotime Biotechnology |
| MitoTracker Red | M7512 | Thermo Fisher |
| Cell Mitochondria Isolation Kit | C3601 | Beyotime Biotechnology |
| Mitochondrial Membrane Potential Assay Kit (JC-1) | C2002S | Beyotime Biotechnology |
| ATP Detection Kit | S0026 | Beyotime Biotechnology |
| Lactoferrin (Lf) | L-047 | Sigma-Aldrich |
| 1,3-Propane Sulfonic Acid Lactone | 291250 | Sigma-Aldrich |
| Chlorin e6 (Ce6) | 425311 | Sigma-Aldrich |
| 4-Dimethylaminopyridine (DMAP) | Y0001099 | Sigma-Aldrich |
| N-Hydroxysuccinimide (NHS) | 6066-82-6 | Sigma-Aldrich |
| 2,3,5-Triphenyltetrazolium Chloride (TTC) | T8877 | Sigma-Aldrich |
| Poly-L-lysine Hydrobromide | 25988-63-0 | Sigma-Aldrich |
| Autophagy Dual-Labeled Lentivirus mRFP-GFP-LC3 | HB-LP210 0001 | Hanbio |
| Coumarin 6 (C6) | HY-N7131 | MedChemExpress |
| H2DCFDA | HY-D0940 | MedChemExpress |
| Cryostat Section Embedding Medium | 3801481 | Leica |
| Coral TSA Staining Kit | FRT000150T | Vive Biotechnology Shanghai Ltd |
| 4% Universal Tissue Fixative | AP0381 | AccuRef Scientific |
| SDS-PAGE Gel Preparation Kit | AP0641 | AccuRef Scientific |
| Isoflurane | 26675-46-7 | Rwd Life Science |
| MCAO Model Suture for Mice | MSMC21B10OPK50 | Rwd Life Science |
| Animal Peripheral Blood Platelet Isolation Kit | Slb2011M | Solarbio Science & Technology |
| Annexin V-FITC/PI Apoptosis Detection Kit | E-CK-A212 | Elabscience Biotechnology |
| Cell Viability Assay Kit (CCK-8) | E-CK-A362 | Elabscience Biotechnology |
| Au Nanoparticles | Nano lab-Au5 | Nanjing Mice Technology Co., Ltd. |
| Rabbit anti-iNOS | AB178945 | Abcam |
| Rabbit anti-IBA1 | AB178846 | Abcam |
| Rabbit anti-PINK-1 | DF7742 | Abcam |
| Rabbit anti-Parkin | AF0235 | Affinity Biosciences |
| Rabbit anti-TOM20 | AF5206 | Affinity Biosciences |
| Mouse anti-β-actin | 66009-1-Ig | Affinity Biosciences |
| Rabbit anti-P62 | 18420-1-AP | Proteintech |
| Goat anti-Rabbit (H+L) | E-AB-1003 | Proteintech |
| Goat anti-Mouse (H+L) | E-AB-1001 | Proteintech |
| CoraLite® Plus 488 Anti-Mouse CD16/32 | CL488-65080 | Proteintech |
| GAPDH | 60004-1-Ig | Proteintech |
| Rabbit anti-RIP3 | 17563-1-AP | Proteintech |
| Rabbit anti-LC3B | A5618 | ABclonal |
| Rabbit anti-RIP | 3493 | Cell Signaling Technology |
| Rabbit anti-Phospho-RIP3 | 93654 | Cell Signaling Technology |
| IL-4 | HY-P7080 | MCE |
| Rabbit anti-P-MLKL | AF7420 | Affinity Biosciences |
| Mouse anti-MLKL | 66675-1-Ig | Proteintech |
| ATP Chemiluminescence Assay Kit | E-BC-F002 | Beyotime Biotechnology |
| TIANamp Genomic DNA Kit | DP304-02 | TIANGEN Biotechnology |
| PT Test Kit | TC0307 | LEAGENE Biotechnology |
| APTT Test Kit | TC0306 | LEAGENE Biotechnology |
| Mitochondrial Respiratory Chain Complex I / NADH-CoQ Reductase Activity Assay Kit | BC0510 | Beyotime Biotechnology |
| Mouse TNF-α ELISA Kit | HJ207 | Epizyme Biotechnology |
| Mouse IL-6 ELISA Kit | HJ182 | Epizyme Biotechnology |
| Mouse IL-1β ELISA Kit | HJ177 | Epizyme Biotechnology |
| CD86 (B7-2) Monoclonal Antibody | 11-0862-82 | ThermoFisher Scientific |
| COXIV Polyclonal antibody | 11242-1-AP | Proteintech |

**1.2 Material characterization.**

^1^H NMR spectra were taken on a Nuclear Magnetic Resonance Spectrometer AVANCE NEO (Bruker) using deuterated dimethylsulfoxide (DMSO-d6) as solvents. UV-Vis absorption spectra were acquired on a PE Lambda 950 (PerkinElmer). Dynamic light scattering (DLS) was conducted on a Zetasizer Nano ZS (Malvern). TEM images were determined by Talos L120C G2. Flow cytometry analyses were performed by BD CantoⅡ (BD). Confocal laser scanning microscopy (CLSM) images were acquired using a FV4000 microscope (Olympus). Cerebral blood flux (CBF) in the cortical region was monitored using a Laser Speckle Contrast Imaging RFLSI ZW (RWD).

**2. Supporting Figures**

Figure S1. The synthesis route of Z-Starch-TPP.


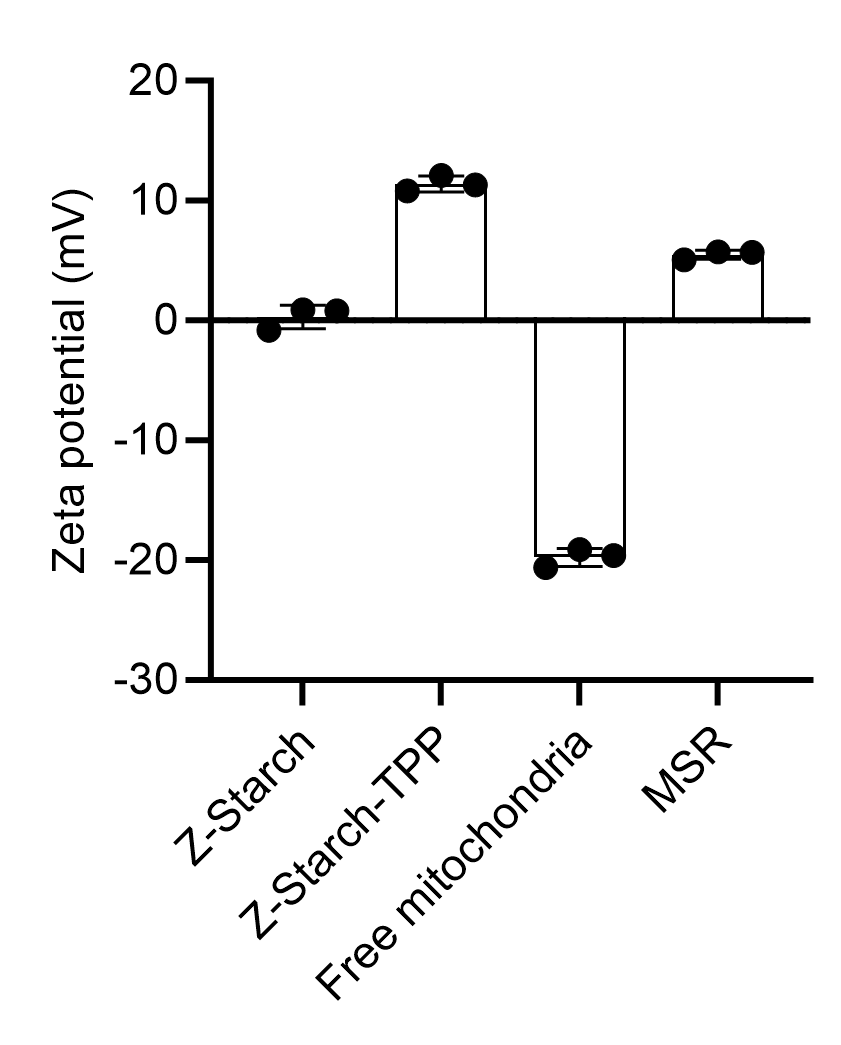


Figure S2. Zeta potential analysis of Z-Starch, Z-Starch-TPP, free mitochondria, and MSR.


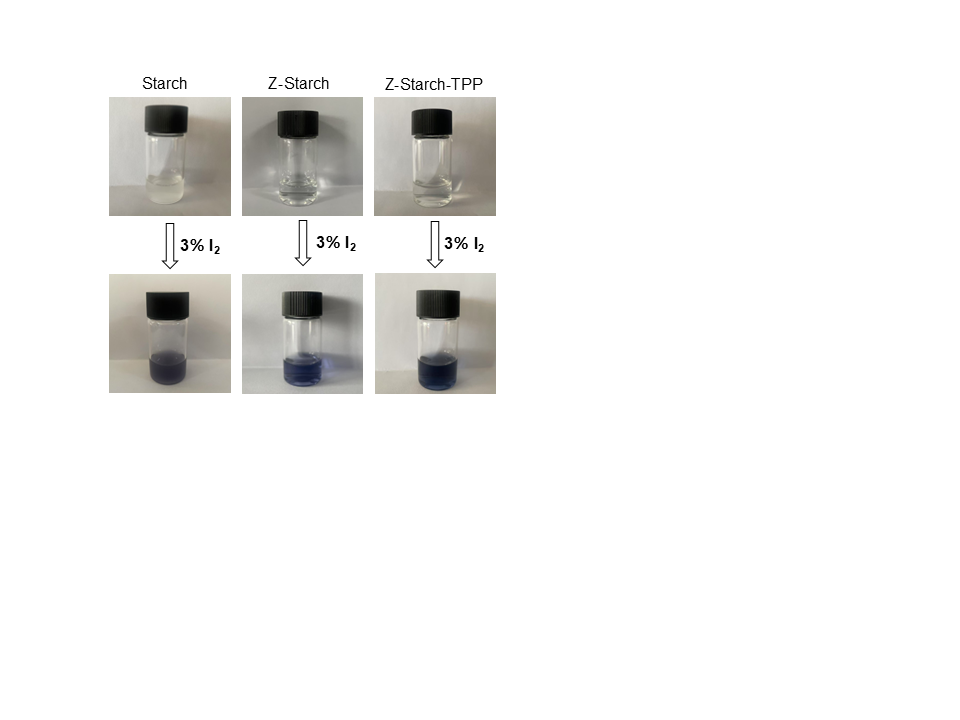


Figure S3. The iodine complexation solution of Starch, Z-Starch and Z-Starch-TPP.


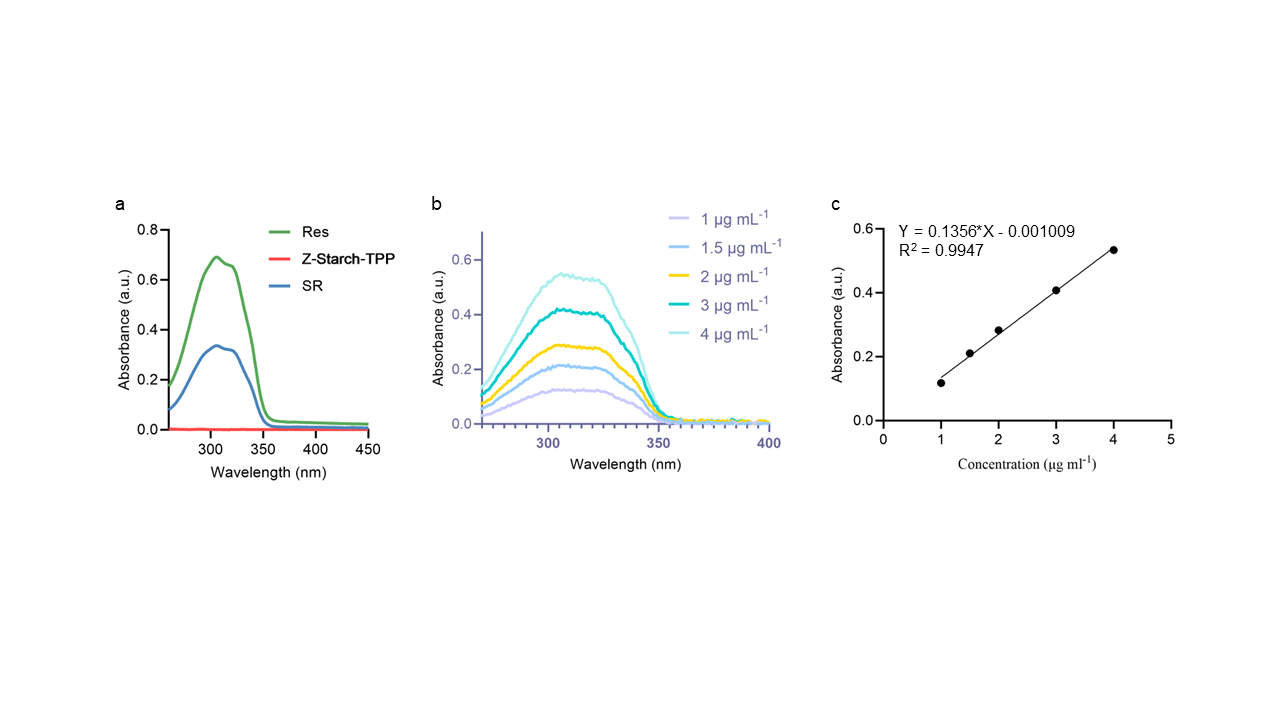


Figure S4. a) The UV-vis absorbance spectrum of Z‑Starch-TPP, resveratrol and SR. b, c) The absorbance spectra of resveratrol and its standard curve at 310 nm.


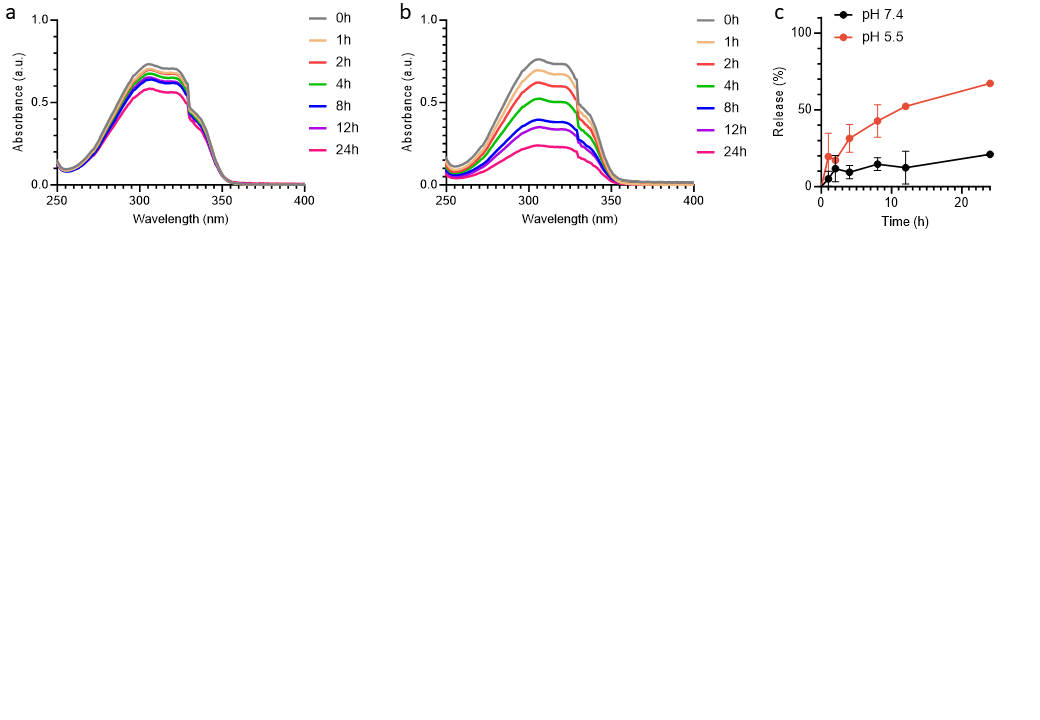


Figure S5. a) The The UV–vis spectra of resveratrol under physiological (pH 7.4) condition at different time points. b) The The UV–vis spectra of resveratrol under lysosomal-mimicking (pH 5.5) condition at different time points. c) The comparative release curves of resveratrol at different pH conditions within 24 hours (n = 3 per group).


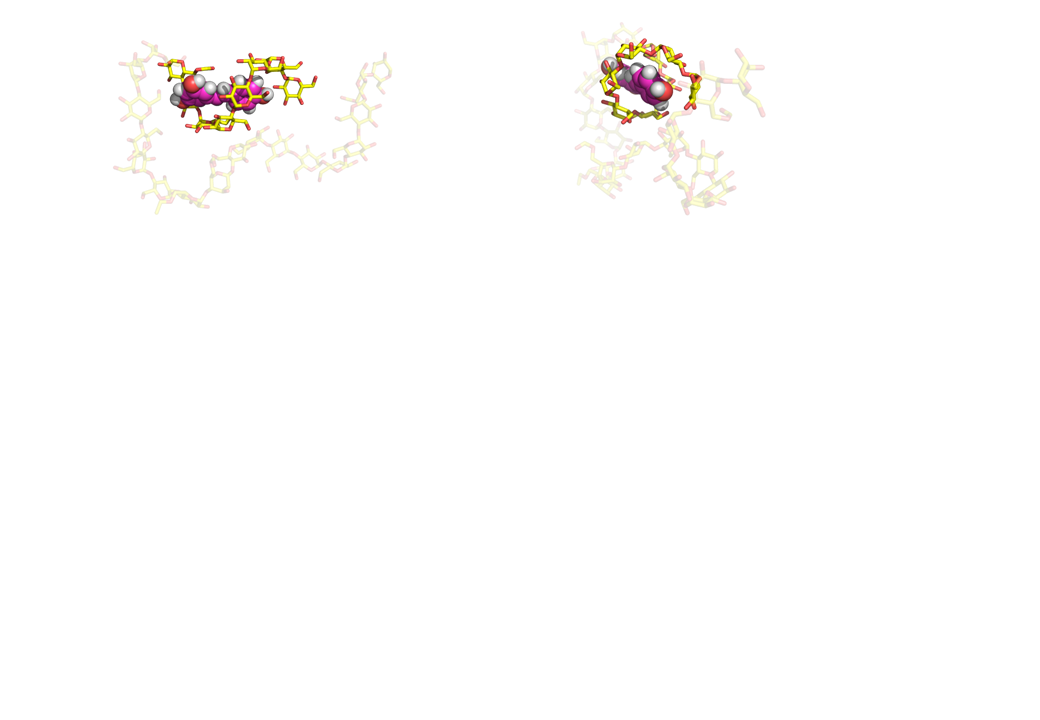


Figure S6. Snapshot of the cross section and longitudinal section of the SR to exhibit successful encapsulation of resveratrol in the helical cavity.


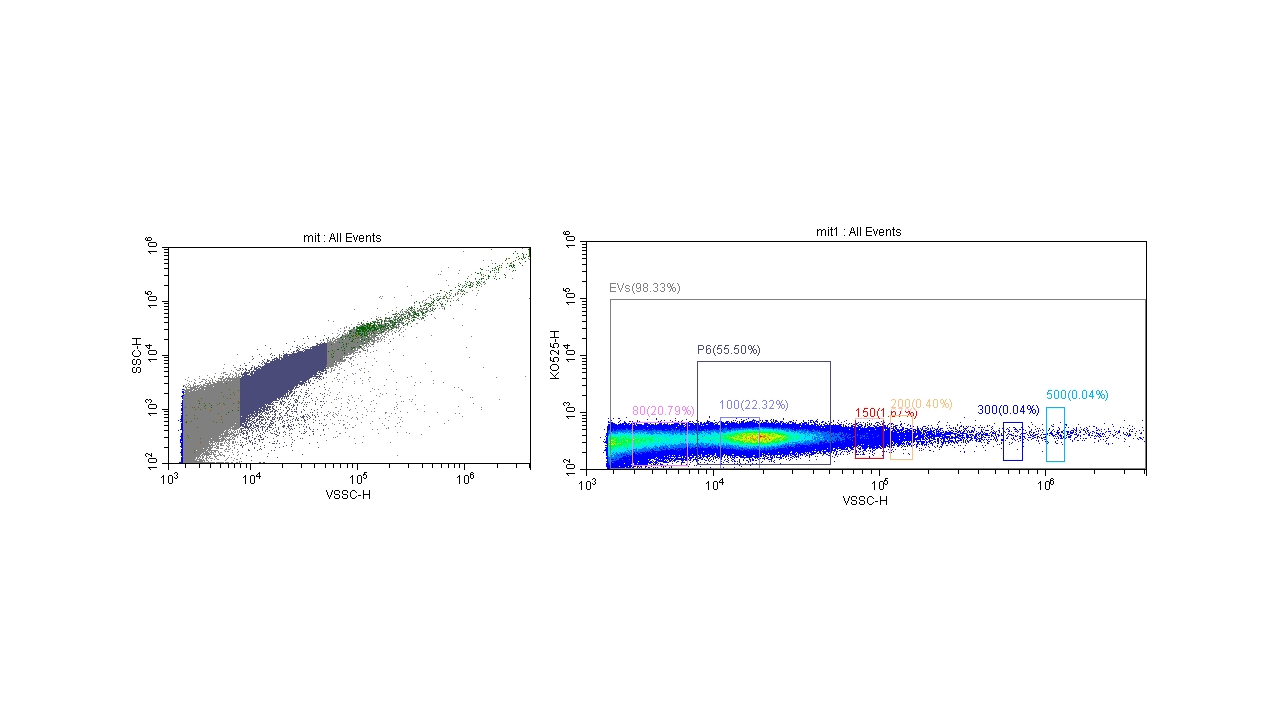


Figure S7. Flow cytometry was used to calculate the number of isolated mitochondria, which were gated in the P6.


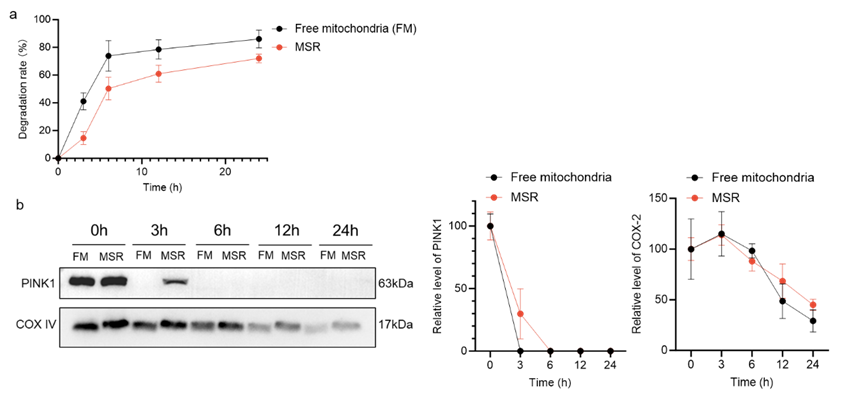


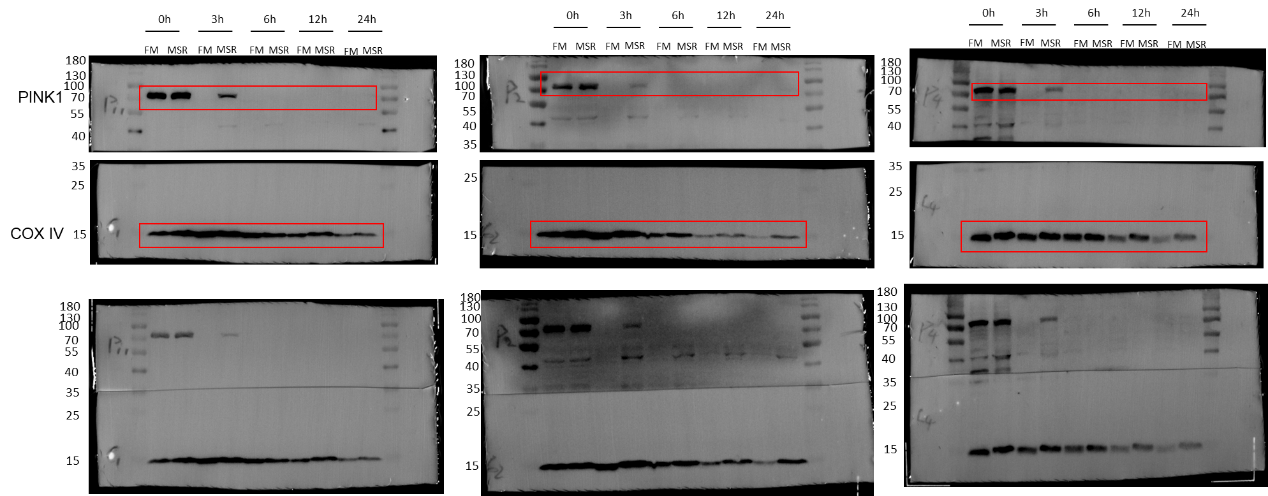


Figure S8. a) The results of evaluating the degradation rate by detecting the total protein concentration at different time points (n = 3 per group). b) Western blot analysis and quantification of PINK1 and COX IV at different time points (n = 3 per group).


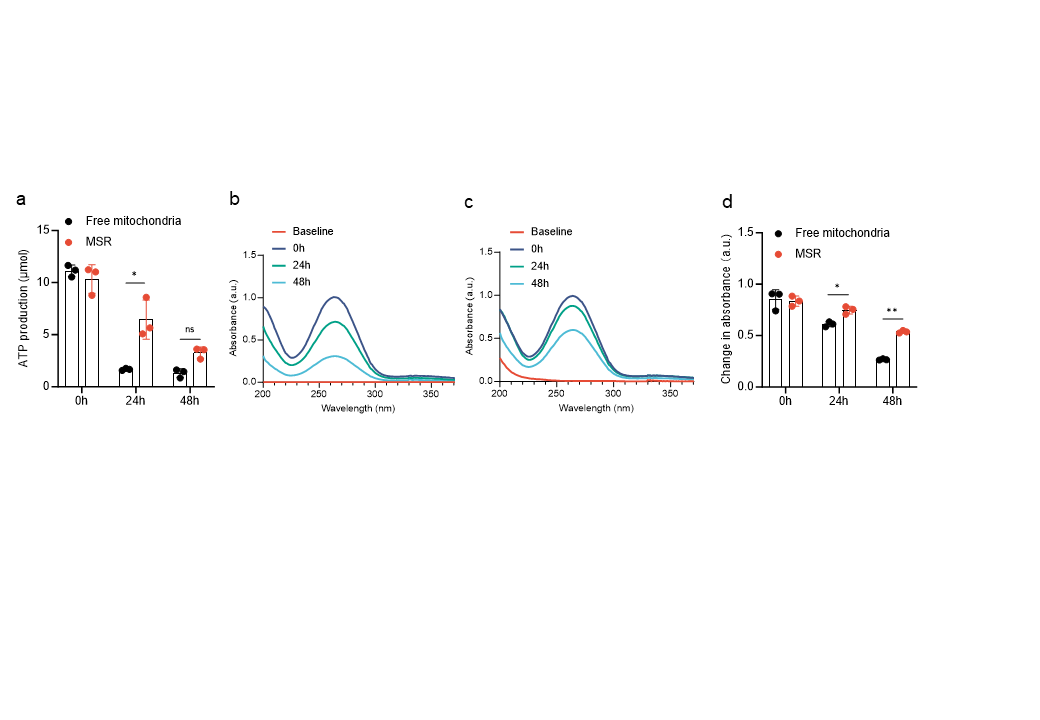


Figure S9. a) ATP synthesis assay of free mitochondria and MSR after being preserved for 24 and 48 hours (t-test; n = 3 per group). b) The UV–vis spectra of NAD^+^ in the free mitochondria group. c) The UV–vis spectra of NAD^+^ in the MSR group. d) Quantitative analysis of NAD^+^ production (t-test; n = 3 per group). Data are presented as mean ± s.d., and n represents biological replicates. *P < 0.05; **P < 0.01; ***P < 0.001; ****P < 0.0001 are considered as statistically significant.


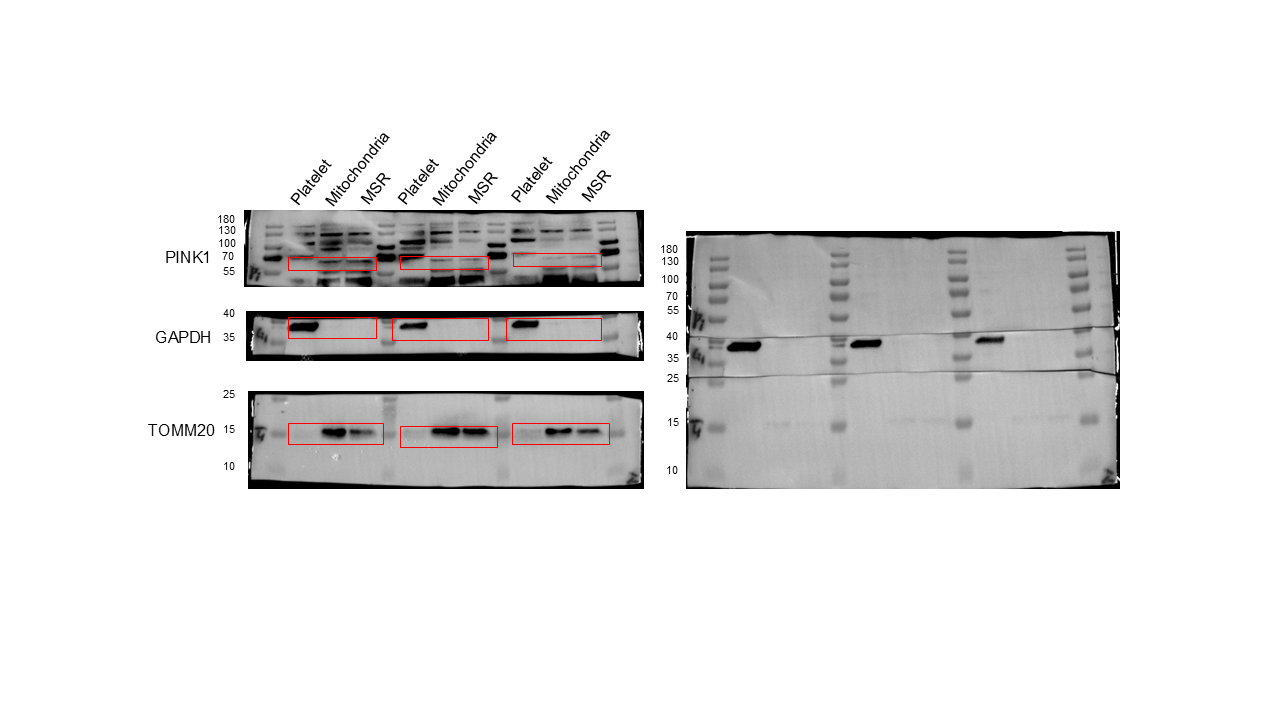


Figure S10. Uncropped Western blot analysis of PINK1 from platelet, isolated mitochondria, and MSR.


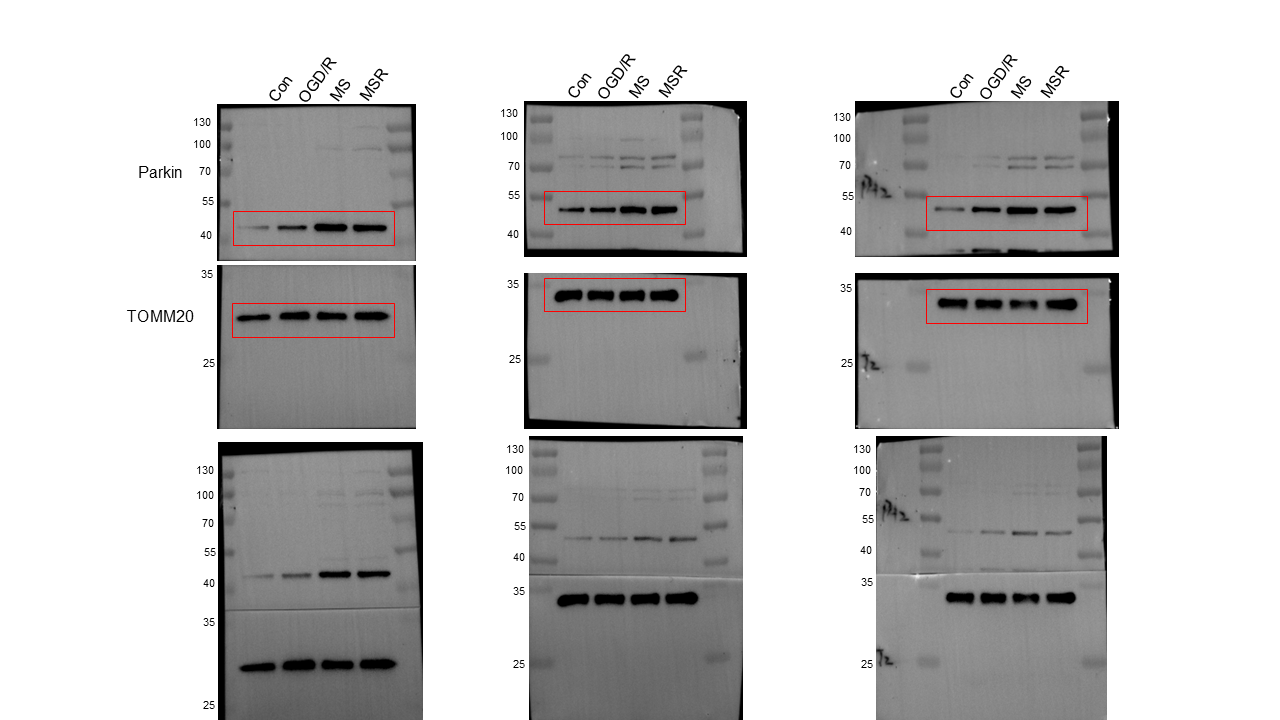


Figure S11. Uncropped Western blot analysis of Parkin from different groups.


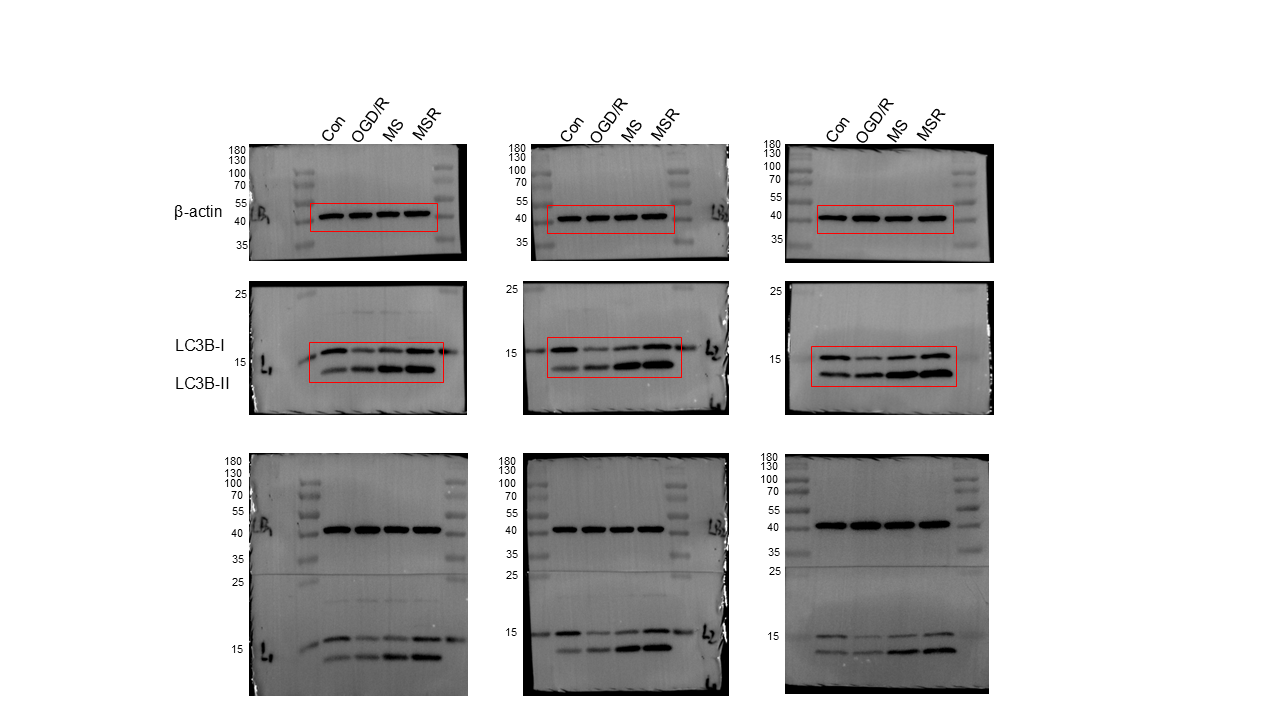


Figure S12. Uncropped Western blot analysis of LC3 from different groups.


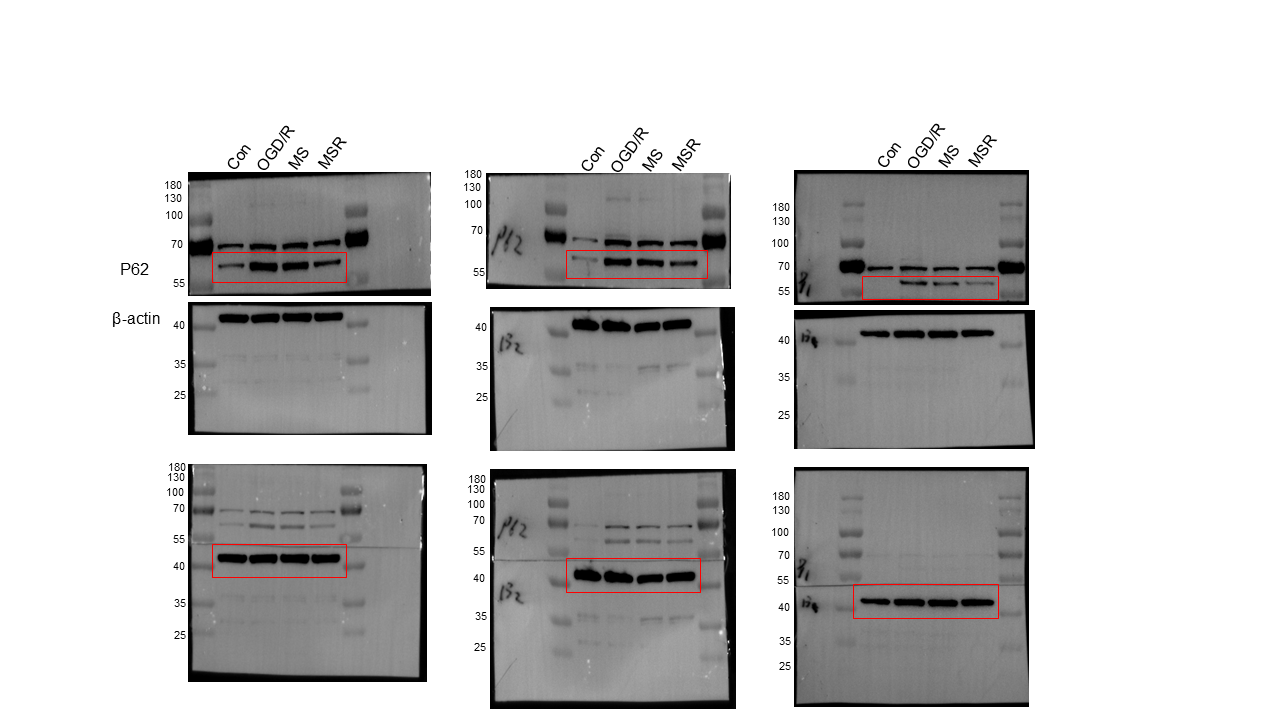


Figure S13. Uncropped Western blot analysis of P62 from different groups.


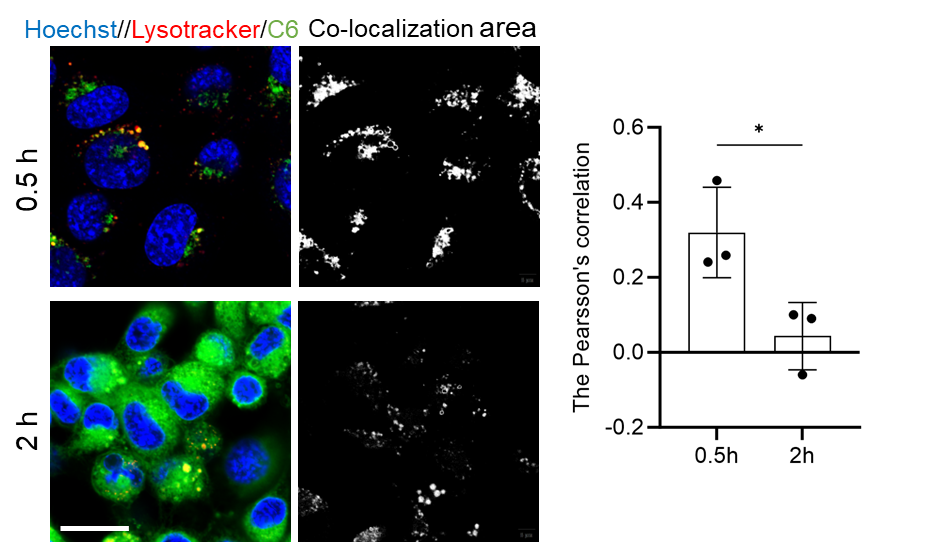


Figure S14. CLSM images for each timepoint to quantify the colocalization of C6-labeled MSR with lysosomes, and the colocalization analysis of C6-labeled MSR with lysosomes using Pearson's Correlation (t-test; n = 3 per group). Scale bars: 20 μm. Data are presented as mean ± s.d., and n are biological replicates. *P < 0.05; **P < 0.01; ***P < 0.001; ****P < 0.0001 are considered as statistically significant.


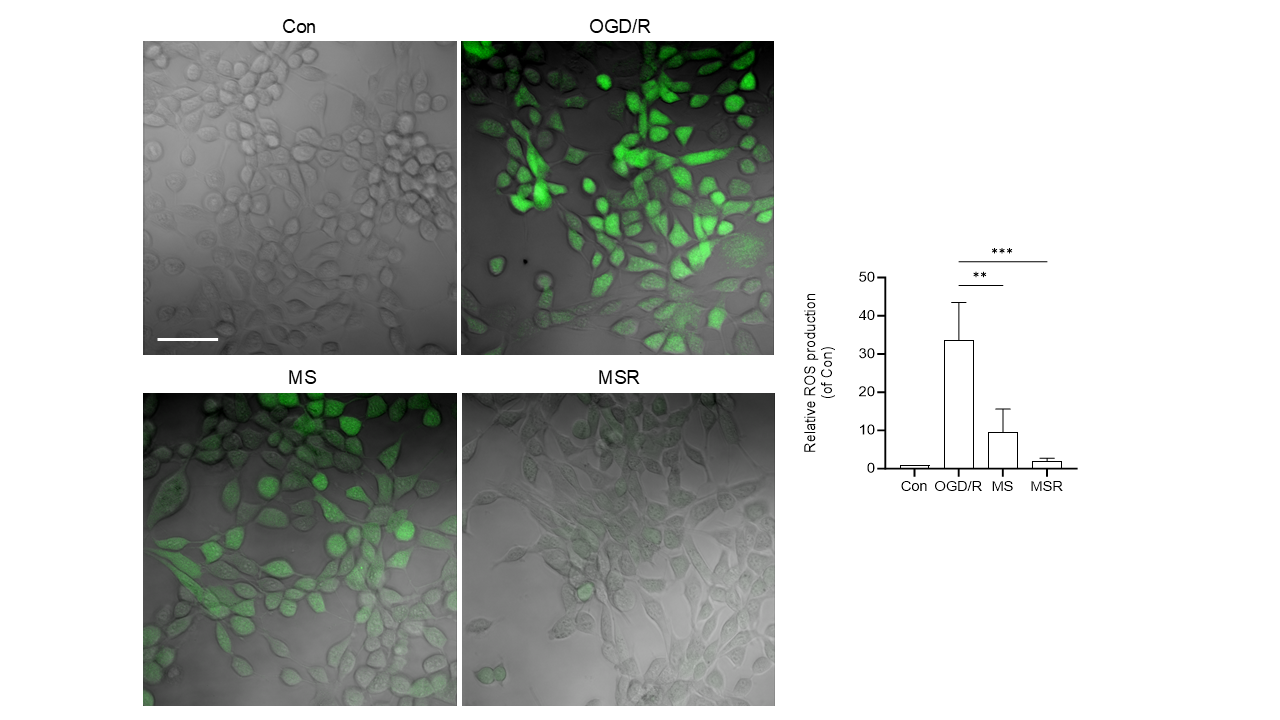


Figure S15. CLSM images showed the ROS clearance using DCFH-DA as fluorescence indicator, and the quantitative analysis of the ROS production (one-way ANOVA; n = 3 per group). Scale bars: 40 μm. Data are presented as mean ± s.d., and n are biological replicates. *P < 0.05; **P < 0.01; ***P < 0.001; ****P < 0.0001 are considered as statistically significant.


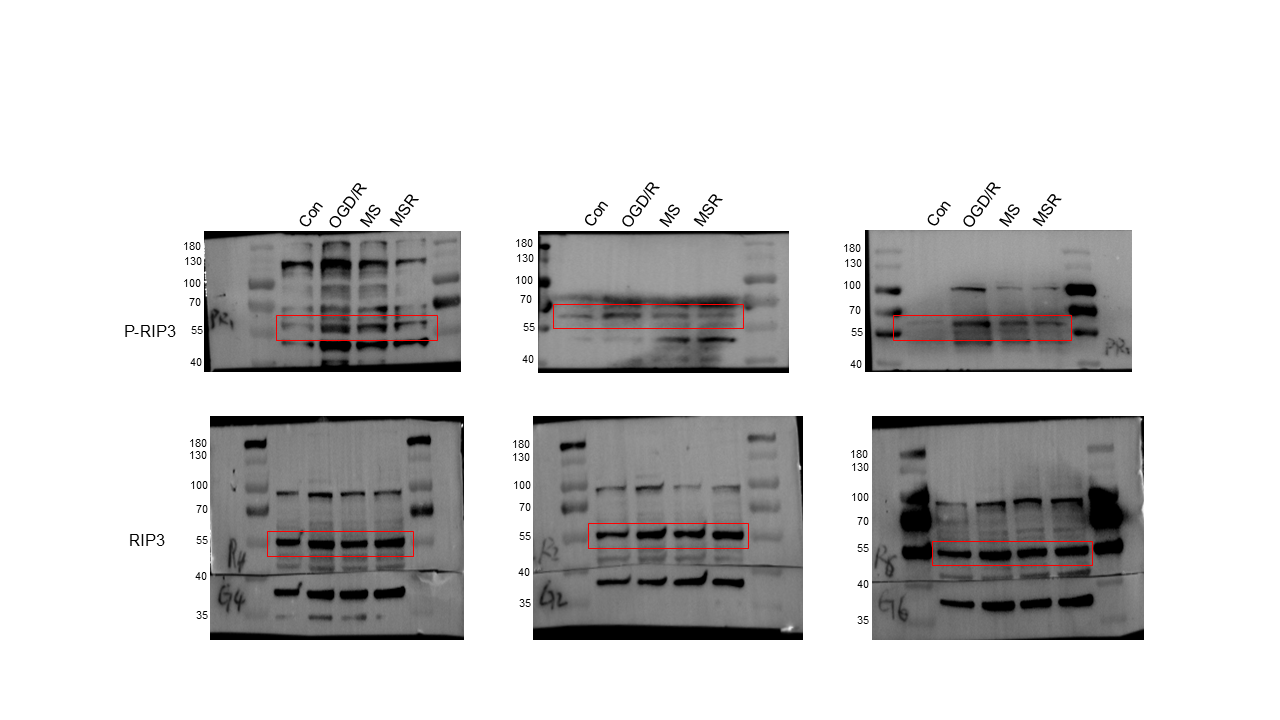


Figure S16. Uncropped Western blot analysis of RIP3 and P-RIP3 from different groups.


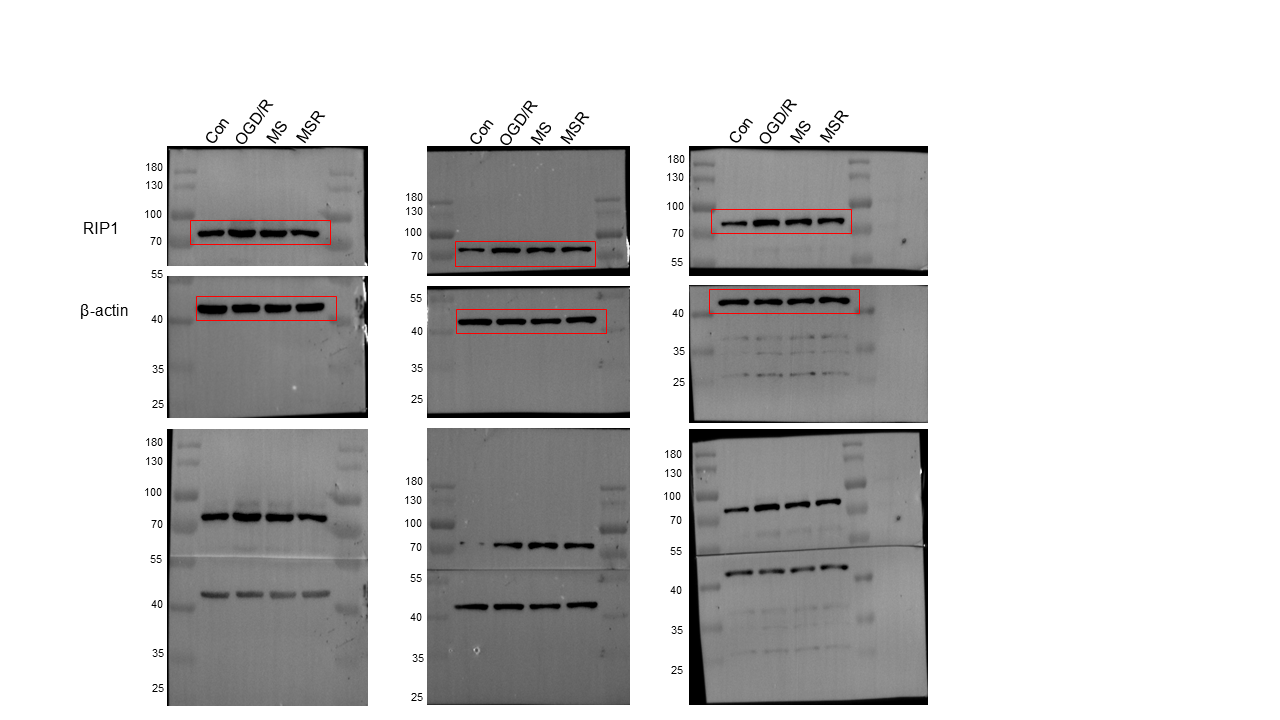


Figure S17. Uncropped Western blot analysis of RIP1 from different groups.


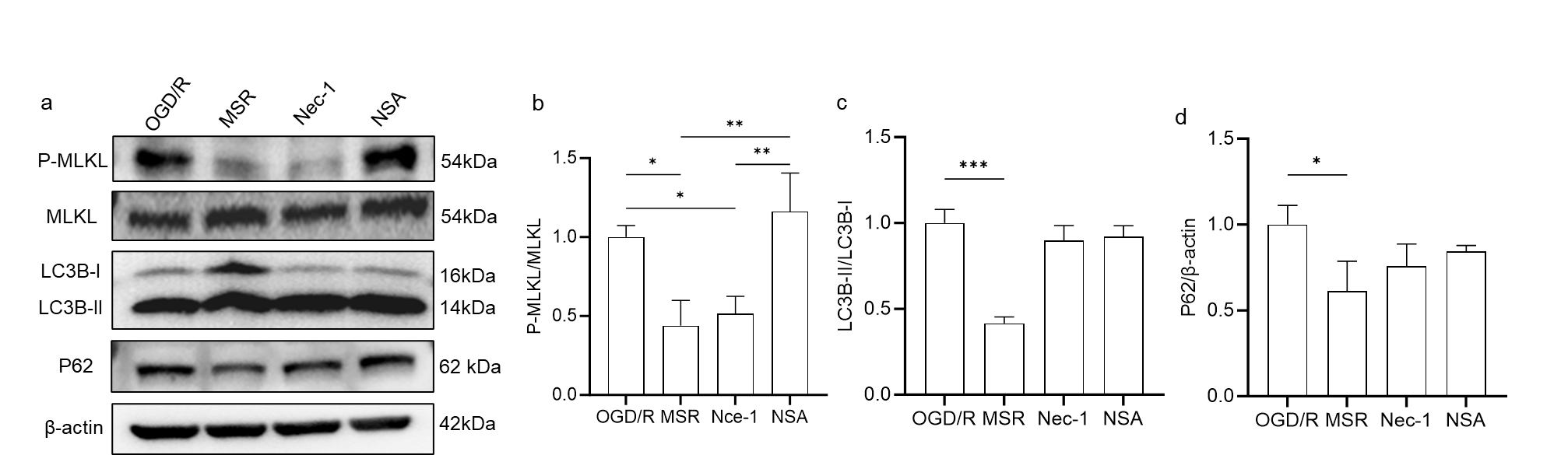


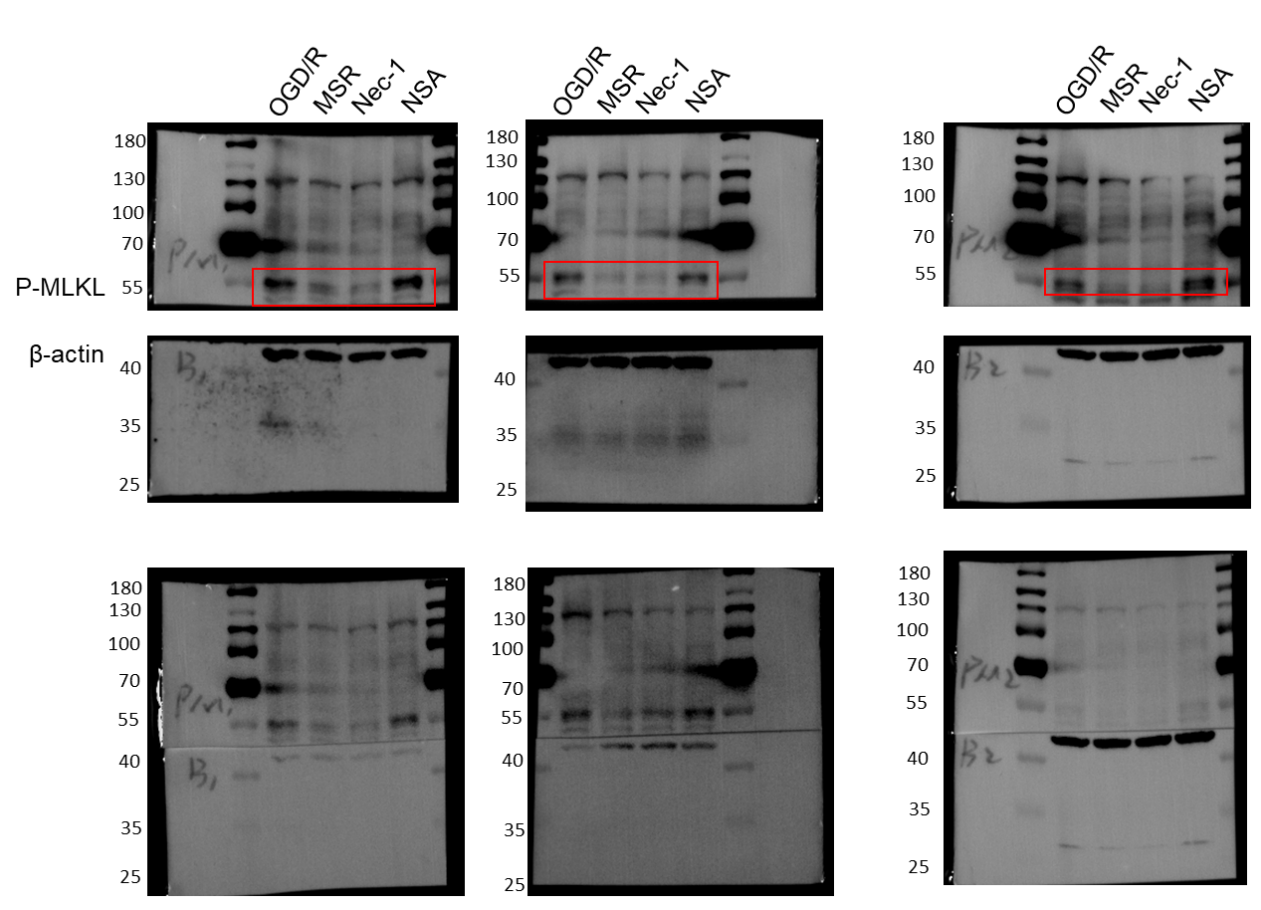


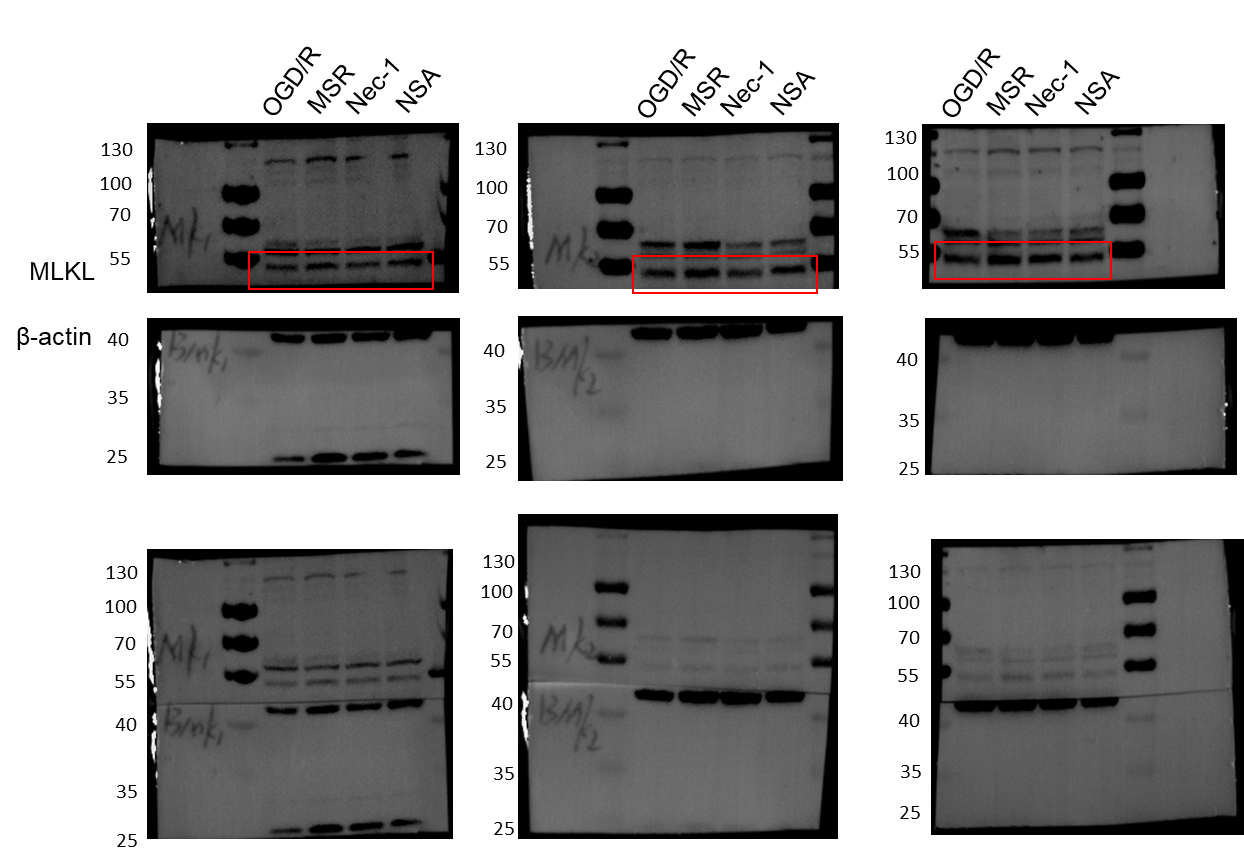


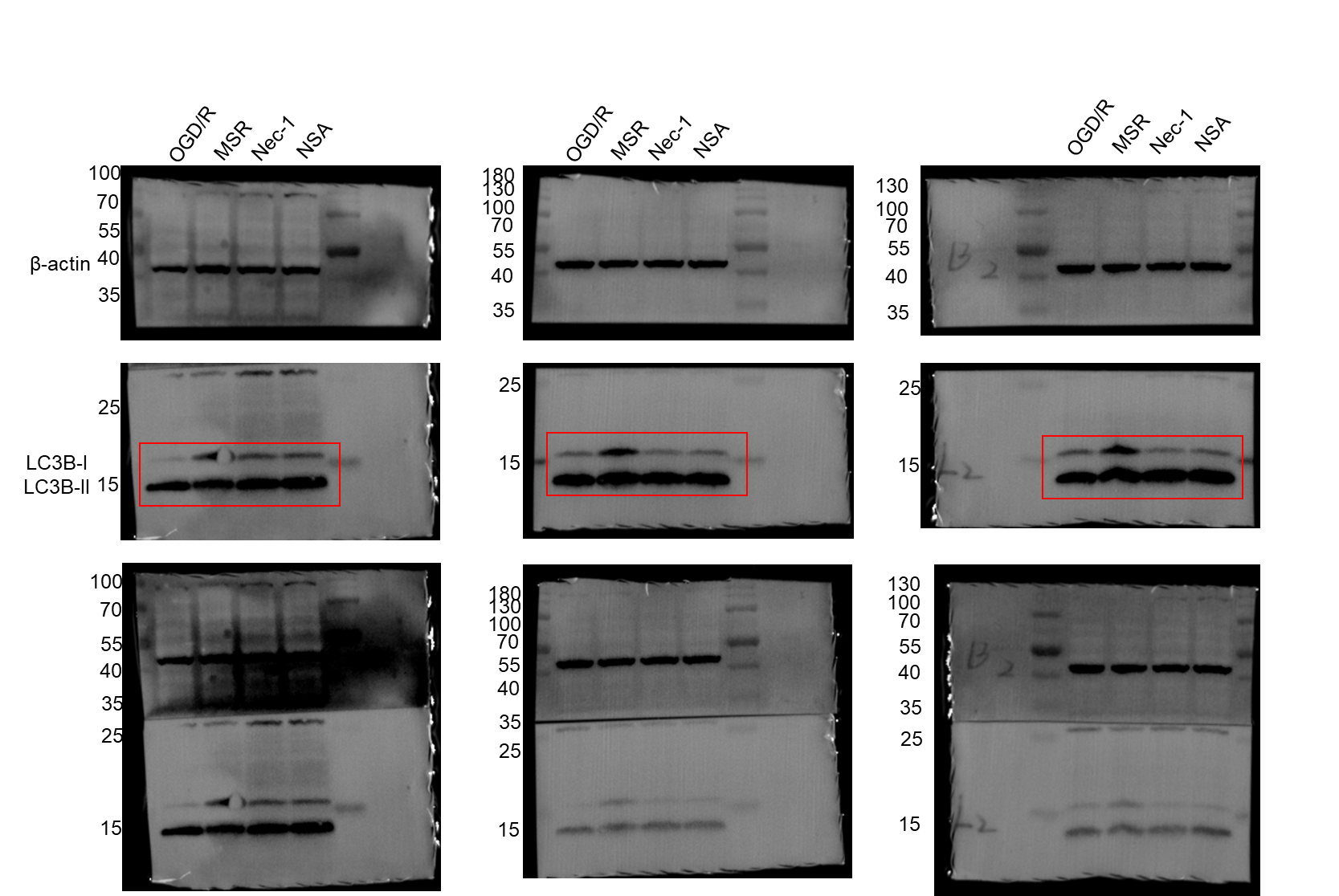


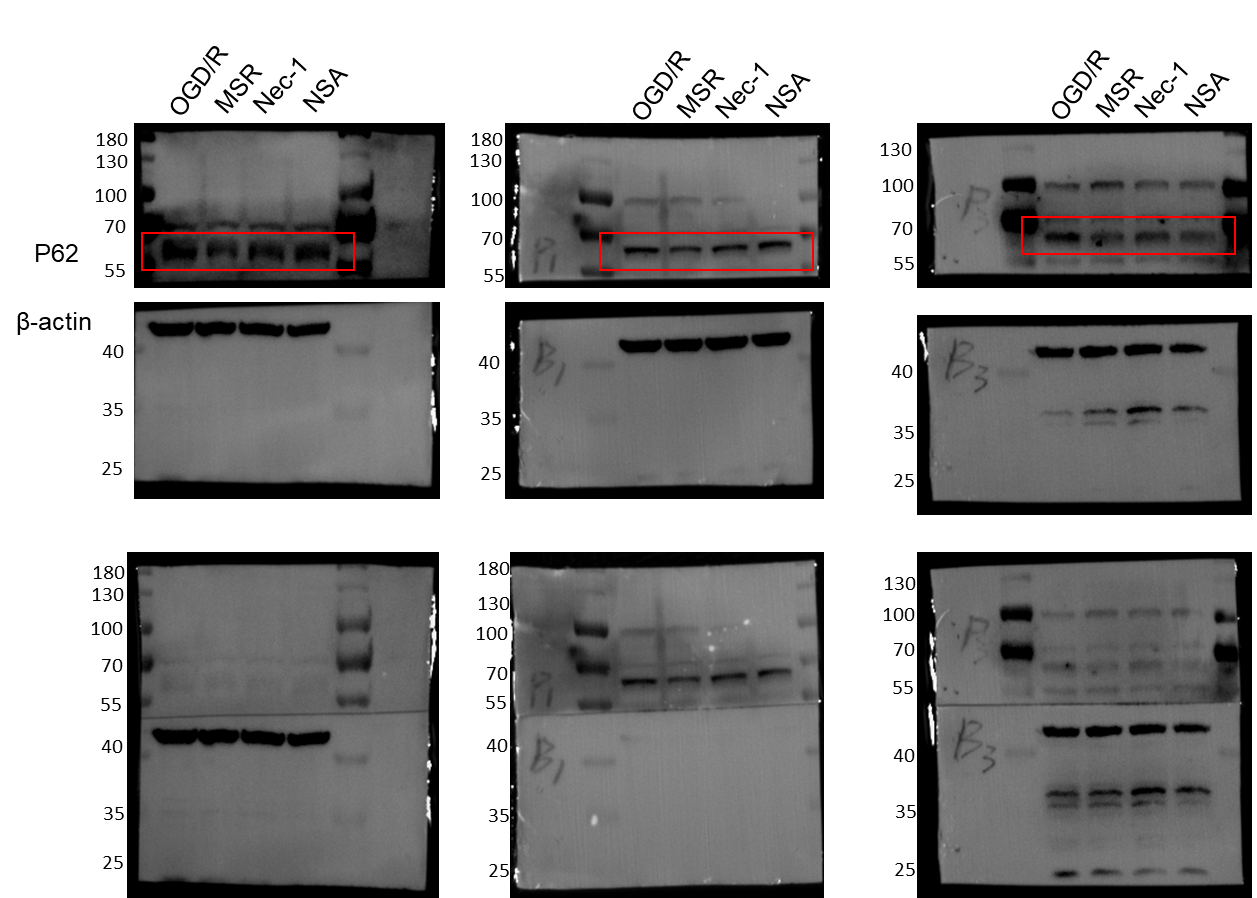


Figure S18. a) Western blot analysis of the protein expression levels related to autophagic flux and necroptosis. b-d) Quantification of expression levels of P-MLKL, ratio of LC3B-II/I, and P62. (one-way ANOVA; n = 3 per group). Data are presented as mean ± s.d., and n represents biological replicates. *P < 0.05; **P < 0.01; ***P < 0.001; ****P < 0.0001 are considered as statistically significant.


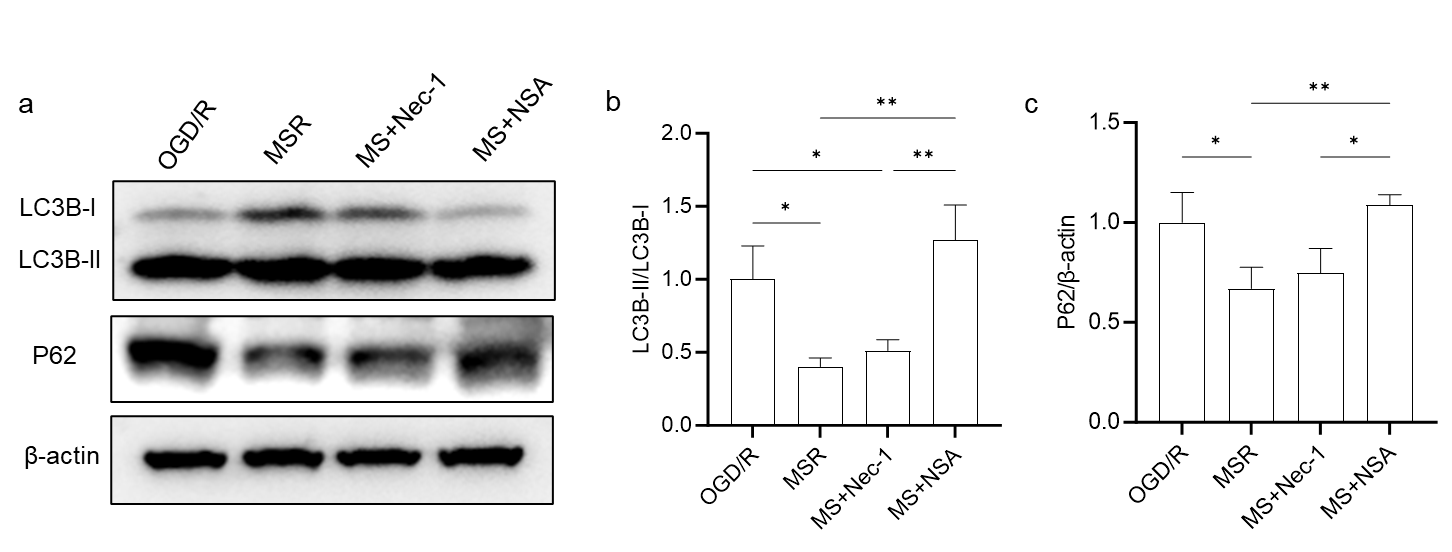


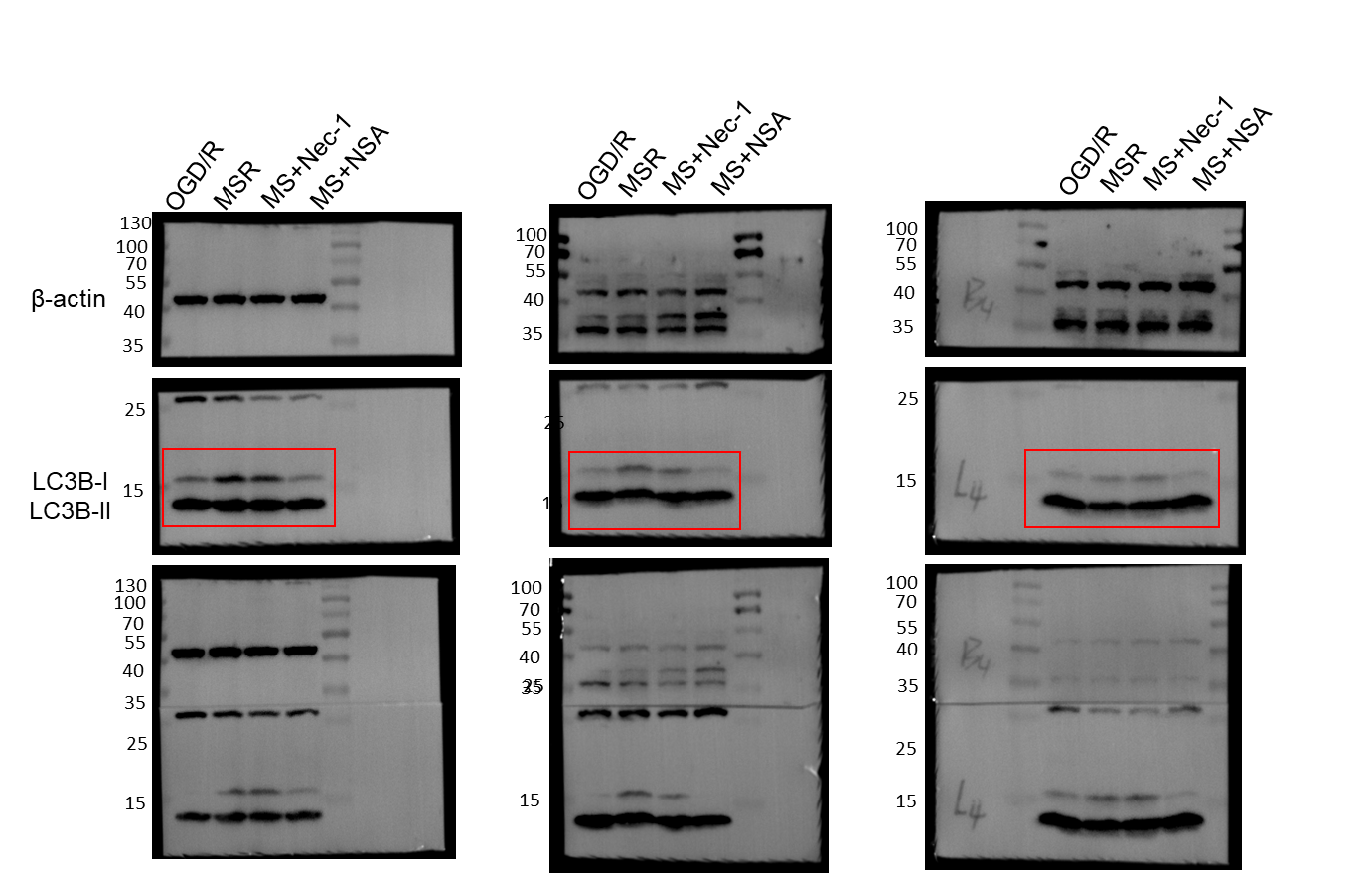


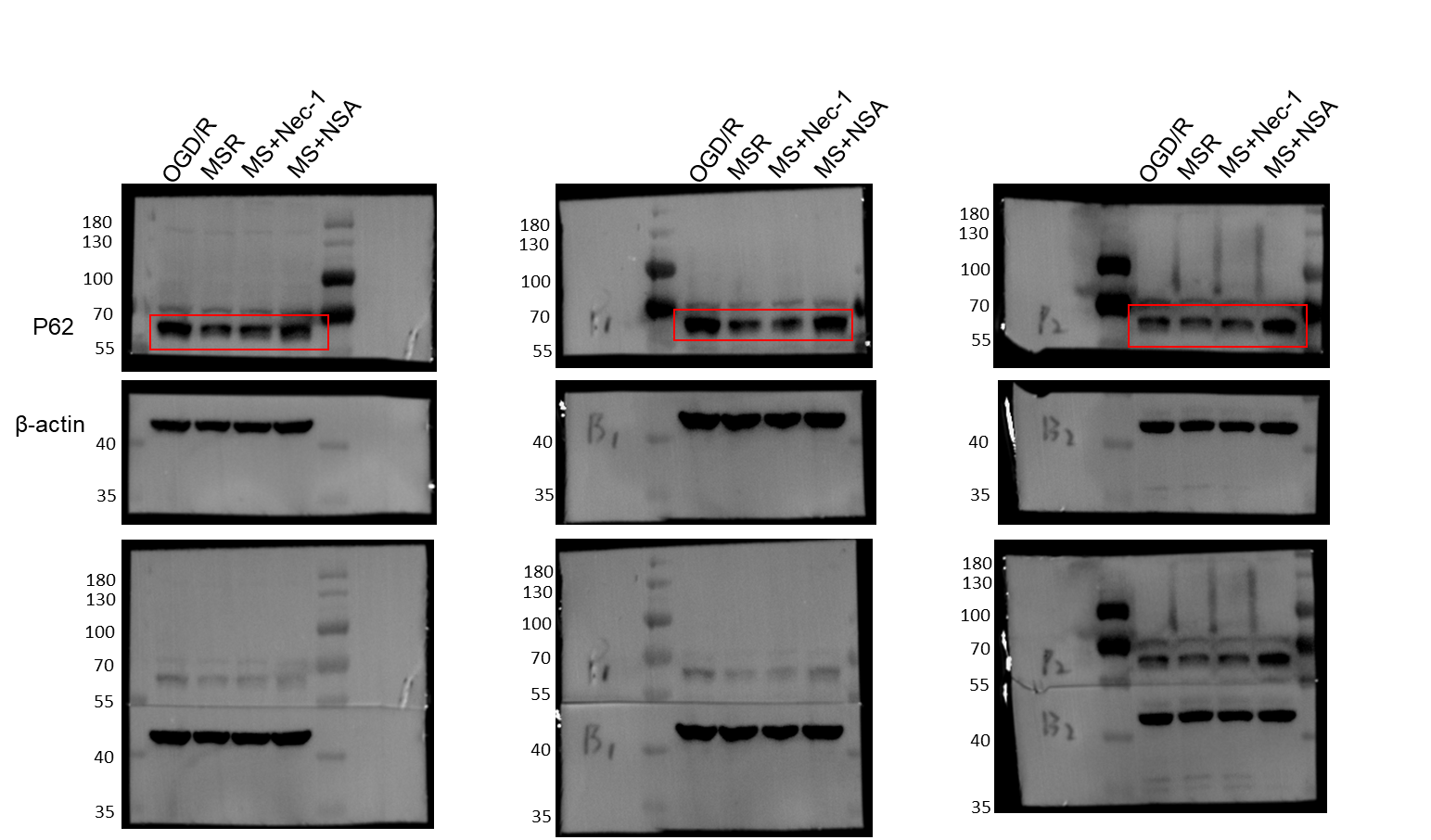


Figure S19. a) Western blot analysis of the protein expression levels related to autophagic flux in groups that combined exogenous mitochondria with necroptosis inhibitor. b, c) Quantification of the ratio of LC3B-II/I and P62. (one-way ANOVA; n = 3 per group). Data are presented as mean ± s.d., and n represents biological replicates. *P < 0.05; **P < 0.01; ***P < 0.001; ****P < 0.0001 are considered as statistically significant.


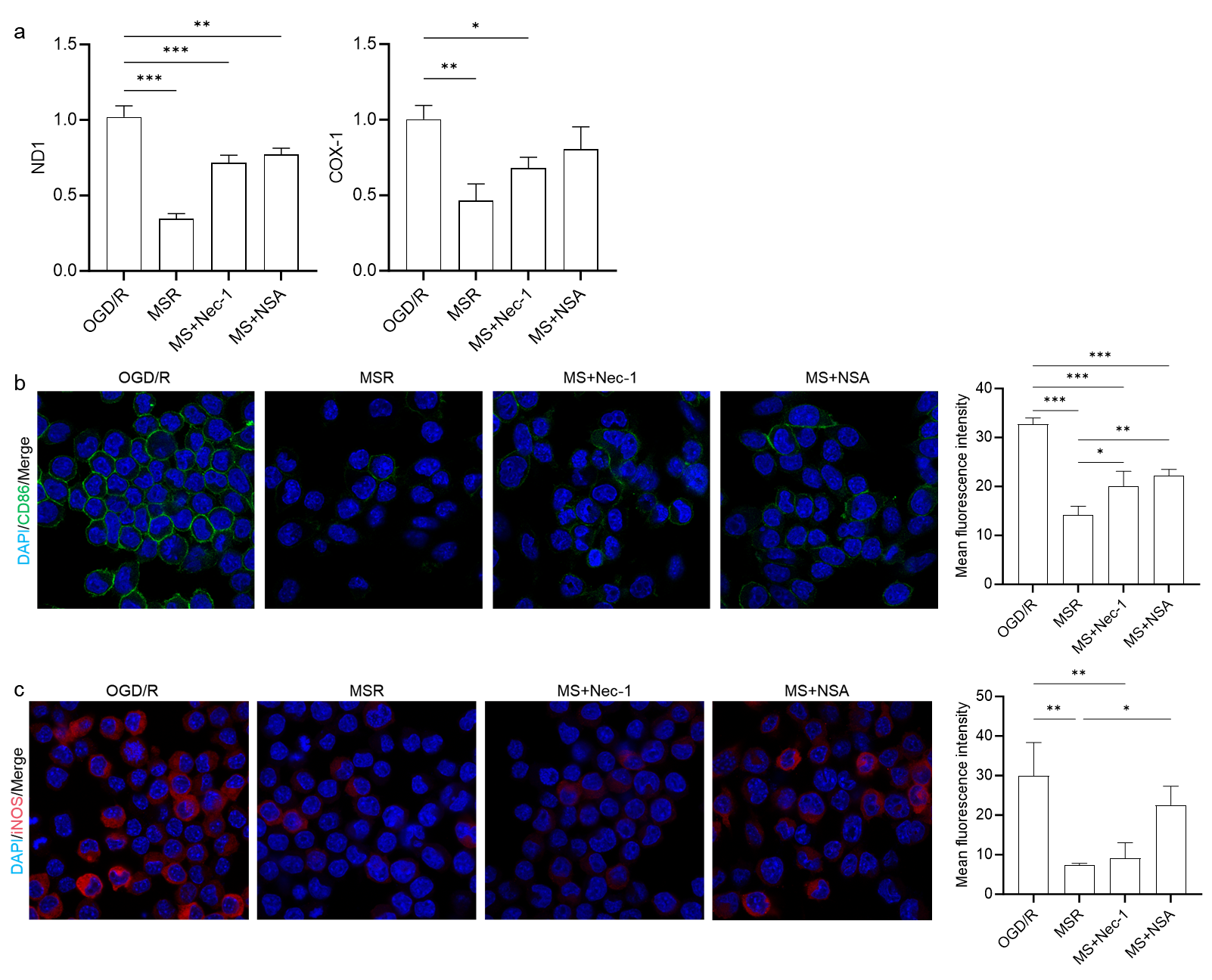


Figure S20. a) Quantification of the extracellular mtDNA levels in groups that combined exogenous mitochondria with necroptosis inhibitor (one-way ANOVA; n = 3 per group). b) The expression and the quantitative analysis of CD86 (M1 phenotypic marker) in groups that combined exogenous mitochondria with necroptosis inhibitor after the co-culture model (one-way ANOVA; n = 3 per group). c) The expression and the quantitative analysis of iNOS (M1 phenotypic marker) in groups that combined exogenous mitochondria with necroptosis inhibitor after the co-culture model (one-way ANOVA; n = 3 per group). Data are presented as mean ± s.d., and n represents biological replicates. *P < 0.05; **P < 0.01; ***P < 0.001; ****P < 0.0001 are considered as statistically significant.


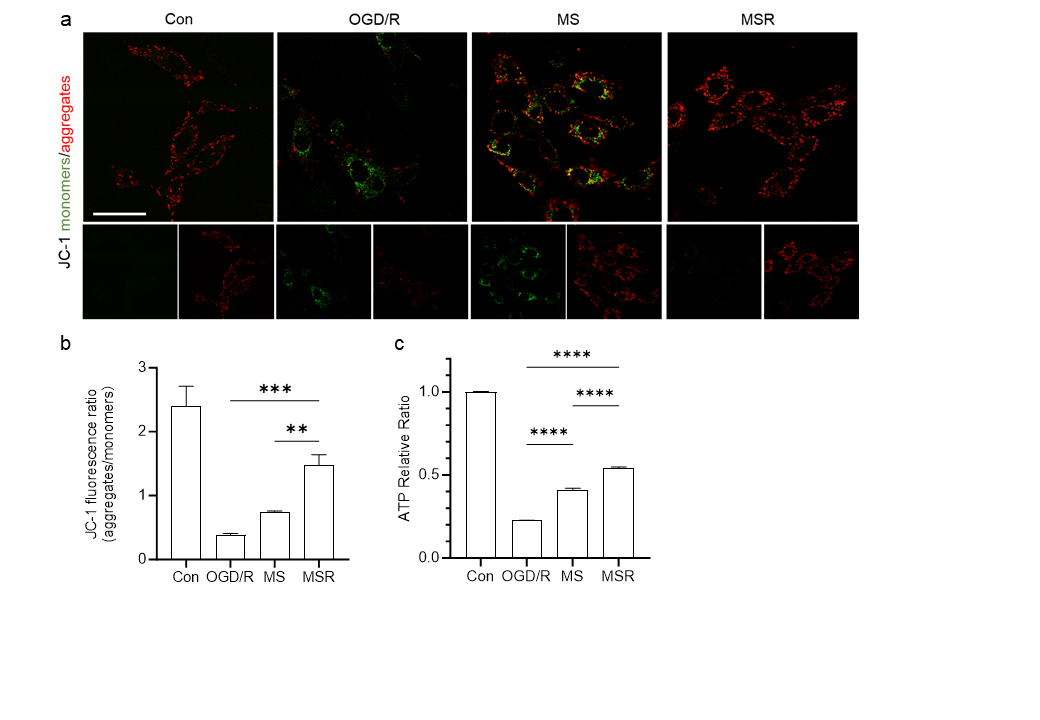


Figure S21. a) The CLSM images of mitochondrial membrane potential (MMP) from different treatment groups that assessed by JC-1 assays (Green: monomer; Red: aggregates). Scale bar: 50 μm. b) Quantification of the ratio of aggregates/monomers. (one-way ANOVA; n = 3 per group). c) Quantification of ATP production from different treatment groups (one-way ANOVA; n = 3 per group). Data are presented as mean ± s.d., and n are biological replicates. *P < 0.05; **P < 0.01; ***P < 0.001; ****P < 0.0001 are considered as statistically significant.


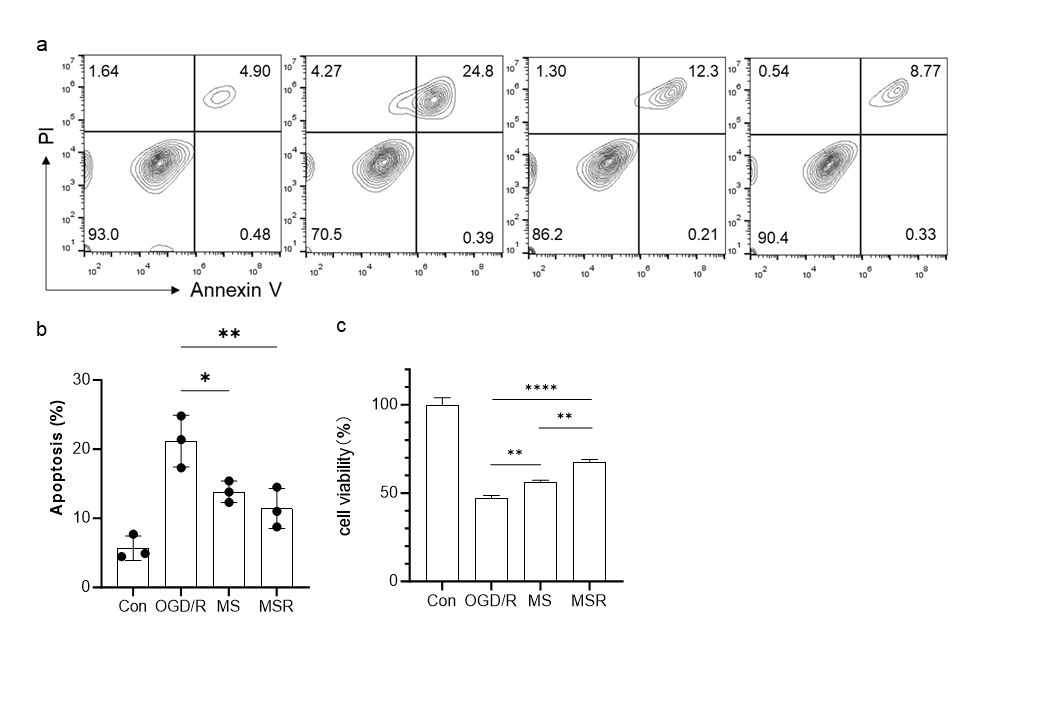


Figure S22. a, b) Flow cytometry analysis and quantification of apoptosis results from different treatment groups (one-way ANOVA; n = 3 per group). c) Cell viability of HT22 cells after different treatments (one-way ANOVA; n = 3 per group). Data are presented as mean ± s.d., and n are biological replicates. *P < 0.05; **P < 0.01; ***P < 0.001; ****P < 0.0001 are considered as statistically significant.


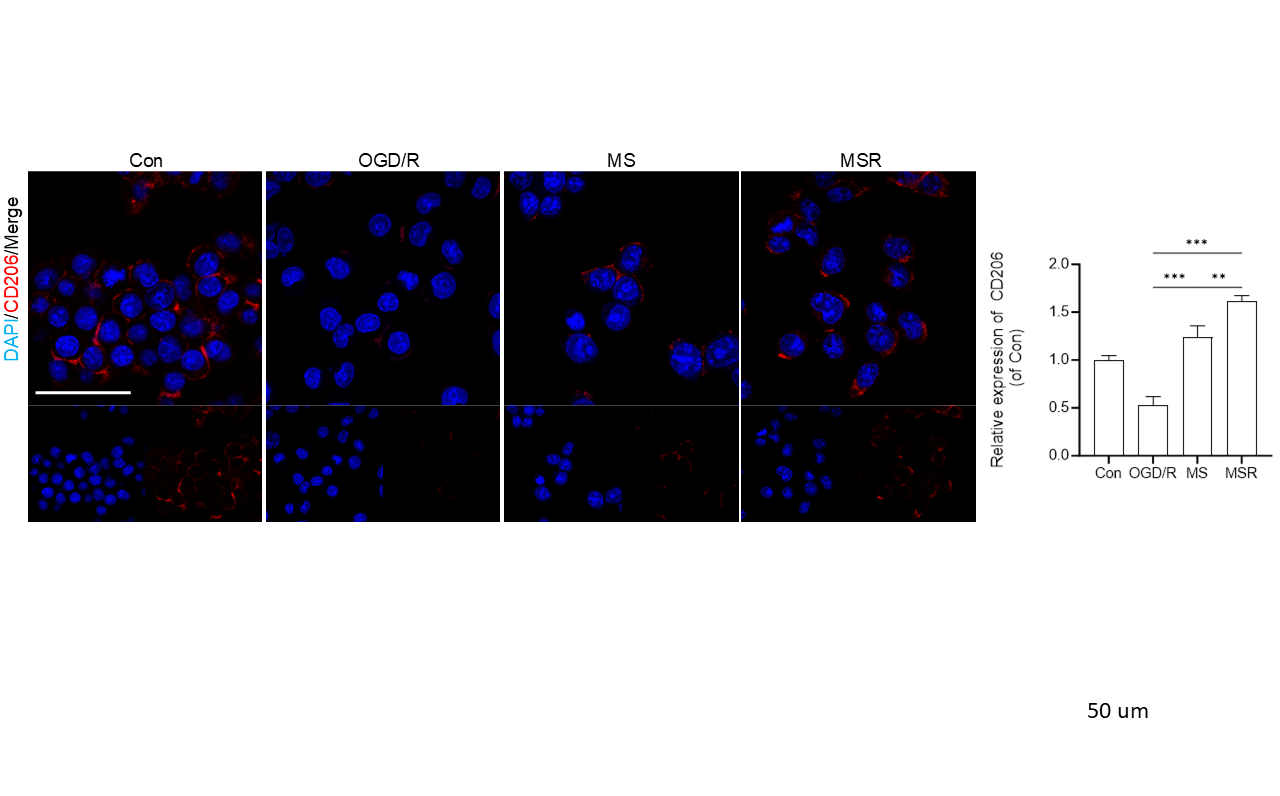


Figure S23. CLSM images showed the changes of CD206 (M2 phenotypic marker) expression after the co-culture model and the quantitative analysis (one-way ANOVA; n = 3 per group). Scale bar: 50 μm. Data are presented as mean ± s.d., and n are biological replicates. *P < 0.05; **P < 0.01; ***P < 0.001; ****P < 0.0001 are considered as statistically significant.


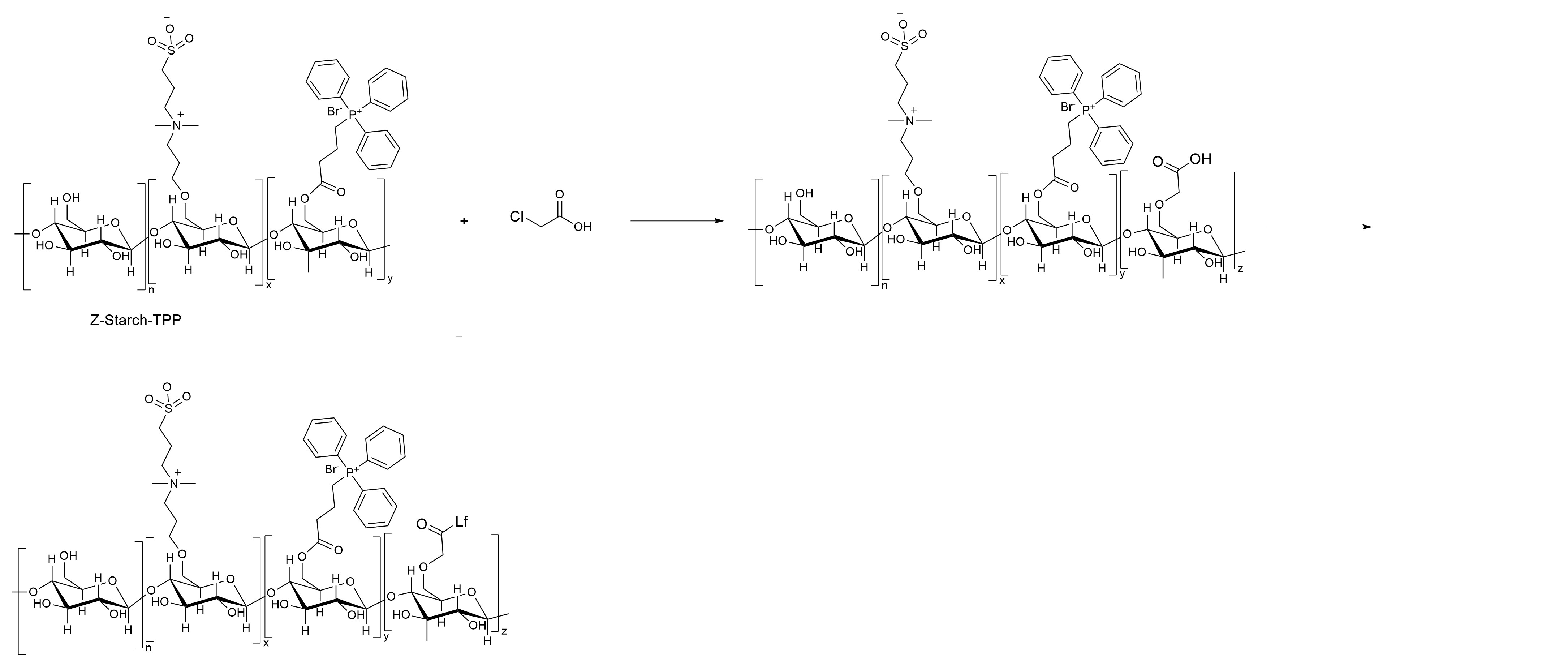


Figure S24. The synthesis route of Lf-modified Z-Starch-TPP.


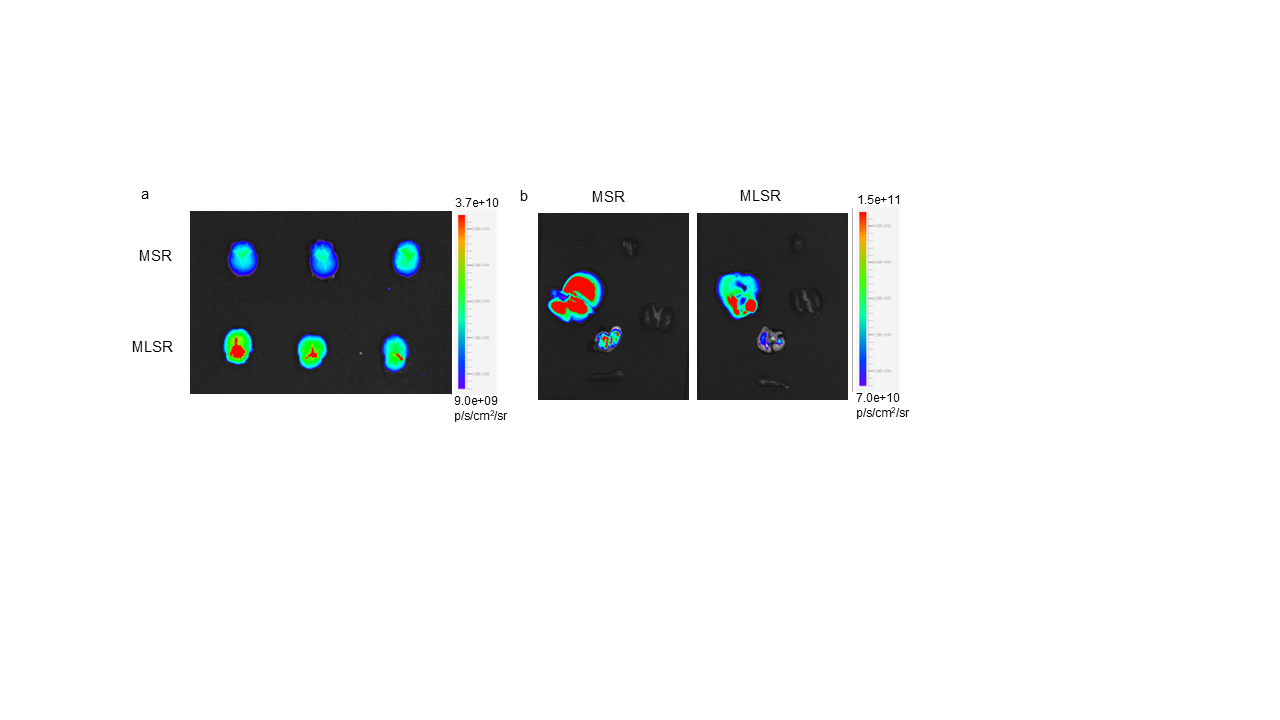


Figure S25. a, b) In vivo imaging of ex vivo brain and major organs 3 h post injection.


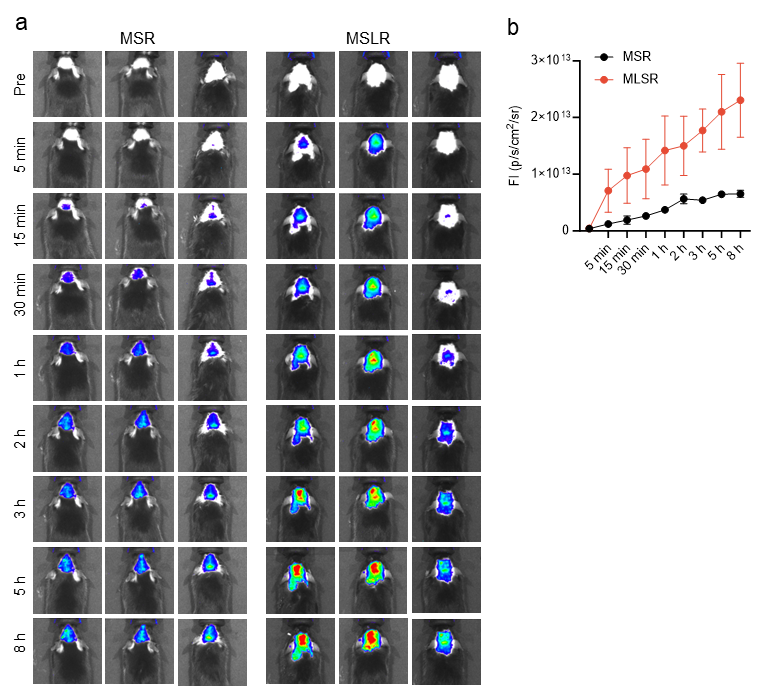


Figure S26. a) In vivo fluorescence imaging of Mitotracker-labeled MSR or MLSR in the brain region (n = 3 per group). b) Fluorescence quantification in the brain region following injection (n = 3 per group).


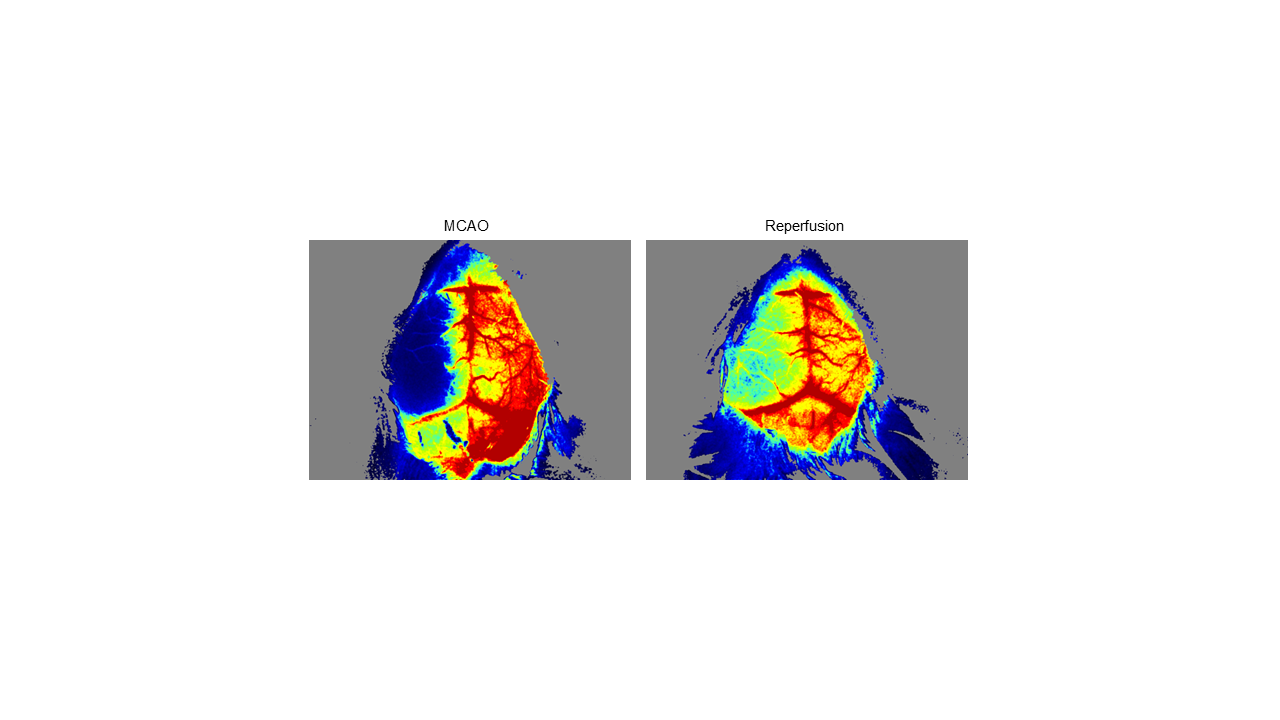


Figure S27. Analysis of the cerebral blood flow in the ischemia phase and reperfusion phase in tMCAO model mice.


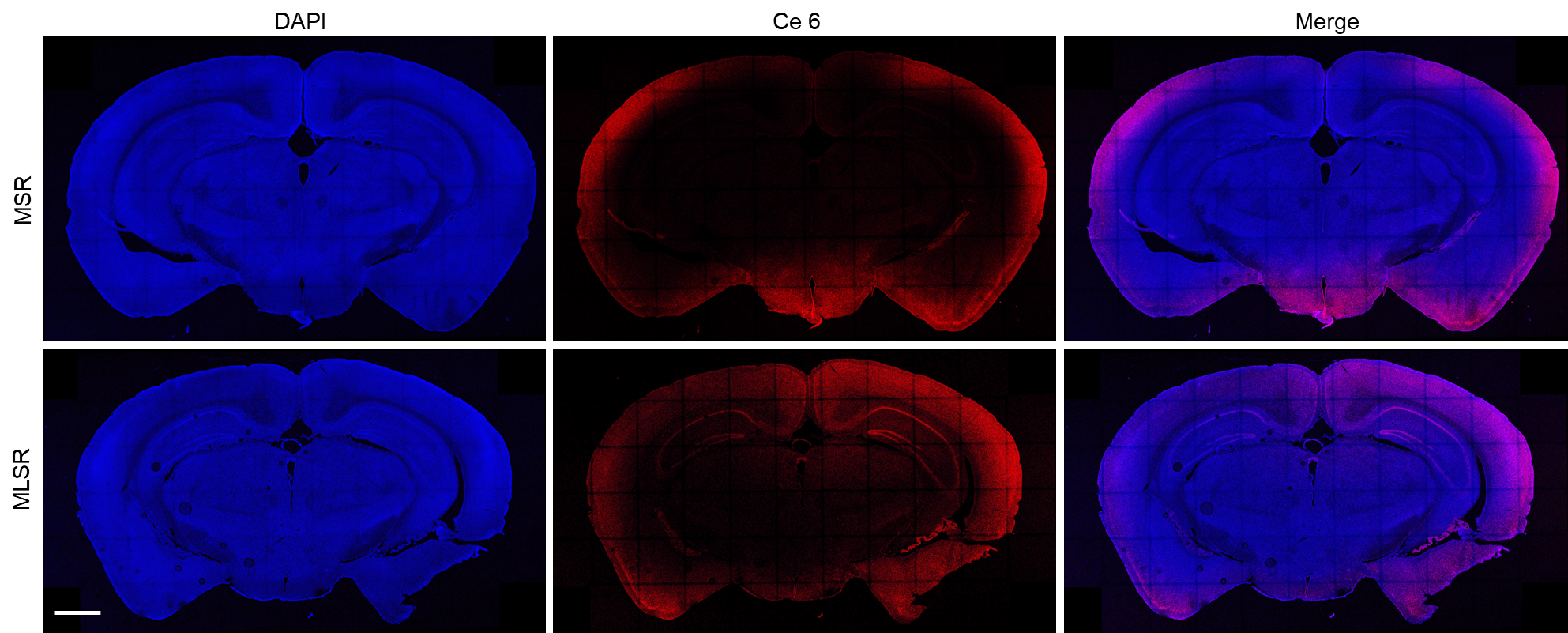


Figure S28. CLSM images showed a greater accumulation of Ce6-labeled MLSR in the ischemic penumbra after intravenous injection (right side). Scale bar: 1 mm.


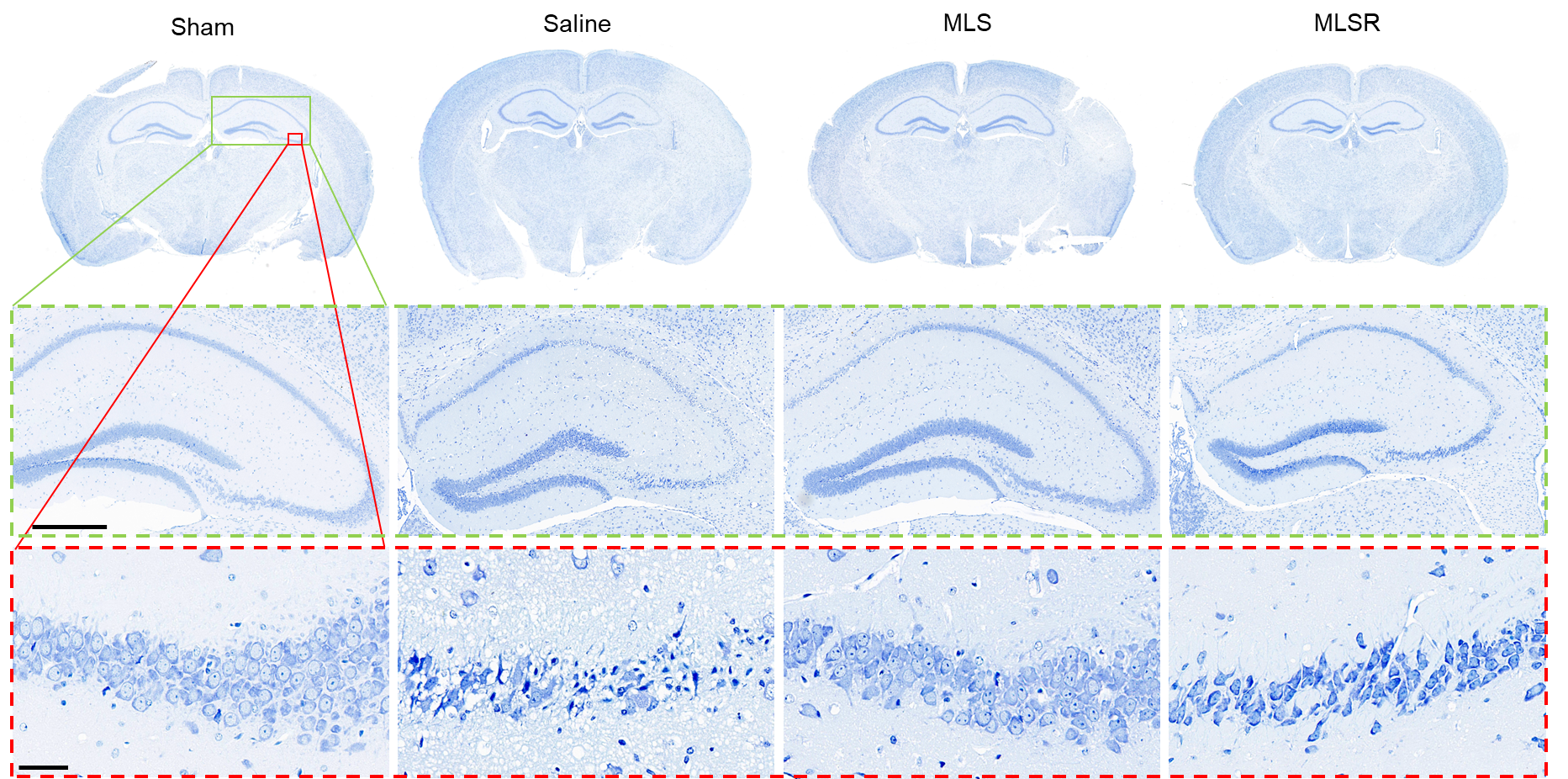


Figure S29. Nissl staining of brain slices in each group. scale bar: 500 μm and 50 μm.


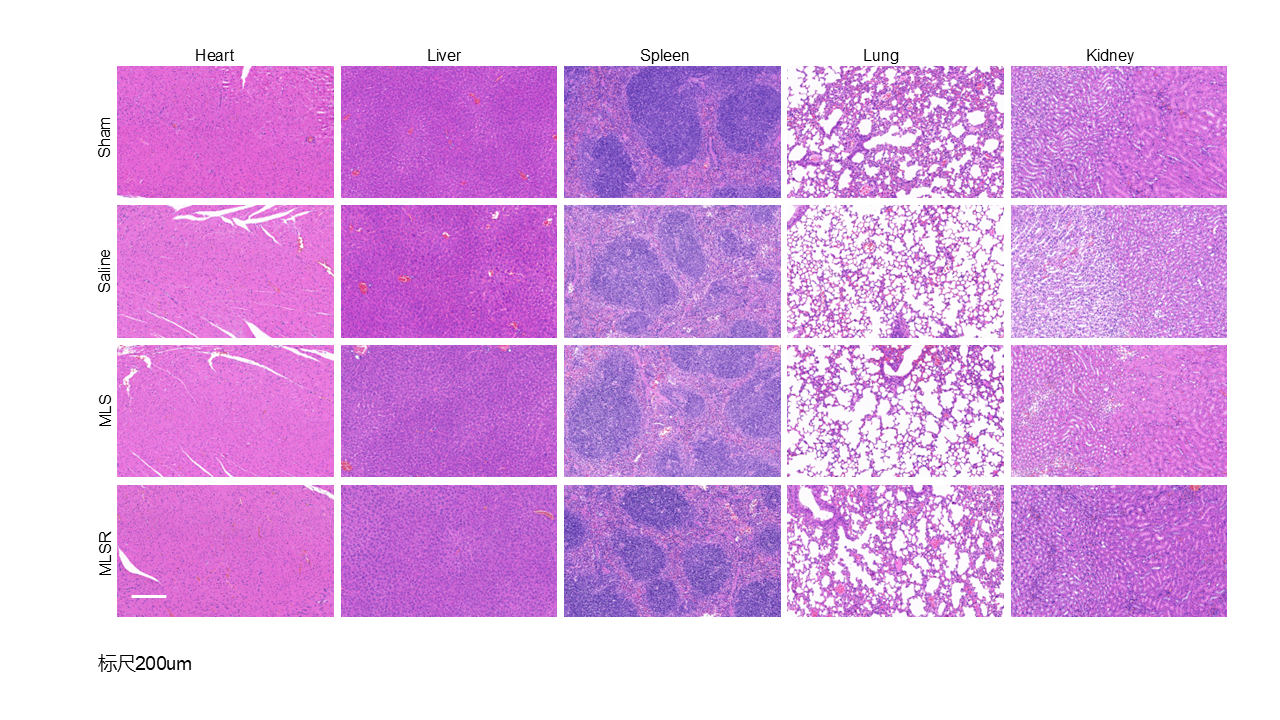


Figure S30. HE staining images of heart, liver, spleen, lung, and kidney of tMCAO mice treated with different treatments. Scale bar: 200 μm.


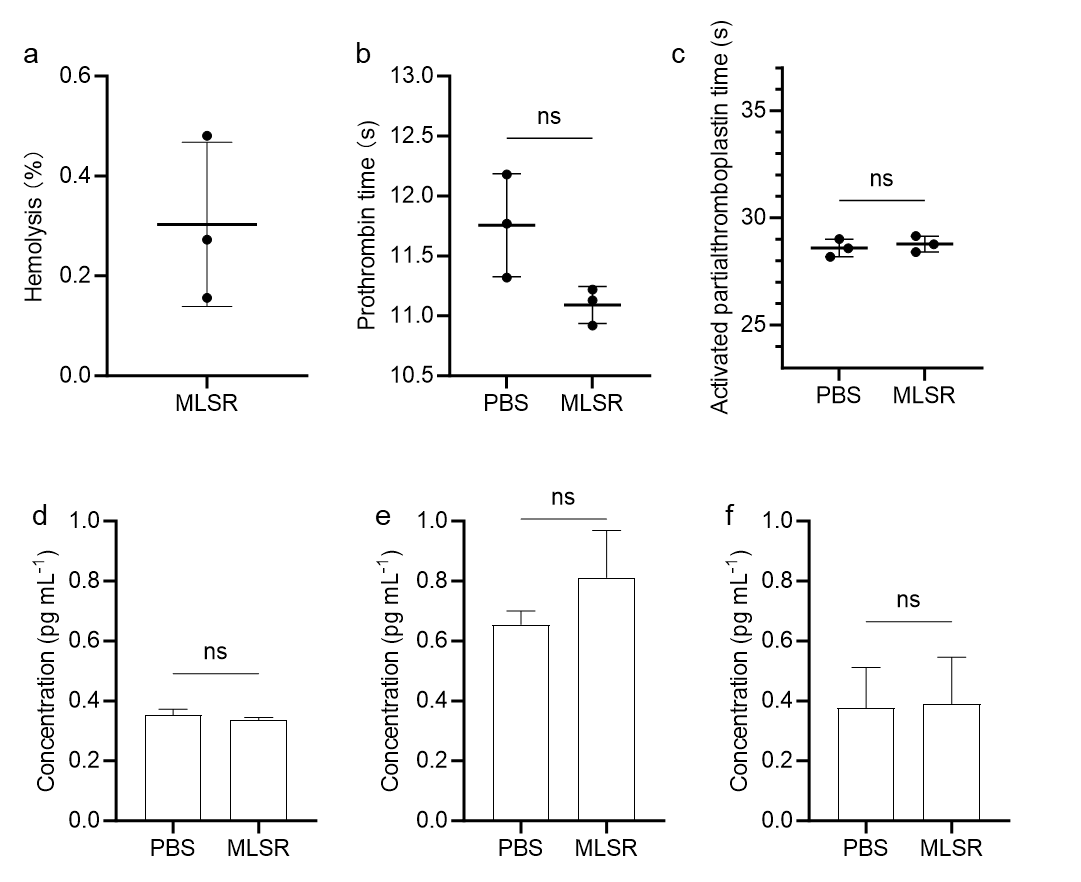


Figure S31. a) The hemolysis assays. b, c) The coagulation function tests include prothrombin time and activated partialthromboplastin time. d-f) Serum cytokines after PBS or MLSR administration, including TNF-α (d), IL-6 (e), and IL-1β (f).


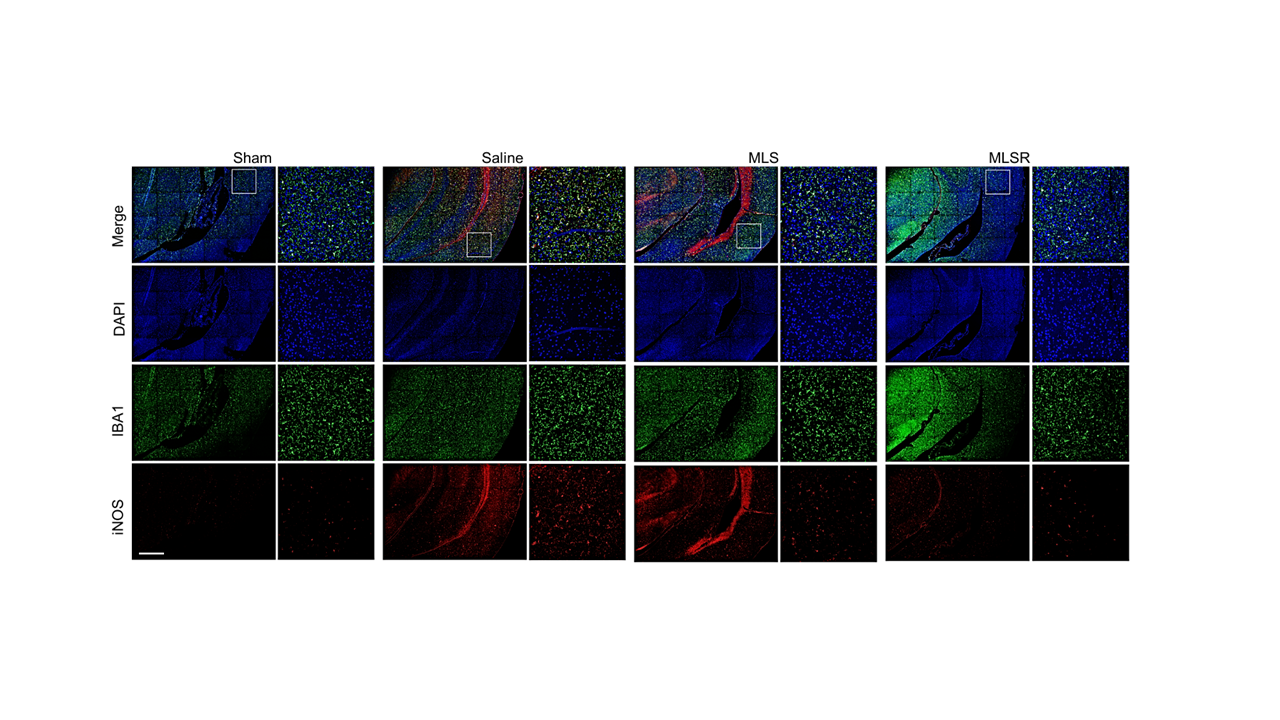


Figure S32. Immunofluorescence staining images of Iba1⁺/iNOS⁺ microglia in ischemic brains (cortex) of tMCAO mice after various treatments. Scale bar: 2.5mm.


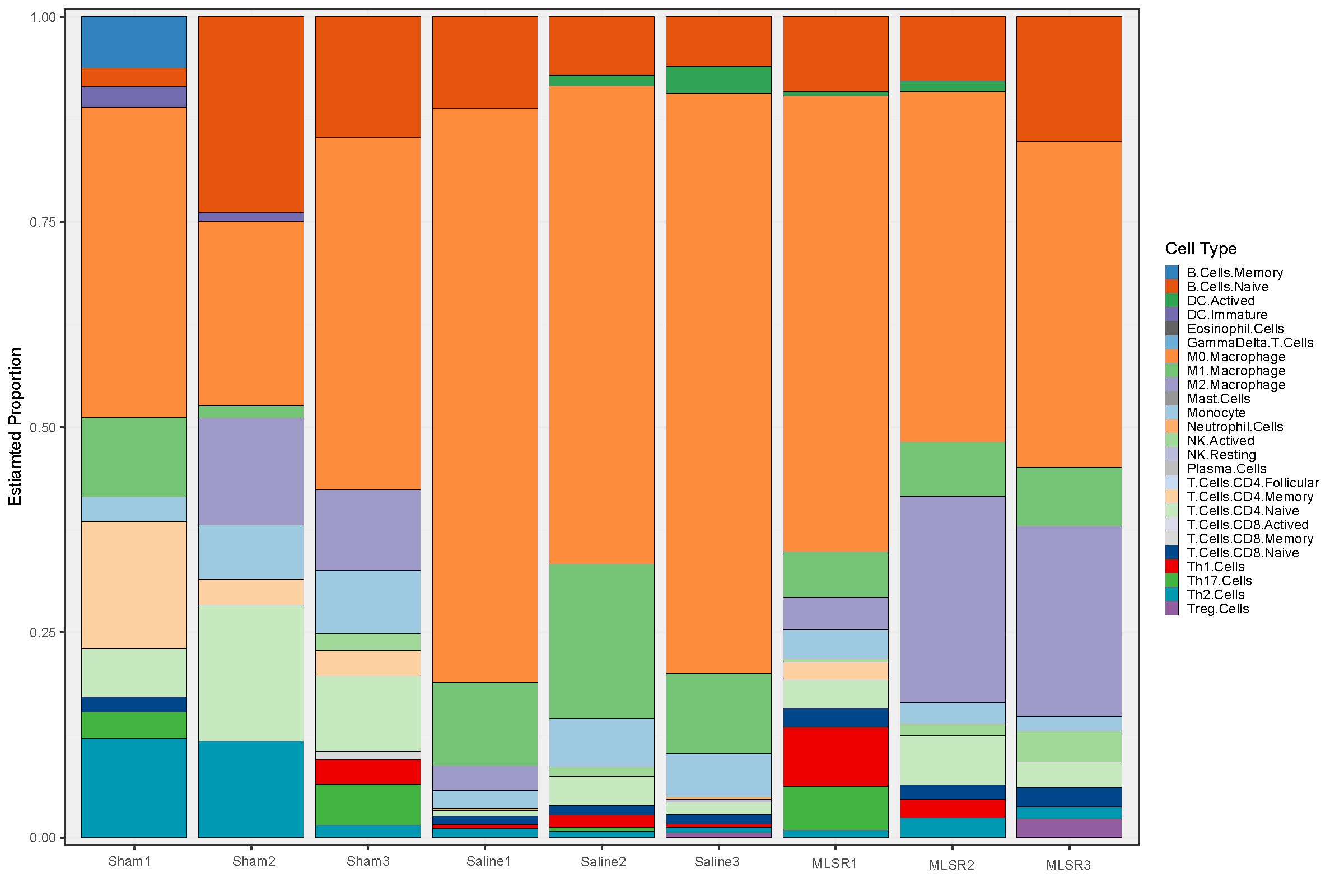


Figure S33. Proportion of each immune cells between the mice in Sham, Saline, and MLSR group.


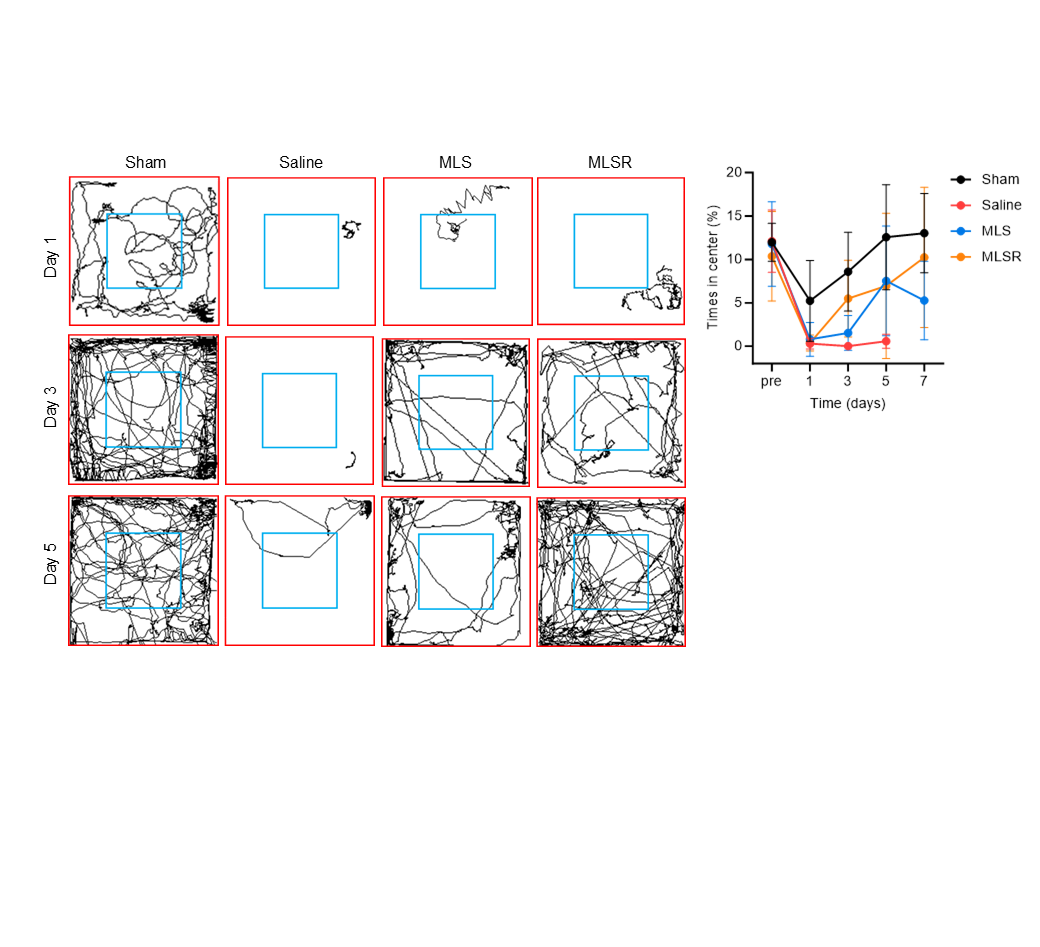


Figure S34. Representative images of open field tests at 1-, 3-, and 5-day post-stroke and analysis of time in the open field center (two-way ANOVA; n = 6 per group).


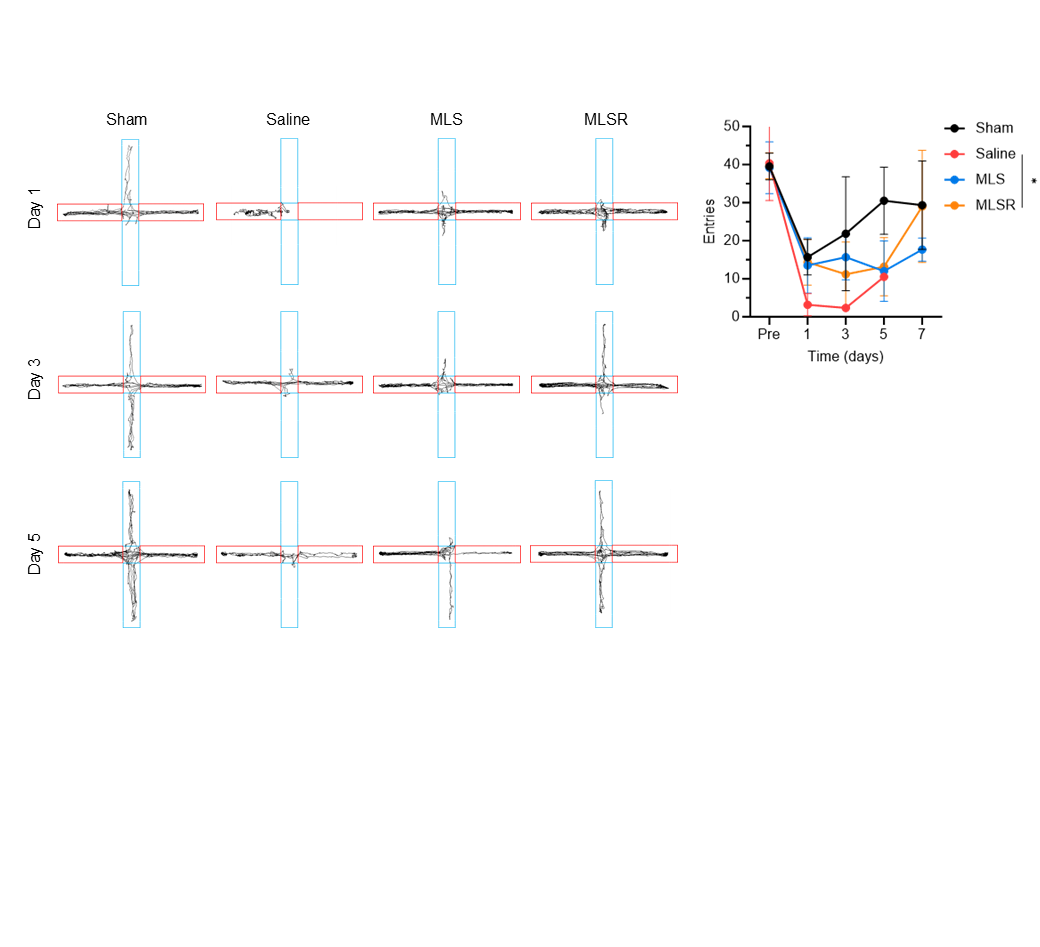


Figure S35. Representative images of elevated plus maze tests at 1-, 3-, and 5-day post-stroke, and analysis of entries in the open arms (two-way ANOVA; n = 6 per group).
